# Mechanism-First Psychobiotics: Fermented Vegetables, Dairy, and Soy for Depression and Anxiety

**DOI:** 10.3390/ijms27146399

**Published:** 2026-07-18

**Authors:** Masaru Tanaka, Claudia Rucco Penteado Detregiachi, Vitor C. Strozze Catharin, Eliana de Souza Bastos Mazuqueli, Cristiano Machado Galhardi, Tereza L. Menegucci Zutin, Mariana Hirata, Karina Quesada, Virginia M. C. Strozze Catharin, Rafael S. de Argollo Haber, Vitor Fernando Bordin Miola, Sandra Maria Barbalho

**Affiliations:** 1 Danube Neuroscience Research Laboratory, HUN-REN-SZTE Neuroscience Research Group, Hungarian Research Network, University of Szeged (HUN-REN-SZTE), H-6725 Szeged, Hungary; 2 Department of Biochemistry and Pharmacology, School of Medicine, Faculdade de Medicina de Marília, Universidade de Marília (UNIMAR), Marília 17525-902, SP, Brazilvitorfbmiola@gmail.com (V.F.B.M.); 3 Post-Graduate Program in Structural and Functional Interactions in Rehabilitation, School of Medicine, Universidade de Marília (UNIMAR), Marília 17525-902, SP, Brazil; 4 Research Coordination, UNIMAR Charity Hospital, Faculdade de Medicina de Marília, Universidade de Marília (UNIMAR), Marília 17525-902, SP, Brazil

**Keywords:** major depressive disoder (MDD), anxiety disorders, gut–brain axis, gastrointestinal microbiome, fermented foods, probiotics, short-chain fatty acids (SCFAs), indoles, bile acids, endothelial dysfunction

## Abstract

Depression and anxiety are increasingly understood to involve systemic biological processes, where chronic stress, immune dysregulation, and vascular dysfunction converge on brain-relevant symptoms. Fermented foods are widely studied as psychobiotic candidates, yet results remain inconsistent because products vary in chemistry, viability, sodium, and biogenic amines, and trials often rely on broad symptom outcomes without exposure verification. A major gap is the lack of a reusable, mechanism-first framework that links what a product delivers to barrier, endothelial, and neurovascular target engagement. As a narrative and conceptual review rather than a systematic review, the article integrates mechanistic evidence into a conceptual framework rather than undertaking quantitative evidence synthesis. It addresses that gap by treating fermented vegetables, dairy, soy, and selected Brazilian cassava ferments and artisanal cheeses as metabolite-engineering platforms mapped onto a tri-barrier remodeling axis from gut epithelium to endothelium and platelets to the blood–brain barrier. We synthesize dosing-resolved metabolite modules, including short-chain fatty acids, tryptophan-derived indoles, bile acids, neuroactive small molecules, and peptide and exopolysaccharide fingerprints, and align them with interpretable readouts for permeability, endotoxemia proxies, endothelial activation, immunothrombosis, and epigenetic aging pace. Here we highlight how this modular framework converts heterogeneous food studies into testable exposure hypotheses, guides comparator design and phenotype stratification, and clarifies why null results can be informative. To maintain a focused scope, the review uses selected fermented-food families as representative test platforms rather than attempting a complete survey of global fermented foods. The emphasis is therefore placed on mechanisms, exposure verification, and trial-design principles that can be transferred to other products.

## 1. Introduction

Depression and anxiety increasingly read as vascular inflammatory syndromes as much as brain-limited disorders, with chronic stress, hypothalamic–pituitary–adrenal (HPA) and autonomic imbalance, immune dysregulation, platelet activation, and endothelial injury converging on microvascular dysfunction and blood–brain barrier (BBB) vulnerability [1,2,3]. Population and clinical studies link depressive disorder to composite signatures of low-grade inflammation and endothelial activation, while mechanistic models implicate nitric oxide (NO) biology, nuclear factor kappa B (NF-κB) signaling, and kynurenine (KYN) pathway diversion as shared substrates of mood symptoms and cardiometabolic risk [2,4,5]. In parallel, psychobiotic research has shifted toward microbiome metabolites as actionable mediators [6,7]. This review therefore frames fermented foods as metabolite-defined interventions, motivates platform selection, and sets clear objectives for a mechanism-first synthesis [6,7,8].

### 1.1. Vascular-Inflammatory Phenotypes in Depression and Anxiety

Depression and anxiety include clinically relevant vascular-inflammatory phenotypes, in which chronic stress, hypothalamic–pituitary–adrenal and autonomic imbalance, immune dysregulation, platelet activation, and endothelial injury can converge on microvascular dysfunction and blood–brain barrier vulnerability [1,2]. In a remodeling-centered Special Issue, this motivates focusing on vascular substrates, especially the endothelium, as mediators that shape both brain-relevant inflammation and cardio–cerebrovascular outcomes [2,9]. Across clinical cohorts and population studies, mood syndromes frequently align with a vascular inflammatory phenotype in which low-grade immune activation runs in parallel with endothelial activation, platelet priming, and microvascular dysfunction [2,10]. In older adults, small-vessel brain disease and white-matter lesions track with late-life depression, supporting vascular depression models in which hypoperfusion and barrier vulnerability intersect with neuroimmune stress responses [2,11,12,13].

Endothelial activation and microvascular dysfunction provide a plausible mechanistic bridge between peripheral inflammatory tone and brain vulnerability, particularly in symptom profiles marked by fatigue, sleep disruption, and stress reactivity [2,14,15,16]. Meta-analytic and epidemiologic evidence links depressive symptoms to peripheral and cerebral microvascular injury, including impaired retinal reactivity, elevated endothelial biomarkers, white-matter hyperintensities, microbleeds, and infarcts [2,9,12]. Preclinical and experimental studies strengthen causal plausibility: chronic stress can remodel brain endothelium through vascular endothelial growth factor (VEGF) signaling and tight-junction disruption, while restoration of NO-dependent microvascular function can improve stress-related behavioral phenotypes in experimental models [17]. Human evidence, however, remains largely associative and supports the neurovascular unit as a testable mediator rather than a proven clinical causal pathway [18]. These experimental findings support mechanistic plausibility, but in humans they should be interpreted as translational support for the neurovascular unit as a testable mediator rather than as proof of a clinical causal pathway.

Immunothrombosis offers a convergent framework in which endothelium, platelets, and innate immunity form a coupled system that can influence cerebral perfusion, BBB integrity, and neuroimmune signaling relevant to mood [10,19,20,21]. Human data further suggest that mood symptoms track with vascular remodeling and a prothrombotic milieu in high-risk states such as post-myocardial infarction, where arterial stiffness and fibrinogen changes co-occur with anxiety and depression [10,22,23]. Taken together, the key implication for psychobiotics is methodological: interventions should be evaluated not only by symptom change but also by measurable engagement of barrier, endothelial, and thrombo-inflammatory pathways that define remodeling risk, ideally coupled to microbiome-metabolite readouts to support mechanistic inference rather than parallel association stories [6,8].

### 1.2. What “Mechanism-First Psychobiotics” Means (And What It Excludes)

“Mechanism-first psychobiotics” treats fermented-food interventions as mechanistically specified agents, defined by strain identity, quantifiable metabolite outputs, or reproducible chemical fingerprints, and anchored to demonstrable host target engagement [6,24,25]. This framing responds to a recurring limitation in the literature: taxonomy-heavy narratives and symptom-only trials often cannot distinguish causation from coincidence when closely related strains differ in bioactive capacity [6,24,26]. The definition shifts the field toward testable causal chains linking exposure to immune, endocrine, metabolic, and neuroactive pathways relevant to depression and anxiety [27,28,29,30].

In this framework, “psychobiotic” stops being a broad category term and becomes an operational designation that requires explicit strain or metabolite specification plus biomarker-based evidence that the intended pathway is actually engaged [24,29,31]. Fermented foods differ from both general “healthy diets” and capsule probiotics because fermentation generates new chemical entities and reshapes matrix bioaccessibility, altering when and where metabolites are encountered along the gut [6,24,26]. This distinction is mechanistically consequential. Immune receptors, endothelial programs, and neuroimmune mediators often respond to exposure kinetics, dose profiles, and sites of release as much as to the molecular identity itself. The effects of fermented foods cannot be inferred from food labels or taxonomy alone but must be interpreted through quantifiable exposure modules [24,26,32]. Extending this logic, translation of microbiome–gut–brain axis research into clinically meaningful strategies requires moving beyond capsule-based interventions toward real dietary exposures, where food matrices, fermentation chemistry, and metabolite kinetics jointly determine biological effects [6,26,33,34,35]. Fermented foods such as kefir, kimchi, and fermented soy products therefore function not as generic “healthy foods,” but as complex delivery systems that combine metabolite provision, substrate steering, and ecological modulation across the gut–vascular–brain axis [6,26,36].

To meet that standard, studies must show target engagement by quantifying in vivo metabolite modules and coherently linking them to barrier, endothelial, and inflammatory readouts that plausibly carry gut signals into vascular and brain vulnerability [6,27,28]. In practice, this means tracking tractable readouts such as short-chain fatty acids (SCFA) and tryptophan (Trp)–indole signatures alongside permeability markers, cytokine panels, and endothelial stress indicators, then mapping them onto symptom domains rather than global scores [27,37,38,39,40,41]. Without this, trials risk becoming interpretable only as behavioral or nutritional interventions rather than as mechanistic tests of microbiome-metabolite biology [6,26,28].

By the same logic, the approach explicitly rejects “more beneficial bacteria” rhetoric, culturally compelling food stories, or unquantified exposure descriptions as mechanistic evidence in the absence of metabolite-resolved pathways, confound control, and phenotype stratification [6,24,26]. Likewise, taxonomy-level claims or multi-strain blends blur strain specificity and dilute causal inference [24,29]. Instead, we prioritize metabolite-defined pathways, confound control (e.g., sodium and biogenic amines), and phenotype stratification to prevent false positives and false negatives in depression/anxiety outcomes [6,26,35].

### 1.3. Why These Three Product Families

The manuscript centers on three fermented-food platform families because they offer complementary mechanistic leverage: live fermented vegetables primarily reshape barrier ecology through organic acids, fiber-linked substrate flow, and locally produced microbial metabolites, while yogurt and kefir offer pragmatic trial feasibility with standardized comparators and a well-described payload of fermentation-derived peptides and exopolysaccharides (EPS) [6,26,36]. Fermented soy adds peptide-rich biochemical diversity and a distinctive capacity for biotransforming phytochemicals into more bioaccessible derivatives with plausible vascular and immune relevance [6,24]. Using these platforms in parallel allows the field to test whether distinct fermented matrices converge on shared downstream pathways, including endothelial activation, immunothrombosis, and neuroimmune tone, or whether effects remain platform-specific and phenotype-dependent [6,27,28]. This selection was deliberately narrowed to three product families with complementary mechanistic roles: fermented vegetables for barrier and ecology effects, fermented dairy for trial feasibility and comparator control, and fermented soy for peptide-rich and immunometabolic diversity. Brazilian cassava ferments and artisanal cheeses are retained only as regional case studies to test external validity, not as additional full review domains.

This platform set is intentionally representative rather than exhaustive, chosen to make mechanistic claims testable with feasible comparators and measurable exposure fingerprints [6,26]. Accordingly, important fermented foods are not treated as primary case examples here, including natto, kombucha, sourdough, fermented teas, traditional fermented fish sauces, and other regional staples [6,26]. These omissions reflect scope discipline, not a judgment about importance, and the framework is meant to be transferable to additional products when chemistry, confounds, and endpoints can be specified [6,26].

**Figure 1:**
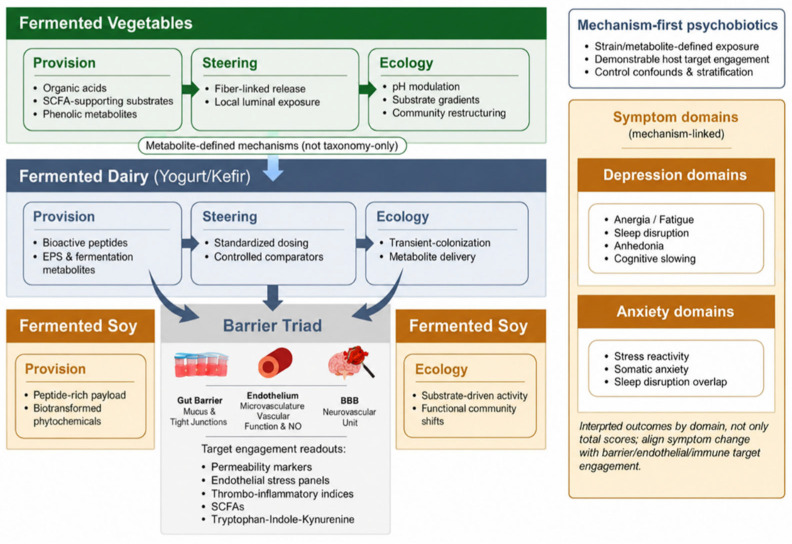
Product-centered psychobiotic framework. Three fermented food families, fermented vegetables, fermented dairy, and fermented soy, are presented as parallel lanes that convert product choice into mechanistic hypotheses and clinically interpretable outcomes. Each lane is decomposed into Provision, Steering, and Ecology. Provision summarizes dominant bioactive payloads and metabolite modules, such as organic acids, bioactive peptides, EPS, and biotransformed phytochemicals. Steering captures exposure kinetics and trial controllability, including dose standardization, comparator feasibility, and matrix-dependent bioaccessibility along the gut. Ecology reflects local pH shifts, substrate gradients, transient colonization, and functional community restructuring. Inputs from all lanes converge on a shared barrier triad consisting of the gut barrier, endothelium, and microvasculature, and the blood–brain barrier and neurovascular unit. Target engagement can be tracked via permeability markers, endothelial stress panels, thrombo-inflammatory indices, and SCFA plus Trp-indole-KYN signatures. Downstream effects are mapped to depression and anxiety symptom domains to support domain-level interpretation. BBB, blood–brain barrier; EPS, exopolysaccharides; SCFA, short-chain fatty acids; Trp, tryptophan. Figure prepared with Canva.com.

We additionally integrate Brazilian cassava ferments and artisanal cheeses as mechanistic case studies that sharpen the platform logic by highlighting fermentation-driven carbohydrate matrix remodeling and proteolysis-driven peptide ecosystems, respectively [6,26,42]. Soy anchors this extension because it couples peptide density with microbial biotransformation of phytochemicals, yielding aglycones and other derivatives with plausible immunometabolic and vascular relevance [6,24,43]. Across these examples, the key is not culinary identity but measurable effectors, including strain-resolved communities, peptide and EPS signatures, and small-molecule modules that map onto barrier and inflammatory biology [6,27,28]. This broadened platform set supports a more generalizable framework in which fermented foods are classified by quantifiable targets, enabling mechanism-first product selection, metabolite-defined pathway testing, confound control, and phenotype-relevant interpretation of depression/anxiety outcomes (Figure 1). The revised scope is therefore centered on a limited conceptual task: to define how selected fermented-food platforms can be evaluated through metabolite modules, barrier readouts, vascular endpoints, and phenotype-stratified depression and anxiety outcomes. Topics that would require independent reviews, such as global fermented-food diversity, all probiotic formulations, broad nutritional psychiatry, and detailed cultural food histories, are treated only when they support this mechanistic framework. To calibrate mechanistic inference, the review distinguishes three evidence levels. Established knowledge refers to findings supported by human clinical, epidemiological, or replicated mechanistic studies. Preclinical evidence refers to animal, cellular, organoid, ex vivo, or experimental models that define pathways but cannot alone establish clinical effects in depression or anxiety. Mechanistic plausibility refers to biologically coherent but incompletely tested links, especially when fermented-food trials have not yet verified exposure, target engagement, or phenotype-specific outcomes. This evidence-tier language is used throughout the revised manuscript to avoid presenting hypotheses as established facts. Accordingly, causal terms in the Introduction are reserved for experimental or preclinical contexts, whereas human cohort and clinical evidence is described as associative, convergent, or hypothesis-generating.

We additionally integrate Brazilian cassava ferments and artisanal cheeses as mechanistic case studies that sharpen the platform logic by highlighting fermentation-driven carbohydrate matrix remodeling and proteolysis-driven peptide ecosystems, respectively [6,26,42]. Soy anchors this extension because it couples peptide density with microbial biotransformation of phytochemicals, yielding aglycones and other derivatives with plausible immunometabolic and vascular relevance [6,24,43]. Across these examples, the key is not culinary identity but measurable effectors, including strain-resolved communities, peptide and EPS signatures, and small-molecule modules that map onto barrier and inflammatory biology [6,27,28]. This broadened platform set supports a more generalizable framework in which fermented foods are classified by quantifiable targets, enabling mechanism-first product selection, metabolite-defined pathway testing, confound control, and phenotype-relevant interpretation of depression/anxiety outcomes (Figure 1). The revised scope is therefore centered on a limited conceptual task: to define how selected fermented-food platforms can be evaluated through metabolite modules, barrier readouts, vascular endpoints, and phenotype-stratified depression and anxiety outcomes. Topics that would require independent reviews, such as global fermented-food diversity, all probiotic formulations, broad nutritional psychiatry, and detailed cultural food histories, are treated only when they support this mechanistic framework. To calibrate mechanistic inference, the review distinguishes three evidence levels. Established knowledge refers to findings supported by human clinical, epidemiological, or replicated mechanistic studies. Preclinical evidence refers to animal, cellular, organoid, ex vivo, or experimental models that define pathways but cannot alone establish clinical effects in depression or anxiety. Mechanistic plausibility refers to biologically coherent but incompletely tested links, especially when fermented-food trials have not yet verified exposure, target engagement, or phenotype-specific outcomes. This evidence-tier language is used throughout the revised manuscript to avoid presenting hypotheses as established facts. Accordingly, causal terms in the Introduction are reserved for experimental or preclinical contexts, whereas human cohort and clinical evidence is described as associative, convergent, or hypothesis-generating.

For practical grading, human randomized trials are treated as the strongest intervention evidence, observational studies as clinical association evidence, animal and cellular studies as preclinical mechanistic evidence, and metabolomic or vascular-biology extrapolations as mechanistic plausibility. Future trial recommendations are therefore presented as testable design priorities rather than as evidence that fermented foods already modify mood through a complete gut-endothelium-BBB pathway [3]. The operational minimum criteria for mechanism-first psychobiotic classification are summarized in Box 1.

Box 1 **Minimum criteria for mechanism-first psychobiotic classification.**

To keep terminology mechanistically honest, we propose the following minimum criteria. A candidate psychobiotic must be defined by strain identity, metabolite outputs, or a reproducible chemical fingerprint, not by food halo or taxonomy rhet-oric. It must demonstrate host target engagement using barrier and vascular readouts that matter for depression and anxiety framed as vascular inflammatory syndromes. At minimum, quantify metabolite modules such as short chain fatty acids and tryptophan indole kynurenine signatures, alongside permeability mark-ers, endothelial activation panels, and thrombo-inflammatory indices. Symptom change should be interpreted by domain and linked to these pathways.

## 2. Fermented-Food Platforms as Mechanistic Tools

Fermented foods can be viewed as experimental platforms that transform raw ingredients into reproducible exposure systems for probing how microbiome-derived metabolites influence vascular and neuroimmune biology [6,44,45]. Fermentation broadens the bioactive landscape by generating organic acids, peptides, exopolysaccharides, and biotransformed phytochemicals, while also introducing microbial communities capable of reshaping the gut microenvironment, barrier function, and immune and HPA-axis signaling relevant to depression and anxiety [6,44,46]. Since these effects depend strongly on both matrix and process, mechanistic interpretation requires precise product characterization and exposure measurement. Given the breadth of fermented-food diversity, this section focuses on a small set of representative platforms rather than attempting an exhaustive survey. This boundary is important because the aim is not to summarize every fermented food, but to build a reusable mechanism-first template. Products outside the selected families are mentioned only when they help define transferable modules, confounds, or future trial opportunities. As a result, widely studied products such as natto, kombucha, and sourdough are mentioned mainly for context, despite their clear relevance for future module-based trials. The section therefore proceeds by outlining a three-lever framework, defining the principal chemical consequences of fermentation, examining Brazilian products as illustrative case studies, and establishing the standardization principles needed to preserve mechanistic rigor.

### 2.1. The Three-Lever Framework (Use Throughout the Review)

Fermentation can be translated into testable psychobiotic biology through three mechanistic levers, each with distinct measurement demands and failure modes [29,47,48]. Metabolite provision is the most direct [27,47,49]. Fermentation-derived small molecules can engage host targets without requiring durable microbial colonization. Across the psychobiotic and fermented-food literature, the most reproducible candidates include organic acids and SCFAs, Trp-derived indole metabolites, and selected neuroactive intermediates that covary with inflammatory tone and stress biology in depression and anxiety [48,50,51]. Treating these as provided effectors tightens inference because the hypothesis becomes dose-linked and falsifiable: exposure must shift first, then intermediates and symptoms can be interpreted as downstream movement [27,49,50]. This lever is strongest when exposure is quantified, since kinetics determine whether metabolites plausibly reach immune, endothelial, or neurovascular interfaces [49,50,52].

Substrate steering acts upstream by reshaping the food matrix and therefore the substrates presented to the colon [29,47,53]. Acidification, partial degradation of fibers and proteins, reduction in antinutrients, and polyphenol biotransformation alter fermentability and bioaccessibility, shifting endogenous production of SCFAs, indoles, bile-acid derivatives, and other host-sensed metabolites [50,54,55]. Metabolomics reinforces this point: process conditions generate distinct small-molecule fingerprints that cannot be inferred from ingredient lists [29,54,56]. The mechanistic prediction is not that fermented foods contain beneficial metabolites, but that they shift metabolite outputs in vivo, often in a diet-dependent manner [47,51,57].

Ecology shaping extends the time horizon. Acids, microbes, and microbial effectors act as selection pressures that recalibrate community function and resilience, even when taxonomic changes look modest [47,52,57]. Fermented foods then operate less as one-off exposures and more as repeat perturbations that favor specific metabolic guilds, alter cross-feeding, and reshape barrier-facing and immune-active outputs relevant to mood [47,50,51]. This distinction makes durability a key criterion: transient passage may create short-lived functional shifts, whereas repeated exposure can stabilize functional behaviors without obvious compositional reorganization [51,52,57]. Because storage, viability, and co-ingredients influence this lever, claims should be anchored to functional readouts rather than compositional snapshots alone [29,47,58]. This ecological perspective also means that fermented foods should be interpreted within whole dietary patterns rather than as isolated items [47,51,57]. In real diets, background fiber availability, protein intake, and matrix co-exposures determine whether fermented foods amplify short-chain fatty acid production, indole signaling, and community resilience, or instead generate weaker, less reproducible effects [51,52,57]. Accordingly, ecological validity is not a soft nutritional add-on; it is part of the mechanism [59,60,61,62,63]. The product-family positioning statements are summarized in Box 2.

Box 2 **Product-family positioning statements (vegetables vs. dairy vs. soy).**

Fermented vegetables function as the barrier and ecology lever: organic acids plus fiber-linked substrate flow reshape luminal pH gradients, tighten gut-barrier tone, and reduce endotoxemia potential that can feed endothelial activation. Yogurt and kefir function as the trial lever: standardized comparators, consistent dosing, and tractable fermentation outputs, especially peptides and exopolysaccharides, make mechanistic target engagement testable. Fermented soy functions as the peptide and immunometabolic lever: proteolysis and phytochemical biotransformation yield bioactive diversity suited to vascular and inflammatory phenotypes.

### 2.2. Product-Centric Chemistry and “What Actually Changes”

Fermentation across vegetables, dairy, and soy reliably drives acidification, redox shifts, and remodeling of small molecules that redirect both microbial competition and host exposure [29,47,53]. Starter consortia channel carbohydrates into lactic and related acids while releasing acetate, propionate and butyrate precursors, volatile acids, and reactive intermediates such as peroxides and carbonyls [29,47]. Polyphenols shrink into more absorbable phenolic acids with altered activity [43,50,53,55]. These chemical shifts are mechanistically relevant because luminal pH and organic acid balance can modulate epithelial barrier function and inflammatory tone, with downstream effects on endothelial behavior [27,47,50].

Fermentation also rewires proteins and polysaccharides. Microbial proteases carve milk and plant substrates into peptide constellations whose sequences depend on strain and process, yielding antioxidant, angiotensin-converting enzyme (ACE) inhibitory, immune, and antithrombotic potential [50,64,65]. At the same time, lactic acid bacteria (LAB) secrete EPS that behave as postbiotic polymers [47,50,64]. They influence mucosal signaling, microbial ecology, and even texture through interactions with proteins [50,64]. Peptide and EPS patterns, therefore, act as product identities that are measurable and comparable, not vague promises attached to a food name [47,50].

Matrix context substantially changes biological interpretation. The same organism or metabolite may produce different effects depending on bioaccessibility, release kinetics, and co-occurring chemical exposures that govern target engagement [29,50,53]. Gastric handling and digestion then sculpt distinct exposure curves for acids, peptides, lipids, minerals, and phytochemicals [29,50,51]. Human metabolomics confirms that similar nutrient lists can yield divergent systemic footprints [29,54,56]. Plant tissues further complicate matters because bound compounds may appear late, primarily in the colon [29,53,55]. Mechanism-first trials must therefore lock down format and processing, while treating sodium and biogenic amines as active modifiers of effect.

### 2.3. Brazilian Fermented Platforms (Cassava and Artisanal Cheeses) as Mechanistic Case Studies

Brazilian cassava fermentations offer a mechanistically clean example of fermentation as matrix bioprocessing, where acidification and starch remodeling jointly shape downstream microbial metabolism and barrier-facing hypotheses [66,67]. More broadly, Brazil offers a diverse repertoire of cassava-, dairy-, and cereal-based ferments shaped by spontaneous or semi-controlled fermentation, underscoring how regional products can generate complex biochemical transformations with mechanistic relevance beyond globally standardized models [68,69,70,71]. To avoid widening the review beyond its central focus, these Brazilian platforms are not presented as a separate comprehensive regional review. They are used as stress tests for the proposed framework because they illustrate how local fermentation systems can be translated into measurable exposure fingerprints, safety variables, and barrier-to-vascular endpoints. The inclusion of Brazilian cassava ferments is intentional. They are retained as case-study platforms, not as additional review domains [70,71,72]. Across tucupi, puba or carimã, polvilho azedo, and farinha d’água, LAB and yeasts drive a rapid pH drop, generate organic acid profiles, and remodel carbohydrate structure and fermentability [66,67,70]. At the same time, traditional workflows also aim to reduce cyanogenic potential through pressing, washing, fermentation, and, for tucupi, boiling [70]. Omics and targeted analytics indicate that these platforms can carry process-sensitive signatures, including carotenoid shifts and low but detectable levels of biogenic amines, with substantial variability across commercial samples [72,73]. Tucupi, puba or carimã, polvilho azedo, and farinha d’água therefore serve as case-study platforms for testing substrate steering and barrier engagement, provided batch chemistry and potential amine or sodium variability are measured [72,73]. In addition to acidification and detoxification, these cassava platforms may alter starch structure in ways that increase colonic fermentability and support acetate- and butyrate-linked barrier and metabolic effects, although these outcomes remain strongly process- and microbiota-dependent [66,74,75,76,77].

Brazilian artisanal cheeses, such as queijo Canastra and queijo do Marajó, highlight fermentation as a proteolysis-dominant ecosystem capable of generating high-information peptide and EPS profiles [78,79,80]. They are included only as regional case studies showing how artisanal dairy matrices can be translated into peptidomic, metabolomic, safety, and comparator requirements [78,79,81]. Raw-milk technologies and endogenous starters create dense microbial consortia in which LAB and yeasts, shaped by geography, farm practices, and even phage–bacterium dynamics, drive ripening-dependent proteolysis and metabolite succession [78,79,81]. Consistent with this, ripening studies show time-dependent accumulation of water-soluble peptide fractions with antioxidant, ACE-inhibitory, and antimicrobial activities, while isolate screens identify frequent EPS-producing and biopreservative LAB traits [78,80,82]. Yet these platforms are high-variance and carry nontrivial safety constraints. Their mechanistic value is highest when coupled with peptidomic and metabolomic fingerprinting and sodium and biogenic amine reporting, enabling comparison with standardized yogurt-style platforms [79,80,83]. Their peptide repertoires also strengthen the case that artisanal cheeses may influence oxidative balance, inflammatory signaling, and vascular function, although this promise remains inseparable from the need for tight safety and batch standardization [80,82,84,85,86,87]. Regional and indigenous fermented preparations likely share these same core mechanistic themes—metabolite production, ecological modulation, and host immune interaction—even where formal psychobiotic evidence remains sparse [70,71]. In Table 1, the product platforms are shown.

### 2.4. Standardization and Safety That Directly Affect Mechanisms

Standardization is essential for mechanistic interpretation. Changes in viability, acid load, salt content, storage conditions, oxygen exposure, temperature, or fermentation time can alter the delivered mixture of organic acids, peptides, EPS, amines, and transformed phytochemicals. Without precise product definitions, dose–response interpretation becomes unreliable, and null findings cannot be assigned confidently to biological inactivity [103,104].

A minimum specification keeps inference alive. Report identity, preparation, serving size, viability status, pH, and a compact organic acid profile. Report whether the product is live or heat-treated, its age, storage conditions, and any factors known to influence metabolite drift. Safety chemistry should be reported within the same framework. Sodium and targeted biogenic amines must be visible, along with routine pathogen screening. If omics are deployed, analytical pipelines and quality thresholds should be explicit [41,105,106]. Where available, trial reports should also specify actionable reference ranges or predefined exclusion thresholds for sodium and biogenic amines, including histamine and tyramine [107]. Because regulatory limits differ across food categories and countries, these thresholds should be matrix-specific, justified from food-safety guidance or validated analytical literature, and reported as mg per serving, mg per day, or mg/kg product as appropriate [108]. When no universal threshold exists, investigators should define batch-release criteria in advance and apply them consistently across intervention and comparator arms [107].

Sodium and biogenic amines require explicit control because they can alter vascular tone, arousal, and sleep, thereby imitating benefit or harm [109]. Comparator logic should therefore match exposure variables rather than food names [108]. Align sodium, track amines, and design arms that can disentangle fermentation from parallel variables. More broadly, causal inference is also limited by product heterogeneity, dietary confounding, small sample sizes, and short intervention durations, which together make symptom-only findings particularly vulnerable to misinterpretation [45,91]. In Table 2, it is possible to find information about components in fermented foods.

## 3. The Tri-Barrier Remodeling Axis: Gut-Endothelium-Blood–Brain Barrier

The tri-barrier remodeling axis frames the gut epithelium, systemic endothelium, and BBB as interlocked control surfaces that convert luminal chemistry into vascular and neuroimmune states, shaping depression and anxiety [138,139,140]. Events upstream set boundary conditions downstream [138,141,142]. A shift in permeability or mucosal signaling can recalibrate cytokine tone, alter endothelial behavior, and ultimately influence neural vulnerability [138,139,143]. Apparent inconsistencies across fermented foods become predictable when exposure kinetics, matrices, storage conditions, and comparators are misaligned, because each variable shifts where and how the signal lands [142,143]. A mechanism-first strategy therefore measures the interfaces in sequence rather than chasing symptoms [138,142,143]. The roadmap proceeds from epithelial sensing to vascular activation to cerebral gating, while aligning vegetables, dairy, and soy with the barrier tier they most plausibly influence and, thus, must be tested. Accordingly, the following sections use calibrated language. Human clinical or epidemiological findings are described as associations unless intervention or longitudinal evidence supports stronger inference. Animal, cellular, and BBB-model findings are described as preclinical mechanisms. Product-to-barrier links are framed as testable hypotheses when direct fermented-food evidence is limited.

### 3.1. Gut Barrier: Pattern-Recognition Receptor (PRR) Signaling, Tight Junctions, Mucosal Immunity

Pattern recognition receptors at the epithelial frontier act as the central regulatory interface for the barrier network [141,144,145]. They convert contact with microbes into alarm signals, cytokine gradients, and recruitment cues for myeloid cells [144,145,146]. If that sensing skews toward danger, endotoxin tone lingers, repair falters, and vascular beds downstream inherit a primed phenotype [140,141,143]. The decisive experiment is therefore exposure biology, not taxonomy [144]. A mechanism-first design should determine whether receptor-relevant ligands decrease and whether downstream signaling is attenuated. Markers such as lipopolysaccharide (LPS)-binding protein (LBP), soluble CD14, Toll-like receptor-induced cytokines, and inflammasome products sit closest to ignition and define whether immune traffic has truly slowed [143,147,148].

Once alarms sound, junctions determine spread. Claudin pores and inducible leak pathways respond to inflammatory kinases, reorganizing occludin and zonula occludens 1 (ZO-1) and allowing graded antigen passage into tissue and blood [143,147,149]. Barrier dysfunction should be interpreted as a graded process rather than as a binary state [142,143,147]. Small defects can have large consequences for cytokines, keeping innate cells on patrol and perpetuating signals that later masquerade as primary brain pathology [140,141,143]. Mechanistic inference strengthens when symptoms travel with measures of permeability adjacent to the gut, such as zonulin-related signatures, intestinal fatty acid-binding protein, and direct assessments of junctional architecture across experimental systems [147,150,151].

Mucus provides the staging ground. Goblet cells invest energy to maintain distance between microbes and epithelium, a task reinforced by butyrate and indole chemistry that supports MUC2 expression and tolerance programs [141,152,153]. Diets that favor mucin degraders collapse that buffer [141,152]. Bacteria approach, receptors engage, and endotoxin pressure rises [141,144,152]. Immune governance then chooses containment or escalation [141,146]. Secretory IgA and antimicrobial peptides can quarantine exposure, yet persistent NF-κB bias drives interleukin (IL)-1β, IL-6, and T helper 17 cell (Th17) polarization [141,144]. Tracking IgA patterns, regenerating islet-derived protein 3 family (Reg3) activity, and Treg balance clarifies whether upstream sparks are extinguished or continue to fuel systemic flow toward vascular and neural interfaces [141,144,152].

### 3.2. Endothelium: Activation (Intercellular Adhesion Molecule (ICAM)/Vascular Cell Adhesion Molecule (VCAM)), Glycocalyx Loss, Nitric Oxide (NO) Biology

Endothelium converts diffuse inflammation into movement. Cytokines and danger signals drive cells toward adhesion, leak, and trafficking programs, characterized by intercellular adhesion molecule 1 (ICAM-1), vascular cell adhesion molecule 1 (VCAM-1), and selectins [154,155,156]. This pivot choreographs rolling, arrest, and transmigration while altering permeability and regional oxygen delivery [154,157,158]. Small molecular nudges can therefore magnify into perfusion deficits and self-reinforcing inflammatory loops [158,159]. Mechanism-driven trials should treat soluble and surface adhesion molecules, along with chemokine gradients, as scaling factors of vascular stress [155,156]. In brain-facing territories, the same biology shapes the susceptibility of microvessels that nourish circuits governing affect, motivation, and cognition [15,27,160].

Immunothrombosis shows what happens next. The endothelial stage recruits platelets and neutrophils, translating tone into obstruction [161,162]. Glycocalyx erosion removes anticoagulant protection, unmasks adhesion receptors, and weakens shear-dependent NO signaling [158,162]. These changes increase endothelial adhesiveness and promote platelet and leukocyte capture. Von Willebrand factor (vWF) release, P selectin exposure, and tissue factor activity intensify capture, while extracellular traps stabilize clots and concentrate enzymes that injure nearby cells [161,163,164]. Measuring vWF handling, fibrinogen, platelet leukocyte aggregates, and neutrophil extracellular trap (NET) products alongside adhesion markers turns an abstract risk into a trackable mechanism with therapeutic leverage [161,163,164].

Loss of glycocalyx is not decorative damage. Heparan sulfate-rich structures normally sense flow and relay it to endothelial nitric oxide synthase (eNOS), sustaining dilation and quelling leukocyte contact [159,165,166]. Oxidative stress and complement disturb this conversation. As the layer thins, permeability rises, and procoagulant motifs dominate [158,162]. Biomarkers such as syndecan-1 or hyaluronan help determine whether improvement reflects true endothelial repair or merely quieter upstream inflammation [158,162]. Pair them with functional tests, and the picture sharpens [165,166].

NO then decides performance. Adequate substrate and redox balance favor relaxation, efficient exchange, and coordinated neurovascular coupling [154,159,165]. Depletion of tetrahydrobiopterin, accumulation of asymmetric dimethylarginine (ADMA), or excess reactive oxygen species (ROS) diverts eNOS toward superoxide production, reducing bioavailability and stiffening the circuit [154,165,166]. Fatigue, stress sensitivity, and cognitive fog become plausible vascular consequences [160,166]. Because diet-derived metabolites can influence arginase flux and oxidative tone, trials gain power when NO metabolites are linked to redox indices and measures of vascular reactivity [154,159,165].

The tri-barrier model should be interpreted as an upstream vascular and immune framework, not as direct evidence that fermented foods modify human serotonin, dopamine, kynurenine tone, or BDNF signaling in the brain [6]. A more conservative interpretation is that barrier integrity, endotoxin tone, endothelial activation, oxidative stress, and BBB vulnerability may shape the inflammatory context in which these neural systems operate [167]. This link remains indirect unless trials include time-aligned metabolite exposure, endothelial or BBB readouts, and independently replicated neuroimmune markers [168]. Accordingly, claims about serotonin-kynurenine balance, dopaminergic function, HPA-axis regulation, and BDNF-related plasticity should be supported by independent primary or review sources and framed as translational hypotheses rather than demonstrated central effects of fermented foods [6].

### 3.3. BBB: Permeability, Microglial Priming, Stress Circuitry

The blood–brain barrier is best viewed as the brain’s specialized endothelium, extending systemic vascular logic into neural territory [169,170,171]. Claudin-5, occludin, and ZO scaffolds create high electrical resistance, reinforced by VE cadherin, while solute carriers and ABC efflux pumps govern selective entry under conditions of normally low transcytosis [172,173]. Inflammation and metabolic stress breach this control through two distinct routes [170,172,174]. Junctions can be relocalized or cleaved, but exposure can also shift without obvious leak when vesicular trafficking rises, and transporter programs are rewired [172,174,175]. For mechanism-first inference, BBB integrity must be measured directly, using permeability proxies and barrier-linked molecular signatures rather than assuming peripheral changes equal brain delivery [13,43,170,175,176,177]. Peripheral biomarker shifts are therefore treated as upstream indicators unless paired with direct BBB, neurovascular, or CNS-adjacent evidence [178]. This distinction is essential because reduced systemic inflammation does not automatically demonstrate altered brain exposure or circuit-level target engagement [179].

Once gating loosens, microglia become the amplifier. Perivascular microglia can initially stabilize vessel contacts and junctional proteins, then pivot toward breakdown, releasing ROS, IL-1β, tumor necrosis factor alpha (TNF-α), matrix metalloproteinase 9 (MMP-9), and chemokines that disrupt astrocytic endfeet, pericyte control of flow, and synaptic homeostasis [180,181,182,183]. That primed state makes later stressors disproportionately potent, translating modest peripheral inflammation into larger circuit-level effects relevant to depression and anxiety [14,181,184]. Pairing BBB readouts with microglial activation markers and inflammatory mediators clarifies whether an intervention strengthens interfaces upstream or only dampens downstream neuroimmune reactivity [13,181,182,185,186,187].

Neurovascular remodeling is not uniform. Limbic and prefrontal circuits gate HPA output differently, so identical blood signals can yield distinct endothelial and junctional responses across medial prefrontal cortex, hippocampus, amygdala, and nucleus accumbens [14,184,188]. Claudin 5-centered patterns, hypoperfusion, VEGF tone, and local cytokines repeatedly track susceptibility versus resilience [14,169,170]. Aligning mechanistic endpoints with symptom domains such as fatigue, sleep disruption, and stress reactivity turns heterogeneity into interpretable biology and guides which barrier layer an intervention most plausibly engages [14,184,188].

### 3.4. Product Mapping: Which Fermented Foods Most Plausibly Support Which Barrier Layer

Fermented vegetables are best framed as gut-barrier candidates because acidity, fiber-associated substrate steering, and LAB-driven ecology may influence endotoxemia-linked signaling upstream of endothelial activation. This mapping should be supported by direct fermented-food studies wherever available, including evidence for permeability markers, tight-junction proteins, SCFA or indole profiles, inflammatory readouts, or endotoxemia-related markers. General BBB literature should be retained only as background for biological plausibility, not as primary support for fermented-vegetable-specific effects [181,183]. This product-to-barrier mapping should be interpreted as provisional when direct fermented-food intervention evidence is limited [189]. General barrier or BBB literature is used here to define biological plausibility, whereas product-specific claims require direct evidence from fermented-food studies using permeability, tight-junction, endotoxemia, inflammatory, or endothelial readouts [6].

Kimchi and related fermented vegetables are best framed as candidate upstream modulators of gut barrier function rather than as established modifiers of BBB integrity or central neurotransmission [190]. Their mechanistic rationale is strongest when supported by product-specific evidence showing effects on epithelial integrity, SCFA or indole profiles, inflammatory tone, endotoxemia-related markers, or endothelial activation [191]. Any proposed downstream effects on kynurenine metabolism or serotonergic signaling should therefore be described as indirect hypotheses that require confirmation through aligned metabolite, barrier, endothelial, and neuroimmune readouts [192].

Fermented dairy and soy products extend the mapping from barrier containment to systemic signaling, because dairy is a trial-friendly matrix rich in peptide and EPS effectors, while soy adds peptide diversity plus biotransformed phytochemicals that intersect with immunometabolic pathways [175,183]. Across yogurts, kefir, and fermented whey, defined starters and processing can reproducibly tighten junctional programs, raise IgA- and Treg-linked tone, and lower cytokine spillover, making peptide and EPS fingerprints plausible effectors rather than background noise [175,183].

Kefir is a particularly useful trial-facing dairy platform because its symbiotic consortium, kefiran-rich matrix, and reproducible fermentation outputs create a tractable combination of peptides, exopolysaccharides, organic acids, and low-level fermentation byproducts [175,183]. Experimental and early clinical data suggest that these outputs may reduce inflammatory tone and oxidative stress while preserving endothelial nitric oxide bioavailability and microvascular function [175,181,183]. In neurobiological terms, that profile makes kefir especially relevant to symptom domains such as fatigue, stress reactivity, and anhedonia, where immune, vascular, and limbic mechanisms converge [14,181,184].

Soy ferments, exemplified by fermented soymilk in colitis models, similarly restore occludin and SCFA profiles but layer on remodeled isoflavones and other small molecules that can engage bile-acid, AhR, and metabolic signaling [183]. The practical implication is to choose platforms based on the dominant hypothesis: dairy when immune tone and peptide/EPS fingerprints are central, soy when metabolite diversity and immunometabolic signaling are prioritized, and then test both against shared endothelial and BBB readouts to establish barrier-to-brain mechanistic continuity [14,181,183,193].

Fermented soy products add a complementary immunometabolic layer because fermentation increases isoflavone bioavailability and generates peptide-rich profiles with antioxidant and ACE-inhibitory properties [62,183,194]. Through these effects, soy ferments may influence vascular tone, bile acid signaling, aryl hydrocarbon receptor biology, and systemic inflammatory load, making them especially relevant in depressive phenotypes coupled to metabolic dysfunction [14,181,183]. In that sense, fermented soy can be positioned as a bridge matrix linking endocrine, vascular, and neuroimmune regulation [14,181,183,193].

## 4. Metabolite Modules (Core Mechanistic Engine)

Metabolite modules are the working units of mechanism in fermented food research: they function like dosing readouts, capturing conserved receptor-facing chemistry that can be quantified, compared across products, and aligned with causal intermediates [44,64]. This module’s first stance matches modern psychiatric metabolomics, where pathway ensembles explain variation in depression and anxiety far better than single analytes or broad diet labels [195,196]. Across cohorts and experimental systems, recurrent clusters point to gut-derived co-metabolites and immunometabolic hubs—most notably SCFAs, Trp derivatives, and bile acid signaling—which then shape barrier integrity, endothelial activation, and higher-order integrative endpoints such as immunothrombosis and biological aging pace [197,198]. Accordingly, each subsection treats a module as a testable exposure unit with explicit kinetics, confounds, and mapping to shared gut, vascular, and BBB panels [38,199]. Across all modules, product-level, fecal, or luminal increases are treated as exposure evidence rather than proof of systemic delivery, target engagement, brain exposure, or clinical mechanism. Stronger inference requires aligned sampling across compartments, time-resolved kinetics, and pathway-specific host readouts. We begin with SCFAs because they are the most conserved fermentation to host signal and the simplest to track longitudinally.

**Figure 2:**
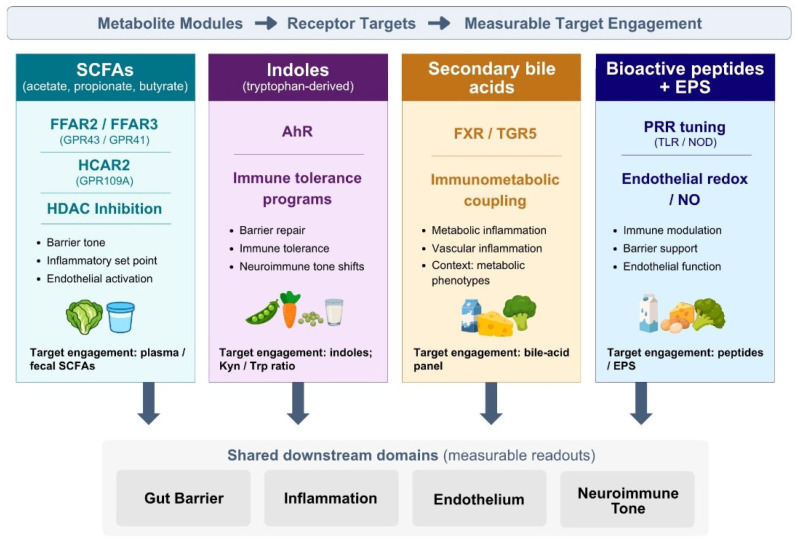
Metabolite modules and receptor targets linking fermented-food platforms to vascular and brain-relevant pathways. This schematic organizes fermented-food exposures into four mechanistically tractable metabolite modules and their primary host targets, emphasizing measurable target engagement rather than taxonomy or tradition. The SCFA module (acetate, propionate, butyrate) signals through FFAR2/FFAR3 and HCAR2 and can modulate chromatin via HDAC inhibition, shaping barrier tone, inflammatory set points, and endothelial activation programs. Indole derivatives arising from Trp metabolism engage AhR to bias immune tolerance and barrier repair, with downstream relevance to neuroimmune tone. Secondary bile acids act through FXR and TGR5 to couple immunometabolic state to vascular inflammation, making them particularly informative in metabolic comorbidity phenotypes. Bioactive peptides and EPS interact with immune pattern-recognition and endothelial nodes, supporting redox and NO-related plausibility. Product-family icons indicate the most likely drivers for each module (vegetables for SCFAs and indoles, dairy for peptides and EPS, soy for peptides and bile-acid-linked shifts), while acknowledging cross-platform overlap. AhR, aryl hydrocarbon receptor; EPS, exopolysaccharides; FFAR2, free fatty acid receptor 2; FFAR3, free fatty acid receptor 3; FXR, farnesoid X receptor; HCAR2, hydroxycarboxylic acid receptor 2; HDAC, histone deacetylase; NO, nitric oxide; SCFA, short-chain fatty acid; TGR5, Takeda G protein-coupled receptor 5; Trp, tryptophan. Figure prepared with Canva.com.

Figure 2 presents the metabolite modulation and receptor targets linking fermented-food platforms to vascular and brain-relevant pathways.

### 4.1. Short-Chain Fatty Acids (SCFAs): G Protein-Coupled Receptor (GPCR) + Histone Deacetylase (HDAC) Biology

SCFAs are best treated as dosing-grade concentration time exposures, not as a vague badge of “healthy fermentation” [200,201,202]. Fiber and resistant starch set the production rate, baseline guilds and cross-feeding set the acetate:propionate:butyrate pattern, and transit determines where along the colon each peaks [200,203,204]. Host handling then filters the signal: colonocytes avidly oxidize butyrate, propionate is largely routed to the liver, and acetate reaches peripheral beds more readily [200,205,206]. A fecal rise, therefore, is not the same as systemic delivery, and timing can matter even more than magnitude in vivo [200,201,202]. This creates a clean causal question in trials: are SCFAs primarily provided by the product’s chemistry, or produced because the intervention steers substrates toward saccharolytic metabolism? The answer determines what “dose” means, what compartment matters, and what should be measured [200,201]. Brazilian cassava ferments offer a clean SCFA-facing case because they are starch-dominant platforms where fermentation changes fermentability and acid load more than it “adds microbes” [207,208,209]. Tucupi and puba/carimã, for example, primarily test whether a process-defined carbohydrate matrix can shift fecal acetate–propionate–butyrate patterns under a fiber-controlled background [207,208]. Because cassava workflows also target cyanogenic risk reduction, chemistry release criteria should include organic acids, along with a safety-focused processing marker set, before inferring physiology [208,209,210].

Mechanistically, SCFAs translate fermentation into rapid mucosal decisions through FFAR2 (GPR43), FFAR3 (GPR41), and G protein-coupled receptor 109A (GPR109A) [211,212,213]. These GPCRs tune cAMP and Ca2^+^ signaling, bias mitogen-activated protein kinase (MAPK) and NF-κB programs, and reshape chemotaxis, enteroendocrine outputs, and cytokine tone across epithelial and myeloid compartments [211,214,215]. Butyrate, and to a lesser extent propionate, also enter cells via monocarboxylate transporters and inhibit HDACs, changing chromatin accessibility and slowing inflammatory transcriptional cycles [211,214,215]. These processes should be interpreted as a connected exposure-to-response sequence: speciation and kinetics set receptor engagement and intracellular exposure, which then reweight IL-10 and IL-22 support, restrain IL-6 and TNF-α, and favor Treg-competent programs [212,214,215]. The goal is not to promise effective rescue, but to demonstrate receptor biology that can plausibly propagate [212,213]. Stress physiology follows the immune set point, not the other way around [206,212]. Mood outcomes should be interpreted as downstream clinical readouts, whereas mechanistic target engagement should be evaluated closer to receptors, transporters, and chromatin-level signaling [212,214,215].

The barrier gate is where SCFAs become causally interpretable [213]. By supporting mucus energetics, tight-junction organization, and epithelial repair programs, SCFAs reduce the probability that luminal products will reach lamina propria sensors and the bloodstream [212,213]. A mechanism-first study therefore expects coordinated movement in containment markers before symptoms: improved TEER or reduced fluorescein isothiocyanate (FITC)-dextran flux in models, tighter junctional localization of claudins, occludin, and ZO-1, and, in humans, lower permeability-adjacent signals such as intestinal fatty acid-binding protein (I-FABP) or zonulin-linked signatures [212,213]. Add a simple immune traffic panel: LBP and soluble cluster of differentiation 14 (sCD14) for translocation pressure, plus a cytokine pattern consistent with reduced PRR drive [206,212]. If possible, include fecal calprotectin or a mucus marker to disambiguate epithelial injury from immune activation over a meaningful time, and assess whether increases in branched-chain fatty acids indicate a shift toward proteolytic fermentation, using sampling aligned with SCFA kinetics [205,211,212].

Once containment improves, endothelial biology becomes the vascular hinge that converts inflammatory tone into perfusion stability, thrombosis propensity, and aging-like programs [206,216]. SCFAs can dampen LPS- or TNF-α-driven endothelial activation by reducing NF-κB and MAPK outputs, often with lower ICAM-1, VCAM-1, and E-selectin signaling and fewer adhesive encounters [214,215,216]. In parallel, redox shifts preserve NO bioavailability by limiting ROS scavenging and supporting eNOS coupling, both of which are important for flow and neurovascular reserve [206,212]. The measurable prediction is a convergent phenotype: reduced adhesion programs, improved NO and oxidative balance, and less platelet priming that otherwise couples to NET formation, vWF handling, and microthrombi [212,216]. Pair adhesion markers with a platelet activation index to show translation from inflammation into vascular mechanics [216]. In that framing, vascular stabilization is a plausible substrate for reduced fatigue, steadier sleep, and less stress reactivity even before mood scales move [206,212].

Minimum specification should be strict [201,217]. Fingerprint the product with pH plus an organic-acid panel, then quantify stool SCFA speciation and pair it with plasma levels or postprandial sampling to capture kinetics [201,202]. Standardize pre-analytics: stool mass, rapid stabilization, cold chain, and zero tolerance for freeze–thaw drift [201]. Match comparators for calories, fiber grams, and type, and sodium, and record alcohol, antibiotics, and metformin because they reroute fermentation [200,203]. Start with SCFAs because they are the most conserved fermentation-to-host signal and the easiest module to quantify longitudinally [203,212,213] (Table 3). Once energy and barrier containment are anchored, the next module asks a different question: how does fermentation reroute Trp signaling toward immune tolerance or neuroinflammatory drift?

### 4.2. Tryptophan (Trp)/Indole Axis: Aryl Hydrocarbon Receptor (AhR) Signaling and Neuroimmune Gating

Trp is best treated as an allocation problem, not a nutrient tally [225,239,240,241]. The same dietary tryptophan pool can be routed toward microbial indole derivatives that support tolerogenic signaling, toward host kynurenine-pathway metabolism associated with immune activation, or toward serotonin precursors with gut and platelet relevance [4,225,239,242,243,244]. Fermentation and background diet influence this routing by altering substrate availability, redox conditions, microbial composition, and enzyme activity [245,246]. For mechanism-first work, the exposure unit is therefore a metabolite module, an interpretable mixture whose concentration and timing can be tracked across compartments rather than inferred from a food label [225,247,248]. This framing turns Trp effects into testable hypotheses about where flux goes and why, before any symptom story is told [16,240,244,246,249,250,251].

At the gut interface, indole-class ligands act as a practical switchboard because they can engage aryl hydrocarbon receptor (AhR) programs that stabilize containment [225,239,240]. When indole-3-aldehyde, indole-3-lactic acid (ILA), indole-3-acetic acid (IAA), or indole-3-propionic acid (IPA) rise, the predicted direction is not mood improvement but quieter immune traffic: tighter junction architecture, reinforced mucus and epithelial stress defenses, and stronger IL-22 centered repair and antimicrobial tone [239,240,252]. Those outputs matter because they reduce the probability that PRR sensing is repeatedly tipped toward alarm [239,240,253]. In other words, AhR signaling helps convert luminal chemistry into tolerogenic set points, with downstream effects on Th17 versus Treg balance and IgA mediated quarantine [239,240,253]. This is why barrier and mucosal immunity belong in the same paragraph as indoles: they are the mechanistic gate that decides whether signals stay local or spill systemically [239,240,254].

Neurovascular relevance follows the same logic, just in a different anatomical compartment [249,254,255]. If indole-driven containment reduces endotoxin and cytokine drift, the BBB and the neurovascular unit receive fewer priming cues that would otherwise bias microglia toward exaggerated responses to stress or infection [249,254,255]. Some indole-class ligands can also act more directly, shaping AhR-dependent inflammatory programs in microglia and astrocytes, which then modulate junctional support, chemokine tone, and synaptic vulnerability [249,254,255]. The key point is continuity: BBB gating is an extension of systemic endothelial biology, so shifts in indole modules should be read alongside permeability proxies and vascular activation, not in isolation [248,249,254]. That design makes it possible to distinguish upstream containment from downstream neuroimmune dampening, and to time-stamp where the signal first appears [248,249,254].

Minimum measurement is straightforward but non-negotiable [225,240,248]. Define dose as a concentration time profile, captured by time-resolved stool or fecal water indoles plus urine and plasma panels that report absorption and systemic handling [225,240,254]. A compact targeted set can include Trp, KYN, and the KYN-to-Trp ratio, plus indole, IAA, IPA, ILA, indole-3-aldehyde, and tryptamine, ideally with a parallel functional readout, such as AhR reporter activity or CYP1A1 induction [225,248,256]. Sampling should be aligned to product kinetics because matrix and processing change where and when ligands appear, and because colon production and host clearance are not synchronized [256]. Pair this with early downstream markers that should move first, such as IL-22, antimicrobial peptide surrogates, and barrier-adjacent readouts like zonulin-linked signatures or I-FABP, so attribution does not depend on symptoms [239,240,252].

Failure modes are predictable and preventable [240]. High protein load, low fiber context, or antibiotic and metformin exposure can reroute flux and flatten signals, while uncontrolled fermentation can add confounding amines and acids that change sleep and arousal [240]. The antidote is comparator discipline and diet context reporting, plus prespecified thresholds for what counts as a meaningful module shift, not just a *p*-value [240,245]. If indoles tune immune set points, bile acids act like an endocrine signaling interface, translating microbial chemistry into host metabolism and vascular tone [248,252,254].

### 4.3. Bile Acid Signaling: Farnesoid X Receptor (FXR)/Takeda G Protein-Coupled Receptor 5 (TGR5) Immunometabolic Bridge

Bile acids belong in a gut–brain translation stack because they link microbial chemistry with immune regulation, metabolic signaling, and vascular tone [257,258,259]. Gut microbes deconjugate and remodel primary bile acids into secondary species with different receptor potencies, so the true exposure is bile acid speciation, not “fat intake” or taxonomy [260,261,262]. These species can tune epithelial junctions, mucus and antimicrobial programs, bias macrophage cytokine output, and reshape glucose handling, which then modulates endothelial activation and perfusion stability [263,264,265]. If mood outcomes drift, this upstream chemistry may be the quiet driver, as it alters inflammatory load, energy balance, and vascular reactivity long before symptoms are read out in questionnaires [257,259].

Mechanistic interpretation is cleanest when it is anchored to two receptor backbones [258,261,264]. Intestinal FXR links luminal bile acids to transcriptional programs that regulate barrier maintenance, antimicrobial tone, and the gut–liver relay via fibroblast growth factor 19 (FGF19), with downstream effects on bile synthesis, lipids, and insulin sensitivity that influence vascular risk [261,264,266]. FXR is also immunomodulatory: in macrophages and innate lymphoid cells, it can suppress inflammatory transcription when ligand tone is favorable [263,264]. In parallel, Takeda G protein-coupled receptor 5 (TGR5) functions as a bile acid-sensing GPCR that raises cAMP, dampens NF-κB-driven macrophage activation, and promotes enteroendocrine glucagon-like peptide-1 (GLP-1) release, adding an incretin lever that can improve endothelial NO signaling indirectly through metabolic relief [264,267]. Treat these pathways as directional hypotheses: species that strengthen FXR feedback or engage TGR5 should track with lower inflammatory load and a less adhesive endothelium [263,264].

Minimum measurement should therefore be chemistry-first and time-aware. Quantify stool and plasma primary and secondary bile acids, plus key conjugates, using targeted liquid chromatography–mass spectrometry (LC-MS) panels that include cholic acid (CA), chenodeoxycholic acid (CDCA), deoxycholic acid (DCA), lithocholic acid (LCA), ursodeoxycholic acid (UDCA), and representative glycine or taurine conjugates [260,262,268]. Report ratios that map onto microbial function and receptor tone, such as secondary to primary bile acids—DCA:CA, LCA:CDCA—and conjugated to unconjugated pools, and sample with standardized timing around meals to capture enterohepatic oscillations [260,269,270]. Add a simple “deconjugation index” and record stool pH and Bristol scale, since transit and acidity shift pools independently of the intervention [262,270]. Define success as coherent directionality, not any movement: bile acid shifts should align with reduced PRR tone and barrier leak, improved inflammatory profiles, and vascular quiescence markers [258,263,264]. Pair speciation with FGF19 for FXR and GLP-1 for TGR5, then interpret against cardiometabolic context (homeostatic model assessment of insulin resistance (HOMA-IR), triglycerides, adiposity, and liver enzymes) that can otherwise masquerade as “psychobiotic” biology [257,264,266].

Bile acids cannot prove causality on their own because the same signature can reflect diet fat quality, transit time, drugs, or baseline microbiome capacity [257,258]. That is why the module needs paired intermediates: barrier markers, cytokine balance, and endothelial activation readouts that confirm the predicted chain [260,263,264]. Control protein and fat background, record antibiotics, metformin, and bile acid binders, and avoid over-reading single time points or isolated ratios [257,258,259]. With these guardrails, bile acids become a disciplined endocrine readout inside a broader mechanistic stack [257,259,265]. After endocrine mediators, the next step is discipline: neuroactive claims only count when exposure is measurable and compartment-aware. For this reason, a product-level or fecal increase in GABA, serotonin-related precursors, or other neuroactive compounds is classified as exposure evidence, not proof of systemic delivery, brain exposure, receptor engagement, or clinical mechanism.

### 4.4. Neuroactive Compounds and Neuromodulatory Pathways (Disciplined)

Neuroactive claims in fermented foods are useful only when posed like pharmacology: which molecule, what concentration-time curve, and which receptor or circuit are plausibly reached [243,250,271,272]. “Contains GABA” or “boosts serotonin” is not a mechanism if delivery is unmeasured and compartments are ignored [271,273,274]. Many candidates are produced in the jar but consumed, degraded, or trapped in the gut lumen, while expectancy and diet co-changes can mimic benefit [271,275,276]. Even a real peripheral shift may matter only through vascular and immune gates that set brain exposure over days, not minutes [129,199,277]. A disciplined approach, therefore, treats neuroactive effects as downstream readouts of quantified exposures and intermediates, not as headlines [271,275,276]. First, verify the module that moves, then ask whether barrier, immune, and HPA indices shift in the same direction and on the same timescale [129,199,277]. The same interpretive rule applies to all neuroactive candidates: product or fecal abundance alone should not be equated with plasma exposure, BBB passage, receptor engagement, or central neuromodulation.

What is plausible, then? Start with signals that have conserved exposure logic and peripheral gatekeeping power: SCFAs that tune inflammatory gain and vagal signaling through mucosal receptors, and Trp outputs that partition between indole ligands and KYN pathways, indexing tolerance versus immune activation [129,199,277]. Add a short list of fast, testable small molecules: GABA when strain screening plus product fingerprinting shows meaningful accumulation and stability; histamine and tyramine as dual-purpose analytes because they can be bioactive yet also confound sleep, anxious arousal, and vascular tone; and a few intermediates that repeatedly track with stress biology, such as lactate, succinate, and select catecholamine-related precursors [273,278,279]. The confound logic is central: amines, sodium, caffeine, and alcohol can shift arousal and sleep architecture directly, so any “neuroactive” claim must survive matched-comparator designs and explicit chemical assays [91,271,276].

Minimum specification follows the same stack logic. Use targeted LC–MS panels that quantify the candidate molecules and key precursors in the product and across stool, urine, and plasma, with sampling anchored to kinetics: a post-dose window for acute signals, plus steady-state profiles after repeated intake [129,271,278]. Pair exposure with proximal pathway readouts that match the hypothesized route, for example, cortisol dynamics for HPA calibration, CRP and IL-6 for inflammatory tone, barrier leakage indices, and, when feasible, CNS-adjacent markers such as glial fibrillary acidic protein (GFAP), S100B, or NF-L [129,199,278]. These markers should be interpreted as CNS-adjacent injury or glial-stress signals rather than evidence of brain delivery. Their specificity is limited, so they are most informative when interpreted together with exposure kinetics, barrier readouts, inflammatory markers, and vascular endpoints. Keep symptom endpoints domain-aligned, emphasizing sleep, fatigue, and stress reactivity over broad mood totals [272,275,278]. Small molecules are fast signals, but fermented foods also deliver larger effectors; peptides and EPS behave more like food-derived biologics and need their own logic [271,272,279].

### 4.5. Bioactive Peptides and Exopolysaccharides (EPS): “Food-Derived Biologics”

Bioactive peptides and EPS deserve their own module because they are product-defining effectors, closer to “food-derived biologics” than to generic nutrients [280,281,282]. Fermentation does not just preserve food; it rewrites protein and polysaccharide structures into sequences and topologies that encode signaling potential [65,281,283]. Two yogurts with identical macronutrients can carry different peptide spectra depending on strain proteases, fermentation time, and cold-chain drift, and those differences plausibly matter more than broad labels like “dairy” or “fermented” [65,284,285]. EPS follows the same rule: monosaccharide composition, branching, charge, and molecular weight form a structured exposure signature that can bias mucosal and immune programs [286,287,288]. The price of this mechanistic specificity is measurement [271]. If the fingerprint is unknown, the mechanism is a story, not an exposure [271,284].

Peptide fingerprints map cleanly onto vascular endpoints when framed as directionally interpretable physiology rather than as in vitro bioactivity claims [271,289,290]. The most useful bridge is renin–angiotensin–aldosterone system (RAAS) and ACE-adjacent biology: short peptides with ACE-inhibitory motifs can lower angiotensin II pressure, tilt signaling toward the ACE2–Ang(1–7) axis, and reduce the oxidative load that collapses NO bioavailability [291,292,293]. Read the causal chain like a circuit. A peptide-rich exposure should shift NO handling (higher nitrate/nitrite with lower lipid peroxidation, improved arginine–ADMA balance), then soften adhesion programs (lower VCAM-1, ICAM-1, selectins), and finally stabilize perfusion-relevant traits such as vascular reactivity and fatigue-linked exertional intolerance [65,271,290]. Redox buffering is the parallel lane: many fermented-food peptide mixtures damp TNF-α signaling to NF-κB and p38, suppress NADPH oxidase activity, and align with nuclear factor erythroid 2-related factor 2 (Nrf2)-centered antioxidant induction [65,294,295].

EPS complements peptides by acting where the tri-barrier axis is most sensitive—at the mucosal surface [286,287]. Many EPS structures resist upper-gut digestion, persist as bioaccessible polymers, and behave as low-intensity patterning signals that can shift epithelial and myeloid tone [286,287,296]. A practical, testable prediction is that tolerogenic bias is characterized by higher secretory IgA, stronger IL-22 and antimicrobial peptide programs, and fewer PRR-amplified cytokine bursts that erode junctional organization [286,287]. EPS can also act indirectly by reshaping the ecology toward SCFA-rich guilds, which support epithelial energetics and mucus output, but the mechanistic claim should remain epithelial first, microbial second [286,287,296]. Because EPS effects are structure-dependent, “amount” is not sufficient; size distribution and charge state often explain why one product calms chemokine output while another is inert [286,287,288].

Minimum measurement should therefore look like peptidomics-lite plus EPS-aware fingerprinting, backed by functional assays and comparator discipline [271,284,285]. For peptides, quantify a targeted LC–MS panel anchored to annotated sequences or motif classes, add an in vitro digestion step to estimate bioaccessibility, and pair it with simple activity assays (ACE inhibition, dipeptidyl peptidase-4 (DPP-IV), antioxidant capacity) that serve as quality control (QC), not as endpoints [280,284,294]. For EPS, report yield, molecular-weight distribution, and basic composition (neutral vs. acidic fractions), then link these to barrier and immune readouts such as TEER or FITC-dextran flux, sIgA, IL-10/IL-22, and a compact PRR-tone panel (LBP, sCD14, TNF-α, IL-6) [286,287,296]. Time-stamp exposure with early post-dose sampling plus steady-state weeks, since acute signaling and remodeling can decouple [65,271]. Confounds must be treated as design variables. Sodium alters vascular tone and sleep, and it also reroutes fermentation chemistry, so arms should be sodium-matched or sodium-measured with prespecified adjustment [271]. Biogenic amines, especially histamine and tyramine, can mimic anxiety, headache, and arousal and can move blood pressure (BP) independent of any peptide benefit; screen them alongside putrescine and cadaverine, and treat elevated batches as non-comparable exposures [271]. Match calories and protein too, because protein load can shift proteolysis and endotoxin tone [271,289].

Integration is the point: peptides and EPS should not compete with SCFAs, indoles, or bile acids; they are part of the upstream “hardware” that helps explain why those smaller molecules shift the way they do in real products [65,271,281]. A clean stack reads exposure to intermediates to integrative endpoints [271]. Fingerprints define exposure; barrier and immune tone validate containment; endothelial activation and immunothrombosis report vascular translation; clocks summarize whether repeated remodeling persists [65,234,271]. This framing also makes platform choice an engineering decision and sets up phenotype stratification, because peptide and EPS effects will be louder in participants with baseline endothelial stress or inflammatory drift [65,271,289]. Yogurt and kefir are ideal for reproducible fingerprints under controlled storage, whereas miso and tempeh are powerful only if batch chemistry, sodium, and amines are tightly controlled, and fraction-enriched variants can be produced [65,280,284]. Brazilian artisanal cheeses such as Queijo Canastra and Queijo do Marajó follow the same logic as peptide/EPS “food-derived biologics,” but with greater variance and stronger confounding pressures [271,284]. They are best used as fingerprinted, batch-qualified exemplars for proteolysis-driven exposure, with sodium and histamine/tyramine explicitly screened to avoid misattributing vascular or sleep shifts to peptides [271,284]. With modules specified as measurable exposures and interpretable intermediates, platform choice becomes an engineering decision: pick the product family that best isolates the mechanism you want to test [65,271,283].

## 5. Immunothrombosis Chapter: Platelets + Neutrophil Extracellular Trap (NETs) + Endothelial Activation

**Figure 3:**
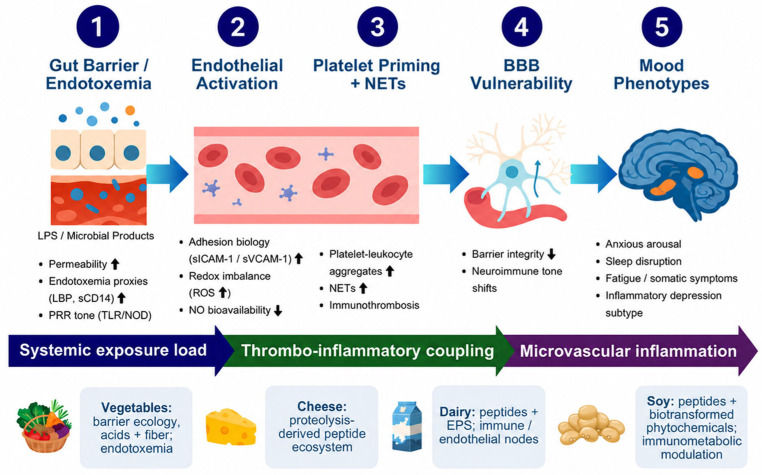
Neurovascular immunothrombosis bridge from gut barrier disruption to mood phenotypes. This figure proposes a stepwise, testable pathway linking gut-derived inflammatory exposure to depression and anxiety through neurovascular vulnerability. Increased gut permeability permits translocation of microbial products, elevating endotoxemia proxies and tuning innate immune receptors. This upstream load programs endothelial activation, expressed as adhesion biology, redox imbalance, and reduced NO bioavailability, creating a vascular substrate for thromboinflammatory coupling. Platelets become primed, platelet–leukocyte interactions intensify, and NET-associated signaling can amplify microvascular inflammation. The downstream consequence is BBB vulnerability, enabling peripheral immune signals to shape neuroimmune tone and stress-reactivity circuits that map onto symptom domains such as anxious arousal, sleep disruption, fatigue, and inflammatory depression. Product-family intervention points are indicated along the cascade: fermented vegetables plausibly act earliest by improving barrier ecology and lowering endotoxemia potential; yogurt/kefir and artisanal cheeses may act at immune and endothelial nodes via peptides and EPS; fermented soy may additionally shift immunometabolic drivers that modulate endothelial and platelet programs, especially in metabolic comorbidity phenotypes. NETs, neutrophil extracellular traps; NO, nitric oxide. Figure prepared with Canva.com.

Immunothrombosis offers a unifying vascular mechanism linking chronic stress biology and low-grade inflammation to platelet priming, NET formation, and endothelial activation, all measurable and clinically legible [297,298,299]. NETs act as DNA–protein scaffolds that trap platelets, concentrate procoagulant cues such as tissue factor, and trigger endothelial ICAM-1/VCAM-1 programs, creating a self-amplifying platelet–NET–endothelium loop [297]. This section uses that loop to motivate the relevance of depression and anxiety, specify the metabolite-to-platelet/endothelium wiring, and map food platforms and comparators that can test causality [298,299,300]. With that framing, the next subsection explains why this pathway belongs inside depression and anxiety models rather than being treated as a cardiology side note [298,301,302] (Figure 3).

### 5.1. Why Immunothrombosis Belongs in Depression/Anxiety

Depression and anxiety can be read as vascular stress tests: repeated autonomic and inflammatory surges push endothelium toward lower NO, higher adhesion signaling, and a more procoagulant surface, while platelets become easier to trigger [298,299,301]. The clinical payoff is subtle but pervasive. Long COVID and PASC frameworks show that low-grade thromboinflammation, with NET-linked coagulation signals, can reduce microvascular perfusion and cause fatigue and cognitive fog in people without overt cardiovascular disease [303,304,305]. Sleep disruption amplifies the same physiology, as obstructive sleep apnea work illustrates through nocturnal oxidative stress, endothelial dysfunction, and daytime sleepiness [298]. ME/CFS adds a complementary pattern, where persistent cytokine tone and altered cerebral blood flow track cognitive slowing [305]. This makes immunothrombosis a mechanistic bridge between subjective symptoms and objective biology, but only if the pathway is specified as platelet, NET, and endothelial programs with defined triggers [297,306].

Immunothrombosis earns its place in depression and anxiety because it is transdiagnostic: the endothelial and platelet programs that drive cardiometabolic risk also govern microvascular perfusion, barrier signaling, and leukocyte trafficking that can shape brain-relevant outcomes [298,299,301]. Cohort and meta-analytic evidence shows depression tracks impaired flow-mediated dilation (FMD) and a circulating endothelial activation signature enriched for soluble intercellular adhesion molecule 1 (sICAM-1), MCP-1, and related adhesion cues, with stronger signals in cardiometabolic comorbidity and prospective links to symptom persistence [301,302,307]. Clinical observations add reversibility, since SSRI treatment can normalize circulating endothelial injury markers and serum-induced endothelial dysfunction [308,309,310]. On the causal side, NET biology supplies a mechanistic amplifier, and LPS models show that blocking NET formation attenuates depression-like behavior alongside reduced cytokine escalation and neuroinflammation [297,298]. Because this evidence derives mainly from LPS-based preclinical models, it should be interpreted as biological plausibility rather than direct clinical proof of NET-driven depressive mechanisms in humans. This implies that mood trials can credibly test immunothrombosis when they commit to platelet and endothelial readouts, not distal events [306,311,312].

A rigorous evidentiary standard should treat immunothrombosis as a graded phenotype rather than a yes-or-no label [297,306]. The core minimum is a joint panel: platelet activation (for example CD62P, PAC-1, platelet–leukocyte aggregates or platelet factor 4 (PF4) and β-thromboglobulin), NET burden (MPO–DNA, NE–DNA, citH3, nucleosomes), and endothelial adhesion and injury signals (sICAM-1, soluble vascular cell adhesion molecule 1 (sVCAM-1), E-selectin, vWF, circulating endothelial cells), ideally summarized as a composite score since multi-marker models outperform single analytes in vascular disease [306,308,311]. In mood populations, interpretation must be stratified by baseline inflammation, adiposity, sleep apnea, and medications, because stress alone can upshift platelet reactivity and SSRIs or antiplatelet therapy can dampen trajectories [300,309,313]. With “why it belongs” established, the next step is the wiring diagram linking metabolite modules to platelet–endothelial machinery [298,299].

### 5.2. Metabolite-to-Platelet/Endothelium Wiring

Metabolite-to-platelet/endothelium wiring becomes readable when you treat it like pharmacology: which receptors are being touched, and which enzymes act as bottlenecks [314,315,316]. The clearest example is phenylacetylglutamine, a microbial metabolite that boosts platelet reactivity by signaling through platelet adrenergic GPCRs, essentially hijacking catecholamine circuitry [314,317,318]. In parallel, lipid ligands such as 12(S)-HETE can amplify thrombotic signaling via platelet GPR31, while proteases remodel receptor behavior through biased PAR1 cleavage, changing the gain on downstream Ca^2+^, p38, and phosphoinositide 3-kinase (PI3K) pathways [315,316,319]. Enzyme layers matter too because platelet activation depends on metabolic checkpoints like PKM2, hexokinase, and mechanistic target of rapamycin (mTOR)-linked programs that convert substrate availability into prothrombotic phenotype [158,316,320]. This prompts a simple logic: specify the exposure class, specify the proximal vascular targets, then test whether downstream immunothrombotic outputs move in the predicted direction [306,321,322].

Different metabolite modules can converge on the same immunothrombotic outputs through distinct entry points, and that convergence is exactly why the framework works [306,319,322]. Redox tone is a central regulatory variable: NADPH oxidase 2 (NOX2), mitochondrial ROS, and myeloperoxidase (MPO) feed the oxidative chemistry required for chromatin decondensation, so antioxidant or glutathione-centric buffering can lower NET yield without needing to name every upstream trigger [158,323,324]. In parallel, NO bioavailability shapes endothelial “stickiness”; NO donors can suppress TNF-α-driven VCAM-1 and ICAM-1 via NF-κB inhibition, while nitrosative stress shifts signaling toward inflammatory gene programs [158,320,325]. A third entry point is trafficking logic, in which NETs themselves act as feed-forward cues that amplify neutrophil ROS and IL-8 release, thereby driving further NETosis and effectively amplifying the inflammatory feed-forward loop [158,326,327]. Because convergence is the goal, the readout panel should prioritize shared endothelial and platelet outputs rather than chasing every upstream molecule in isolation [306,321,322].

Vascular target engagement is best demonstrated with time-aligned sampling of exposure panels alongside platelet activation indices, NET proxies, and endothelial activation markers, rather than assuming causality from microbiome shifts or symptom changes alone [306,315,321]. Practically, that means pre-specified kinetics for metabolomics and, where feasible, compartment-aware endothelial phenotyping since microvascular versus macrovascular programs are not interchangeable [158,321,328]. On the vascular side, a minimal panel can pair adhesion markers such as ICAM-1, VCAM-1, E-selectin, and endothelin-1 with platelet activation readouts such as soluble P-selectin or platelet-derived signals, complemented by a NET proxy layer when the hypothesis is immunothrombosis [306,321,329]. Comparator design is non-negotiable: compliance-matched vascular graft work is a reminder that “material differences” can masquerade as inflammation, and in food trials, sodium, calories, and co-metabolites can do the same [315,319,326].

### 5.3. Product Mapping

Product mapping should be hypothesis-driven, distinguishing platforms likely to deliver direct effector chemistry from those that mainly stabilize upstream ecology and barrier containment that indirectly lowers immunothrombotic tone [314,315,326]. A useful heuristic is to separate “drivers” from “enablers.” Drivers are platforms that reliably carry a defined payload, like metabolite-rich fractions or engineered delivery systems, where structure-centered design is explicitly used to overcome biological barriers and improve target engagement [314,315,326]. Enablers, in contrast, act more like governance layers in an ecosystem: they increase stability, reduce noise, and shape interactions, yet they do not themselves guarantee a strong effector signal [320,324,330]. Treating foods as modular platforms forces the same question asked in other ecosystems: what is standardized, what drifts, and where does leverage actually come from [319,326,330]. The Brazilian case studies are included to operationalize this point: they exemplify how regional products can be translated into measurable exposures and interpretable intermediates without requiring encyclopedic coverage [318,319,326]. In that sense, they are not detours, but templates for discovering and evaluating “unknown” fermented foods worldwide [318,326,330]. This framing supports a tiered testing strategy, where the first objective is to verify module engagement and the second is to assess whether immunothrombosis endpoints respond in the predicted direction and with the predicted kinetics [306,321,322].

Because fermented foods co-deliver vascularly active confounders, product studies should report sodium and biogenic amines and use matched comparators, such as live versus heat-treated or metabolite-enriched variants, with energy and salt held constant [319,326]. Sodium is not just a background nutrient in fermented matrices; it can reshape microbial ecology and, in turn, biogenic amine accumulation, as shown in controlled fermentation models where higher NaCl shifts communities and lowers histamine/tyramine-related load [319,326]. Biogenic amines then become interpretation-critical because they can perturb vascular tone, sleep, and neurovegetative symptoms in ways that mimic “benefit” or “harm” independent of the intended module [319,326]. A minimal checklist, therefore, includes per-serving NaCl, quantified histamine and tyramine, and a comparator hierarchy that perturbs only one axis at a time: culture viability, module enrichment, or amine depletion [319,326]. With this discipline, product effects can be credibly attributed to verified metabolite-module engagement and aligned to endothelial and BBB-relevant outcomes rather than to uncontrolled dietary drift [321,322,326] (Table 4).

## 6. Epigenetic Clocks + Biological Aging Endpoints

Epigenetic clocks are algorithms that estimate biological age, and age acceleration marks when that estimate runs ahead of chronological age [347]. Because clocks compress immune tone, metabolic stress, and vascular dysfunction into a single quantitative signal, they act as an integrative endpoint for linking mood symptoms to endothelial and inflammatory biology in a mechanism-first framework [352]. Here, clocks are proposed as exploratory readouts of remodeling pressure rather than as established response markers for fermented-food interventions. If future trials show verified metabolite exposure together with coordinated inflammatory and vascular changes, clock trajectories may help test whether biological aging signals move in a coherent direction across individuals [180,353]. The next subsection explains why clocks are particularly useful in depression and anxiety research, where heterogeneity makes single biomarkers easy to misread, before discussing plausible metabolite routes into methylation dynamics and then trial-ready product mapping and endpoints [353,354]. This section is explicitly hypothesis-generating. Current evidence does not establish that fermented foods remodel epigenetic clocks in depression or anxiety. Clock readouts are proposed as exploratory integrative endpoints that require longitudinal trials with verified exposure, inflammatory and vascular co-measures, and repeated intake over at least 6 to 8 weeks. At present, fermented-food randomized controlled trials with epigenetic-clock endpoints are lacking, so the proposed clock framework should be treated as a trial-design hypothesis rather than a demonstrated intervention effect.

### 6.1. Why Clocks Are Useful Here

In a mechanism-first framework, epigenetic clocks function as “remodeling time” readouts that capture whether repeated exposures actually shift organism-level set points rather than producing transient biomarker noise [347,350,355]. Longitudinal clock trajectories are therefore more informative than single cross-sectional age gaps, but they should be interpreted cautiously. In dietary or fermented-food trials, evidence for durable recalibration would require repeated sampling, verified exposure, and a realistic intervention window, preferably at least 6 to 8 weeks, together with inflammatory and vascular co-measures [350,355]. Different clocks emphasize different constructs, from chronological age approximation to mortality-linked pace measures, so a meaningful change mechanistically implies altered aging rate signals aligned with nutrient sensing, mitochondrial function, immune composition, and other hallmarks [347,356,357]. This is especially valuable in depression and anxiety because biological aging signals are not uniform across patients, so the question becomes who shows acceleration and why [40,356,358,359].

Depression and anxiety show biological aging patterns in subsets, where inflammatory tone, sleep disruption, metabolic risk, and medication context plausibly determine whether methylation trajectories drift toward acceleration or remain resilient [356,358,359,360]. Large cohorts report only modest average acceleration in major depressive disorder (MDD), yet the signal strengthens in higher-severity and childhood-adversity profiles, and in never-medicated active disease with immune shifts such as lower DNAm-inferred NK cells [356,359]. Anxiety adds its own twist: subtype and social-functioning context can align with suppressed or altered aging signals rather than uniform acceleration [352,358]. Next-generation clocks often track transdiagnostic internalizing liability more than any single syndrome, while insomnia-defined male mood-disorder groups show striking GrimAge acceleration tied to cardiometabolic proteins [357,358,360]. Once subsets are explicit, the mechanistic question sharpens to how microbial metabolites and fermented-food modules might plausibly nudge methylation control systems in those at-risk phenotypes.

### 6.2. How Microbial Metabolites Plausibly Touch Methylation Trajectories

SCFAs offer a clean mechanistic entry point because they behave like chromatin-active exposures rather than passive fuels. Butyrate and propionate can inhibit class I/II HDACs, yet they also act as acyl donors that activate p300 and write histone acylation marks, including butyrylation, propionylation, and crotonylation at open regulatory regions that control large transcriptional programs [361,362]. This architecture makes it plausible that sustained SCFA patterns could shift the transcriptional “guard rails” that stabilize methylation trajectories over time [363,364]. The most convincing intermediates are therefore proximate and time-aligned: histone acylation panels, p300 and HDAC activity readouts, chromatin accessibility, DNMT expression, and immune-cell fate shifts such as Th17 to Treg or B-cell differentiation [363,365,366]. However, chromatin plausibility is not enough; therefore, the next step is to treat inflammation reduction as a key “clock input” that can mediate durable remodeling across immune and vascular compartments [366,367,368,369]. These links remain mechanistic hypotheses for fermented foods because direct trials connecting fermented-food exposure, histone acylation, DNA methylation clocks, and depression or anxiety outcomes have not yet been established.

A lower inflammatory load is the most interpretable route to slower biological aging because cytokine tone is not just a correlate; it is a repeated stimulus that keeps hematopoiesis in “alarm mode” and pressures the epigenome toward wear-and-tear remodeling [366,367,369,370]. Large cohort data show that a latent systemic inflammation factor aligns with epigenetic age acceleration across many clocks and forecasts mortality, while geroscience syntheses place inflammaging upstream of senescence, mitochondrial stress, and immune decline [368,369,370,371]. Mechanistically, chronic IL-6, TNF-α, CRP, and related mediators can bias immune cell composition, amplify oxidative and circadian stress, and sustain pro-aging transcriptional programs [366,369,372]. To make clock shifts interpretable, trials should co-measure cytokines and acute-phase markers, immune-cell proportions, NLR-type indices, and immunosenescence panels. This makes endothelium a critical intermediate, since vascular cells both sense inflammatory traffic and express senescence-like programs that can feed back into systemic aging signatures [199,371,373,374].

Endothelial senescence-like activation programs—reflected in NO imbalance, redox stress, and adhesion signaling—provide a biologically plausible bridge from gut-derived exposures to epigenetic aging readouts because they sit where inflammation, perfusion, and barrier function collide [199,366,371,374]. Endothelial cells are continuously “sampling” circulating chemistry, so metabolite modules can plausibly inscribe aging signals through SIRT1, AMPK–mTOR, NRF2, and p53/p21 pathways that reshape permeability, vasodilatory reserve, and SASP-like cytokine output. Direct examples strengthen the logic: a gut flora–dependent metabolite such as TMAO can accelerate endothelial senescence via oxidative stress and SIRT1 repression, while broader vascular-aging syntheses highlight metabolite-sensitive control of NO and inflammatory adhesion programs [199,366,374,375]. Practically, endothelial senescence signatures and vascular function measures should be co-primary with clocks, so products are chosen for their ability to move both panels. With these mechanistic paths defined, platform selection can be made hypothesis-driven, choosing foods that reliably deliver the exposure modules needed to test clock and vascular remodeling in trials.

### 6.3. Product Mapping and Trial Endpoints

Yogurt and kefir are the most trial-friendly platforms for epigenetic clocks because they already come with a playbook for standardization: defined starter consortia, controlled fermentation kinetics, stable cold-chain storage, and high adherence when dosing is daily and food-like [105,376]. That matters if clocks are treated as “remodeling time” signals that benefit from repeated, time-resolved sampling rather than one-off snapshots [105,376]. The minimum mechanistic fingerprint should pair untargeted metabolomics with targeted SCFAs, plus optional peptide and exopolysaccharide signatures when strains are selected for bioactive release [47,105,377]. On the outcome side, clocks can be stacked with inflammatory load (hs-CRP, IL-6), endothelial aging panels (sICAM-1, sVCAM-1, vWF, selectins), and a functional readout such as FMD [62,378]. This enables comparator designs that isolate live, heat-treated, and metabolite-enriched variants while preserving energy and sodium matching, creating clean tests of clock and endothelial remodeling [62,105].

Fermented vegetables and soy ferments fit best as adjunct arms or controlled dietary backgrounds, where vegetables stabilize ecology and barrier tone, while soy can add high-information peptides and biotransformed phytochemical exposure if batch chemistry is fingerprinted and variability is constrained [47,62,379]. Pilot and crossover studies suggest that vegetable ferments shift butyrate-associated taxa and gut community structure with modest systemic inflammatory change, which is useful for “ecology support” but risky if sourcing, salt, and fermentation parameters drift [47,377]. Soy, by contrast, has clearer, biomarker-dense signals in RCTs, including lipid and metabolic changes, and even effects on cognition in older adults, yet its effects can hinge on matrix and responder status, such as equol production [93,380,381]. Both platforms, therefore, need lot-level metabolomics plus sodium and biogenic-amine reporting when relevant, and a design that fixes background intake or stratifies it explicitly [47,105]. With platform roles clarified, the section can specify a core endpoint panel that treats clocks as integrative outcomes and vascular, immune, and metabolite measures as the mechanistic scaffolding needed for causal interpretation [105,378].

## 7. Evidence Map: What Exists and What’s Missing

This section is a readiness audit: it scores psychobiotic and fermented-food evidence by how well it turns “gut–brain” claims into testable biology. The map is organized as a causality ladder, then a catalog of failure modes, and finally a platform summary, because efficacy without mechanism often reflects dosing noise, unmeasured metabolites, or uncontrolled diets rather than true pathway engagement. “Missing” evidence here means absent target or exposure verification, absent proximal biomarkers, or designs that cannot falsify the proposed mechanism. The ladder starts at the lowest rung, where endothelial and BBB models can specify targets and outputs with minimal clinical noise.

### 7.1. Causality Ladder

In vitro endothelium and BBB systems are the cleanest places to test receptor and enzyme engagement, because exposure can be clamped and proximal outputs, such as eNOS signaling, permeability, glycocalyx injury, and adhesion programs, can be read directly [382,383]. Modern BBB microvessels, organoids, and chips achieve low baseline leakage with high TEER, tight junction markers like claudin-5 and ZO-1, and functional efflux transporters, letting you quantify module effects on barrier tightening or disruption with dextran or albumin tracers [383,384,385]. They also support decisive engagement assays, including transferrin receptor or LRP1 transcytosis and chemokine-guided immune trafficking [383,384,385]. Mood inference is indirect, so a mitigation method is to pair these readouts with exposure metabolomics and then escalate [386,387]. Once a module moves these programs in the predicted direction, the next rung asks whether the same biology remains causal in living systems that include immunity, perfusion, and behavior [382,388].

Animal studies earn their rung only when they demonstrate directionality, ideally by showing that the intervention shifts platelet, NET, or endothelial programs and that blocking a defined node abolishes both the vascular phenotype and the depression or anxiety-like behavior [175,388]. The strongest designs look less like “probiotic improves behavior” and more like circuit tests: a metabolite-competent strain rescues stress phenotypes, the same strain with a knocked-out biosynthetic gene loses efficacy, and pathway blockade or rescue (for example, AhR signaling for ILA, or experimentally induced BBB opening) toggles both neuroinflammation and behavior [382,388]. Behavioral readouts still matter, but they should be bracketed by intermediates, including barrier integrity, cytokine tone, and vascular transcriptomic signatures, with timing matched to exposure kinetics [175,382]. Even strong animal causality still leaves a translational gap, so the next step is human feeding work designed to capture time-resolved exposure and proximal biology before symptom claims [386,387].

Short mechanistic feeding studies are the best human bridge because they can verify exposure chemistry, quantify immediate platelet and endothelial responses, and map kinetics in blood, stool, and urine before outcomes are diluted by weeks of life noise [386,387,389]. The strongest examples already pair intake with objective fingerprints, such as 16S plus fecal metabolomics in strain trials and metagenomics with urine and fecal metabolite panels in psychobiotic diet work, so “what was delivered” and “what moved” are both visible [387,390]. The minimum design then adds time-stamped vascular biology: platelet activation indices, endothelial adhesion and permeability markers, and aligned inflammatory and endocrine signals such as CRP, IL-6, and cortisol [175,387]. If these studies show consistent proximal movement, the field has earned the right to scale into RCTs that treat immunothrombosis and aging endpoints as quantifiable phenotypes [175,386].

RCTs become mechanistic when they preserve tight comparators, verify strain identity or processing differences, and pair symptom outcomes with an endpoint stack that includes metabolite fingerprints, endothelial activation markers, platelet or NET indices, and integrative clocks [386,387]. The most informative psychobiotic trials already hint at this architecture: multi-strain designs that show shifts in hs-CRP, cortisol awakening response, glutathione, IL-6, serotonin, stool metabolomes, or SCFA-producing taxa make it clear the intervention did something biological even when between-group symptom separation is modest [387]. Credibility then hinges on adherence verification and matched controls, including heat-killed versus live cells, matrix-matched placebos, and factorial diet designs that prevent background drift from mimicking “effects” [386,387]. The problem is that many trials skip these elements, so the next subsection names the recurring failure modes that make null results uninterpretable [386,387].

### 7.2. Why Trials Often Fail (Mechanistically)

Many psychobiotic trials collapse at the chemistry layer because, without starter verification, viability or heat-treatment confirmation, and basic metabolomics or targeted panels, the intervention is a label rather than a measurable exposure [8,391]. Reviews of RCTs repeatedly show that strains, doses, and formulations are poorly harmonized, and that “probiotics” are often treated as a generic class rather than a defined chemotype with trackable outputs [6,8]. Minimal QC should include strain ID confirmation, CFU stability across storage, documentation of inactivation when claimed, and a fingerprint that can be reproduced across batches (untargeted metabolomics or a targeted set spanning SCFAs, indoles, lactate, and key amino-acid derivatives) [391]. When these elements are missing, null results cannot distinguish biological failure from exposure failure, and positive signals do not replicate [8,391]. Even with verified products, effects can still be washed out or misattributed when confounders and participant heterogeneity are not engineered out of the design [8,391].

Mechanistic failure often reflects preventable noise, including sodium load, histamine and tyramine exposure, calorie mismatch, medication background, and uncontrolled fermented-food intake, layered onto unstratified depression and anxiety subsets with very different inflammatory and metabolic baselines [8,392,393]. Diet can remodel metabolites and the microbiota within days, while “microbiome resilience” and person-level factors can swamp short interventions, so weak controls can yield both false negatives and spurious wins [391,392]. Reviews in youth and mixed psychiatric samples repeatedly flag small cohorts, variable baseline severity, and heterogeneous products as the recipe for nulls that teach nothing, while pharmacomicrobiomics data add a further confound because psychotropics can shift the microbiome and response propensity [8,392,393]. The fix is boring but decisive: sodium and amine reporting, calorie matching, lead-in stabilization, adherence biomarkers, and stratification by sleep, adiposity, inflammation, and medication [8,392]. These constraints set up a final, practical question: given the evidence and failure modes, which product families are best positioned for mechanism-first trials, and which should be positioned as adjuncts [392,394].

Clinically, the most defensible role for fermented foods at present is as adjunctive, mechanism-aligned interventions, especially in patients with inflammatory, metabolic, or neurovascular features [392,394]. Translation should therefore move away from generic advice to “eat more fermented foods” and toward matrix-specific selection based on biochemical profile, tolerability, and measurable target engagement [392,394,395,396,397]. Within that framework, fermented vegetables are most naturally aligned with barrier reinforcement and endotoxemia reduction, dairy with standardized peptide/EPS delivery and endothelial-redox hypotheses, and soy with peptide-rich immunometabolic and bile-acid-linked mechanisms [394,398,399]. Practical implementation should be framed in terms of dose, duration, timing, and biochemical tolerability rather than solely on food identity [394]. Moderate daily exposures sustained over several weeks are more plausible than acute dosing for ecological and metabolic adaptation, but sodium load, biogenic amines, gastrointestinal sensitivity, and sleep-related tolerability must be considered, as they can alter vascular tone and symptom perception [394,395,396,397,399].

Fermented foods should not be positioned as substitutes for pharmacological or psychotherapeutic treatment [8,392]. Their more plausible role is as systems-level adjuncts capable of modulating upstream drivers such as inflammation, barrier dysfunction, and metabolic dysregulation, thereby potentially improving resilience and treatment responsiveness within a precision nutritional psychiatry framework [8,28,275,392,394,396,397,400,401,402]. Long-term effects remain poorly characterized [8,392,394]. Chronic intake may induce more sustained shifts in microbiota function and metabolite production, but adaptive microbial and host responses could also attenuate effects over time, making the durability of benefit an open mechanistic and clinical question [392,394,403]. Longitudinal studies are therefore needed to determine whether continuous intake, cycling, or diversification of fermented foods best sustains benefit, and whether adherence, cultural acceptability, and safety constrain real-world implementation in specific patient groups [392,394]. Fermented foods should also be contextualized within broader dietary and therapeutic strategies [8,392,394]. Mediterranean-style and other anti-inflammatory dietary patterns likely provide a permissive background for fermented-food effects, whereas fiber supplementation may reproduce only part of the mechanism by increasing SCFA availability without the added peptide, EPS, and fermentation-derived chemical complexity [394,404,405,406]. Compared with probiotic capsules, fermented foods offer a more ecologically valid but less standardized exposure, and this trade-off becomes even more complex when concurrent antidepressant or other medications alter microbiome composition and metabolism [392,393].

Even with rapid expansion of mechanistic insight, the clinical evidence base remains heterogeneous and at times inconclusive [8,392,407]. That mismatch between mechanistic richness and trial interpretability explains why product-family readiness must be judged not only by biological plausibility but also by exposure control, target engagement, and phenotype alignment [391,392]. One network meta-analysis found that probiotics were superior to placebo and several antidepressant comparators in adults with major depressive disorder, whereas a monotherapy-focused meta-analysis in unmedicated depression showed only a small benefit that became non-significant after sensitivity analyses [408,409]. Together, these findings suggest that probiotics are promising and well tolerated, but should be tested in larger, independent, phenotype-stratified trials, especially in mild-to-moderate or inflammation-linked depression.

Yet heterogeneity across strains, doses, matrices, and trial design sharply limits interpretation, reinforcing the need to treat positive and null findings as information about exposure fidelity rather than as simple verdicts on “probiotics” or “fermented foods” [6,8,391]. Whole fermented foods remain underrepresented in controlled trials relative to capsule-based probiotics, creating a persistent translational gap between real dietary exposures and mechanism-first hypotheses [392,394]. Closing that gap will require human studies that define the food matrix as carefully as the clinical phenotype [392,394,410,411,412]. A central limitation is that many studies still do not verify whether candidate metabolite modules—such as SCFAs, indoles, bile acids, peptides, or EPS-linked signatures—were actually engaged [391,394]. Without exposure verification, symptom outcomes alone provide only weak causal inference, especially when products differ in chemistry, viability, sodium content, and storage history [8,391]. The following product-family summary therefore applies this failure-mode logic to matrix readiness rather than restating the full checklist for sodium, amines, diet, adherence, and stratification.

### 7.3. Product-Family Summary of Evidence Readiness

Fermented vegetables are increasingly plausible as ecological stabilizers, nudging taxa, organic acids, and, occasionally, GABA in ways that may support barrier tone, yet their readiness is constrained by batch-to-batch microbiology, salt variability, and the ever-present risk of biogenic amines or other process byproducts [413,414,415]. Fermented soy is biologically denser: isoflavone biotransformation, peptide release, and product-specific neuroactive signatures can generate high-information signals, but these effects ride on cultural dietary baselines, equol-responder status, and process-driven divergence even within a single named product family [62,63,416]. Their readiness therefore depends on applying Section 7.2 failure-mode controls to each matrix, with emphasis on batch identity, exposure verification, and phenotype alignment rather than repeating the full trial-design checklist here [47,414,417]. This contrast explains why dairy often becomes the anchor platform, since it best supports standardized dosing, clean comparators, and dense mechanistic sampling [47,105].

Yogurt and kefir are the most evidence-ready platforms because dairy fermentation is unusually controllable: starters can be specified, viable counts and storage can be standardized, and dosing can be repeated with high adherence and tolerability [47,105]. This feasibility provides causal leverage because tight matrix control allows a defined strain–metabolite fingerprint to be linked to aligned biological changes. Downstream changes in aging markers or symptom trajectories can then be interpreted within the same cohort [47,214,377]. In other words, dairy turns “psychobiotics” from a brand label into a measurable exposure with interpretable biology [47,377]. With the evidence ladder, failure modes, and platform readiness clarified, the next section can move from mapping to execution by specifying the minimal design template for mechanism-first psychobiotic trials [47,377].

## 8. Translation Playbook: Next-Generation Study Designs

A translation playbook is needed because the psychobiotics literature still too often trades on promise without pinning down what actually moves in the body. Across reviews and proposed pipelines, the same bottlenecks recur: heterogeneous products, weak strain or matrix attribution, and mechanistic readouts that are missing or incomparable, especially for metabolites whose quantification is technically demanding and timing-sensitive. The playbook solves this by standardizing what counts as evidence, then operationalizing it into panels, comparator templates, and phenotype stratification that can survive replication and regulation [418,419]. The starting point is a minimal mechanistic panel that treats exposure as measurable chemistry and biology as a linked stack rather than isolated endpoints [354,418,419] (Table 5).

### 8.1. Minimum Mechanistic Panel (For Publishable Causality)

A “minimum” mechanistic panel should be publishable because it is vertically integrated, not exhaustive: it verifies exposure modules first (strain identity plus a metabolite fingerprint such as SCFAs and Trp/indole derivatives), then reads the next dominoes in tissues that transmit gut signals [439]. Barrier integrity (permeability markers and tight-junction-linked readouts) determines whether luminal chemistry can plausibly reach the circulation [440]. Endothelial activation and platelet or NET indices then translate immune traffic into a vascular phenotype with clear directionality [441]. Finally, integrative endpoints like immunothrombosis composites and epigenetic aging pace summarize accumulated wear [442]. That chain begins with exposure verification, because without a quantified metabolite and strain signature, downstream biology cannot be attributed [443].

Metabolite modules serve as the anchor because they turn food interventions into measurable dosing: you fingerprint the product at baseline, then track time-resolved signatures in stool, urine, and blood to confirm delivery, absorption, and host handling, rather than assuming “one serving” is a unit of exposure [439]. Minimal coverage can stay lean but must be interpretable, typically SCFAs, Trp, and indole derivatives, key biogenic amines, and a compact bile acid or phenolic panel, read with attention to kinetics and compartment logic [444]. In this framing, “dose” is the achieved concentration time-course plus its reproducibility across batches and participants [445]. Once exposure is real, the next question is whether it shifts the gut barrier and immune traffic that often mediate systemic vascular and mood-relevant biology [446].

The barrier module should quantify permeability and mucosal immune tone because small changes in microbial translocation can amplify cytokine signaling, nudge coagulation tone, and prime vascular activation, creating downstream noise that looks like mood biology but is really immune traffic [447]. Minimum outputs should separate “pore” versus “leak” behavior, then pair these with translocation proxies and a compact cytokine set that includes both drivers and brakes, so you can tell barrier opening from generic inflammation [448]. Add one epithelial repair or stress marker to distinguish injury from remodeling [449]. If barrier readouts move in the predicted direction, endothelial phenotypes become the next mechanistic checkpoint linking circulating exposures to perfusion, adhesion programs, and aging signatures [449].

Endothelial readouts should capture the balance between NO signaling, redox stress, and adhesion activation, since this triad translates inflammatory load into perfusion and barrier-function consequences that can propagate into whole-body aging and brain vulnerability [450,451]. A minimal panel can stay lean: one vasodilatory axis marker, plus a redox pair that reflects oxidative burden and antioxidant capacity; then an activation set anchored on VCAM-1 or ICAM-1, with an endothelial microparticle or glycocalyx-injury proxy [450,452]. Directionality should be explicit: higher NO bioavailability with lower oxidative markers and reduced adhesion signals indicates de-activation, while the opposite pattern flags senescence-like programming and SASP drift [371,453]. Because endothelial activation often travels with immunothrombosis, the panel should next quantify platelet and NET-linked programs that operationalize vascular risk biology [450,454].

Platelet activation markers and NET-related indices provide a high-signal readout of immunothrombosis that is mechanistically close to endothelial stress, sensitive to inflammatory tone, and feasible to measure repeatedly in short and long trials [20,455]. This module matters because platelets can trigger NETosis and then get trapped in NET scaffolds, creating a feed-forward thromboinflammatory loop that is targetable, not just correlative [20,456]. Minimum feasible markers can stay pragmatic: soluble P-selectin or platelet–monocyte aggregates for platelet activation, PF4 as a platelet release signal, and a NET core such as MPO–DNA or citrullinated histone H3, optionally paired with vWF and ADAMTS13 as a functional hinge [338]. With exposure, barrier, vascular activation, and immunothrombosis quantified, epigenetic clocks can be interpreted as an integrative “pace” endpoint rather than a decorative add-on.

Clocks should be treated as longitudinal pace indicators that summarize whether repeated exposures and intermediate pathway shifts actually remodel system-level set points, with interpretation anchored to concurrent changes in inflammation, endothelial state, and immune cell composition. A meaningful shift is not “age reversal” on paper, but a reproducible change in a morbidity-linked pace measure that tracks in the same direction as improved barrier integrity, lower cytokine tone, and quieter vascular activation [371,455]. Clock selection should be purpose-built: pair one mortality or phenotypic clock with a process-leaning option, and repeat both to separate signal from cell-mixture drift. These clock measures should be interpreted as complementary longitudinal indicators rather than definitive evidence of biological age reversal. Once the minimal panel is fixed, trial design becomes a question of product-centered comparators that isolate live microbes, postbiotic chemistry, and matrix effects.

### 8.2. Three Trial Templates (Product-Centered)

A yogurt or kefir template works best as a three-arm design: live fermented product, pasteurized after fermentation, and a chemically acidified dairy control matched for calories and pH [457,458]. Adherence is tightened with portioned servings and container returns [457,458]. Verification must be dual: strain tracking in stool to test passage or engraftment, plus LC–MS fingerprints of peptides, acids, and microbial metabolites in product and urine or plasma as the dose readout [457,459]. Endpoints map to the causal question: if only live shifts barrier, endothelial, or immunothrombosis modules, colonization matters; if pasteurized matches it, postbiotic chemistry is sufficient [457]. If dairy yields a low-noise signal, plant ferments can follow with sodium, amine, and batch controls [459]. A low-expansion extension is to treat selected artisanal cheeses (e.g., Canastra, Marajó) as a peptidome-forward dairy subcase, only when matched for sodium and batch-certified for biogenic amines [459,460]. This keeps the dairy logic intact while letting peptide-rich matrices be evaluated without inflating the design [457,459].

A kimchi or sauerkraut template should be engineered like a dosing study, not a cultural food swap: fixed calories, fixed fiber, and either low amount of sodium or deliberately clamped to a narrow target so salt-driven hemodynamics cannot masquerade as “psychobiotic” biology [461]. Each batch needs release criteria, including starter-consortium identity, viable counts, and a metabolomic fingerprint that explicitly quantifies lactate, acetate, GABA, and biogenic amines such as histamine and tyramine, then tracks their kinetics in stool, urine, and blood through standardized storage and shelf-life [459,461]. Mechanistic “success” is directional, not poetic: reduced permeability and inflammatory traffic, plus quieter endothelial activation, at unchanged sodium exposure [457,462]. For soy ferments, the handle shifts toward peptide and phytochemical transformation, so fractionation and immunometabolic readouts become central [457,461]. Cassava ferments can serve as a complementary plant-ferment template, with the dominant knob being starch remodeling and acidification, not sodium-driven hemodynamics [461]. Tucupi and puba/carimã are especially useful when the goal is to link defined organic-acid exposure and substrate steering to permeability and PRR-tone readouts under standardized storage and batch chemistry [461,462].

### 8.3. Stratify by Phenotype

**Figure 4:**
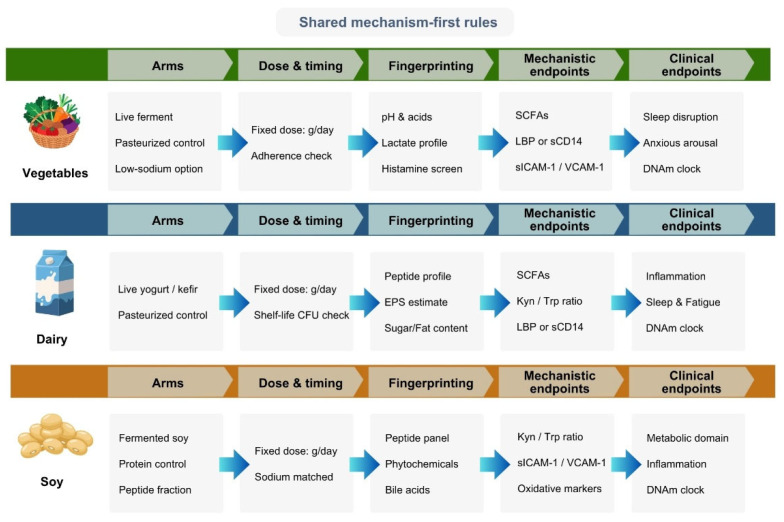
Trial roadmap for mechanism-first fermented-food psychobiotics in depression and anxiety. This roadmap summarizes three pragmatic trial templates aligned with distinct fermented-food platforms while adhering to a shared mechanism-first logic. Each template specifies core arms (live versus pasteurized or matched-matrix controls), fixed dosing in g/day with standardized timing, and batch-level fingerprinting to document what was actually delivered (pH and titratable acidity for vegetables, peptide and EPS signatures for yogurt/kefir, and peptide plus biotransformed metabolite modules for fermented soy). Mechanistic endpoints are harmonized across platforms to enable cross-study comparability, including SCFA and Trp-KYN modules, barrier and endotoxemia markers (LBP, sCD14), endothelial activation (sICAM-1, sVCAM-1), and an optional platelet panel when immunothrombosis is a claim. Clinical endpoints prioritize symptom-domain readouts (sleep disruption, anxious arousal, fatigue, and inflammatory depression features) rather than total scores alone. Epigenetic clocks are integrated as a systems-level layer, comprising at least one DNAm clock and one pace-of-aging metric, and are best suited to interventions lasting 6–8 weeks or longer. DNAm, DNA methylation; EPS, exopolysaccharides; KYN, kynurenine; LBP, lipopolysaccharide-binding protein; SCFA, short-chain fatty acid; sCD14, soluble cluster of differentiation 14; sICAM-1, soluble intercellular adhesion molecule 1; sVCAM-1, soluble vascular cell adhesion molecule 1; Trp, tryptophan. Figure prepared with Canva.com.

Phenotype stratification is the antidote to “average-effect” ambiguity. Pre-specify four high-yield strata: inflammatory depression, anxious arousal, metabolic-syndrome comorbidity, and a sleep-disturbed subtype, because each plausibly locks in baseline cytokine tone, endothelial stress, platelet reactivity, and clock pace that governs whether a psychobiotic can move the mechanistic stack. Use PATH-style principles of predictive heterogeneity by defining strata based on multivariable risk and effect models, then report absolute effects within strata rather than fishing for one-variable subgroups [37]. This turns null results into interpretable evidence and often improves power through prognostic enrichment [463]. With strata pre-specified and the mechanistic panel fixed, the next step is to formalize a minimal design template that researchers can reuse across products and populations [354]. This is especially important because inflammatory and non-inflammatory depressive phenotypes, as well as cardiometabolic comorbidity states, are unlikely to respond equally to barrier-, peptide-, or bile-acid-dominant interventions. Baseline microbial diversity and metabolic comorbidity should also be considered response modifiers, because identical fermented-food exposures may yield very different metabolite outputs across obesity, insulin resistance, hypertension, and low-diversity microbiome states [464] (Figure 4).

### 8.4. Limitations of This Review and Mechanistic Inference

This review is narrative and conceptual. It does not provide a formal risk-of-bias assessment or quantitative synthesis. Because no systematic search protocol, screening flow, or preregistered eligibility criteria were used, the evidence base should be interpreted as conceptually selected rather than exhaustive. Several proposed pathways are supported by indirect evidence from cellular systems, animal studies, metabolomics, vascular biology, or probiotic trials rather than by fermented-food RCTs in depression and anxiety. Therefore, the framework should be read as a testable roadmap for future studies, not as proof that specific fermented foods improve mood through defined vascular, BBB, immunothrombotic, or epigenetic mechanisms.

## 9. Conclusions

This review presents a mechanism-first framework for evaluating fermented foods in depression and anxiety as defined exposures rather than broad dietary labels. Its main contribution is methodological. Products should be characterized at the batch level, exposure should be verified with metabolite and safety panels, target engagement should be measured at barrier, endothelial, immunothrombotic, and BBB-relevant interfaces, and outcomes should be interpreted by phenotype and symptom domain. The framework does not establish that specific fermented foods improve mood through these pathways. Instead, it provides reusable trial templates, minimal biomarker panels, comparator rules, and falsifiable hypotheses for future studies. Priority should now be placed on standardized products, sodium and biogenic-amine control, compartment-aware kinetics, prespecified stratification, and longitudinal designs that can distinguish mechanistic target engagement from symptom-only association [418,419] (Table 6).

## Figures and Tables

**Table 1:** Mechanism-first standardization framework for fermented-food psychobiotic platforms. The table maps fermented vegetables, dairy, soy, Brazilian cassava ferments, and artisanal cheeses to mechanistic levers, key confounds, biomarker endpoints, and minimum reporting parameters required for interpretable depression/anxiety trials.

Product Platform (Examples)	Delivered Mechanistic Levers	Key Confounds to Report/Control	Best Mechanism-First Endpoints	Minimum Standardization Checklist	Ref.
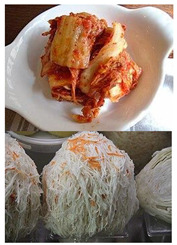 Live fermented vegetables (kimchi, sauerkraut)	Organic acids, live LAB ecology, and fiber steering; tests gut-barrier and endotoxemia-linked endothelial hypotheses.	Sodium; histamine and tyramine; recipe ingredients; storage time and temperature; batch acid profile.	LBP, sCD14, permeability markers, cytokines, sICAM-1, sVCAM-1; sleep disruption and anxious arousal.	Salt percentage; fermentation time/temp; pH and titratable acidity; lactate/acetate profile; amine screen; live or pasteurized status; dose (g/day).	[88,89,90,91,92]
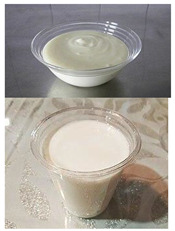 Fermented dairy, trial friendly (yogurt, kefir)	Defined cultures, lactate, peptides, and EPS; tests immune and barrier signaling with feasible comparators.	Added sugar; fat percentage; lactose intolerance; culture definition; pasteurization status; storage time.	Peptide/EPS profile; inflammatory markers; endothelial function proxies; stratified depression/anxiety phenotypes.	Culture or strain identity; CFU at end of shelf life; live or pasteurized comparator; peptide fingerprint; EPS estimate if feasible; sugar/fat content; dose and timing.	[93,94,95,96,97]
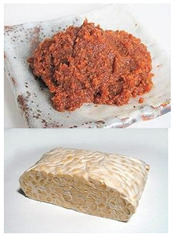 Fermented soy, mechanistic diversity (miso, tempeh)	Proteolysis and microbial biotransformation generate peptides and soy metabolites; tests immunometabolic and vascular-redox hypotheses.	Sodium; heating or cooking effects; portion size; background diet; biogenic amines.	Oxidative stress and NO-related markers; bile-acid or FXR/TGR5 readouts; fatigue and somatic symptoms.	Product form; sodium per serving; preparation method; peptide or amino-acid profile; fermentation duration; microbial label if available; dose (g/day).	[91,92,93,94,98]
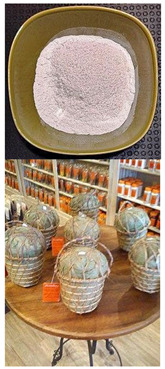 Brazilian cassava ferments, case study (tucupi; puba/carimã; polvilho azedo; farinha d’água)	Acidified carbohydrate matrix and fermentation-altered starch; case-study platform for substrate steering and barrier-to-endothelium hypotheses.	Processing and storage variability; spontaneous fermentation; biogenic amines; sodium if used; acid-profile variability; tucupi processing consistency.	SCFA module; metabolomics; permeability or endotoxemia markers; endothelial activation.	Product definition; fermentation time/temp; pH and titratable acidity; lactate/acetate profile; starch properties if feasible; amine and sodium screen; dose (g/day).	[90,99,100]
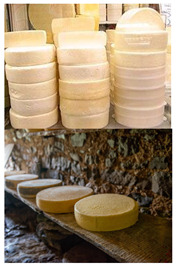 Brazilian artisanal cheeses, case study (Queijo Canastra; Queijo do Marajó)	Proteolysis and endogenous starter ecology generate peptide and EPS signatures; case-study comparator for artisanal dairy matrices.	Raw milk status; salt content; maturation time and conditions; batch microbiota; biogenic amines; safety constraints.	Peptide/EPS module; redox/NO and inflammation markers; endothelial function proxies.	Milk type and pasteurization; salt percentage; maturation time/temp; peptide fingerprint; microbial profile if feasible; amine screen; dose (g/day).	[92,94,101,102]

Image credit (Wikimedia Commons): Kimchi, Baptori, Paris 20 September 2016, photo by Guilhem Vellut, Wikimedia Commons, https://commons.wikimedia.org/wiki/File:Kimchi,_Baptori,_Paris_20_September_2016.jpg (accessed on 27 June 2026), licensed under Creative Commons Attribution 2.0 Generic (CC BY 2.0), https://creativecommons.org/licenses/by/2.0 (accessed on 27 June 2026). (Wikimedia Commons). Fresh Sauerkraut in the market Danilovsky Market, Moscow, Russia (25840010287).jpg, photo by Andrey Filippov 安德烈, Wikimedia Commons, https://commons.wikimedia.org/wiki/File:Fresh_Sauerkraut_in_the_market_Danilovsky_Market,_Moscow,_Russia_(25840010287).jpg (accessed on 27 June 2026), licensed under Creative Commons Attribution 2.0 Generic (CC BY 2.0), https://creativecommons.org/licenses/by/2.0 (accessed on 27 June 2026). Yogurt, Samoa, Yoggi original.JPG, photo by Jin Zan, Wikimedia Commons, https://commons.wikimedia.org/wiki/File:Yogurt,_Samoa,_Yoggi_original.JPG (accessed on 27 June 2026), licensed under Creative Commons Attribution ShareAlike 4.0 International (CC BY-SA 4.0), https://creativecommons.org/licenses/by-sa/4.0 (accessed on 27 June 2026). Kefir 1.jpg, photo by Scudsvlad, Wikimedia Commons, https://commons.wikimedia.org/wiki/File:Kefir_1.jpg (accessed on 27 June 2026), licensed under Creative Commons Attribution ShareAlike 4.0 International (CC BY-SA 4.0), https://creativecommons.org/licenses/by-sa/4.0 (accessed on 27 June 2026). Miso 003.jpg, Red Miso, by oofree.net, Wikimedia Commons, https://commons.wikimedia.org/wiki/File:Miso_003.jpg (accessed on 27 June 2026), licensed under Creative Commons CC0 1.0 Universal Public Domain Dedication, https://creativecommons.org/publicdomain/zero/1.0/ (accessed on 27 June 2026). Tempeh raw.jpg, photo by Christian Franke, Wikimedia Commons, https://commons.wikimedia.org/wiki/File:Tempeh_raw.jpg (accessed on 27 June 2026), licensed under Creative Commons Attribution ShareAlike 3.0 Unported (CC BY SA 3.0), https://creativecommons.org/licenses/by-sa/3.0 (accessed on 27 June 2026). (commons.wikimedia.org). Polvilho azedo.jpg, photo by Lichinga, Wikimedia Commons, https://commons.wikimedia.org/wiki/File:Polvilho_azedo.jpg (accessed on 27 June 2026), licensed under Creative Commons Attribution ShareAlike 4.0 International (CC BY-SA 4.0), https://creativecommons.org/licenses/by-sa/4.0/ (accessed on 27 June 2026). Cropped and resized for layout; changes indicated; license terms retained. Farinha de mandioca d’agua paraense (6892622493).jpg, photo by Monica Kaneko, Wikimedia Commons, https://commons.wikimedia.org/wiki/File:Farinha_de_mandioca_d%27agua_paraense_(6892622493).jpg (accessed on 27 June 2026), licensed under Creative Commons Attribution ShareAlike 2.0 Generic (CC BY-SA 2.0), https://creativecommons.org/licenses/by-sa/2.0/ (accessed on 27 June 2026). (commons.wikimedia.org). Queijo canastra, Minas Gerais.jpg, photo by Marcelo Costa, Wikimedia Commons, https://commons.wikimedia.org/wiki/File:Queijo_canastra,_Minas_Gerais.jpg (accessed on 27 June 2026), licensed under Creative Commons Attribution 2.0 Generic (CC BY 2.0), https://creativecommons.org/licenses/by/2.0 (accessed on 27 June 2026). Photo licensed from Vecteezy under the Free License. Copyright remains with the contributor. Source: Vecteezy.com. https://www.vecteezy.com/free-photos/cellar (accessed on 27 June 2026). Cropped and resized for layout; no substantive content changed. CFU, colony-forming units; EPS, exopolysaccharides; FXR, farnesoid X receptor; g/day, grams per day; LAB, lactic acid bacteria; LBP, lipopolysaccharide-binding protein; NO, nitric oxide; pH, potential of hydrogen; SCFA, short-chain fatty acid; sCD14, soluble cluster of differentiation 14; sICAM-1, soluble intercellular adhesion molecule-1; sVCAM-1, soluble vascular cell adhesion molecule-1; TGR5, Takeda G protein-coupled receptor 5.

**Table 2:** Safety and confounder control in fermented-food psychobiotic trials. This table summarizes key fermented-food exposures that may confound depression and anxiety trials, specifying reporting or measurement needs, minimal control actions, high-risk matrices, and medication-relevant cautions, including MAOIs, antihypertensives, immunosuppressants, disulfiram, and sedatives.

Item	Mechanistic Risk	Report/Measure	Control Action	Medication Notes	High-Risk Platforms	Ref.
Sodium	BP, vascular tone, endothelial-marker, and fatigue confounding.	mg/serving and mg/day; salt percentage if feasible; prespecified sodium cap or comparator-matching target.	Match sodium across arms or cap intake; stabilize background salt.	Kimchi/sauerkraut; miso; aged cheeses; cassava when salted.	BP, vascular tone, endothelial-marker, and fatigue confounding.	[110,111,112,113,114]
Histamine, tyramine	Arousal, headache, flushing, and vascular reactivity; levels rise with aging or storage.	Histamine and tyramine in mg/kg product or mg/serving; fermentation or maturation time; storage temperature; batch-release threshold.	Set matrix-specific thresholds; fix fermentation window; maintain strict cold chain.	Aged cheeses; kimchi/sauerkraut; some miso/tempeh; spontaneous cassava.	Arousal, headache, flushing, and vascular reactivity; levels rise with aging or storage.	[91,107,109,115]
Alcohol traces	Sleep, anxiety, platelet, and vascular-tone confounding.	Ethanol percentage *v*/*v* when plausible; dosing time relative to sleep.	Exclude or cap ethanol; avoid evening dosing if sleep endpoints are used.	Kefir; spontaneous ferments; niche artisanal products.	Sleep, anxiety, platelet, and vascular-tone confounding.	[116,117,118,119,120]
Additives/sweeteners	Glycemia, inflammation, permeability, and GI-symptom confounding.	Added sugar, kcal/serving, additive list, and sweetener type.	Prefer minimal-additive products; energy-match and sugar-match comparators.	Flavored yogurt/kefir; processed products across platforms.	Glycemia, inflammation, permeability, and GI-symptom confounding.	[121,122,123,124,125]
Live vs. pasteurized	Live ecology versus postbiotic chemistry and safety distinction.	Live or pasteurized status; CFU at end of shelf life for live arms.	Predefine live or pasteurized design; verify CFU or no-growth by batch.	Yogurt/kefir; vegetable ferments; raw-milk cheeses.	Live ecology versus postbiotic chemistry and safety distinction.	[90,126,127,128]
Batch variability	Drift in acids, peptides, EPS, and amines can change target engagement.	Batch ID; pH and titratable acidity; targeted acid, amine, peptide, or EPS fingerprint.	Lock batches; apply QC release criteria; retain samples; report AEs by batch.	Artisanal cheeses; spontaneous cassava; home-style vegetables; less standardized dairy.	Drift in acids, peptides, EPS, and amines can change target engagement.	[8,91,109,129,130]
Dose + timing	Exposure kinetics and dosing time can confound sleep or anxious arousal.	g/day; dosing time; adherence; fed or fasting state.	Fix dose and timing; align with blood draws and sleep measures; titrate for GI tolerance.	All platforms.	Exposure kinetics and dosing time can confound sleep or anxious arousal.	[131,132,133,134]
Storage/shelf-life	Viability and amines shift with time and temperature, changing exposure direction.	Storage temperature; days since production; pH drift; CFU for live products.	Define dosing window; enforce cold chain; avoid end-of-life products.	Vegetables; kefir; artisanal cheeses; spontaneous cassava.	Viability and amines shift with time and temperature, changing exposure direction.	[89,130,135,136,137]

AE, adverse event; BP, blood pressure; CFU, colony-forming units; EPS, exopolysaccharides; g/day, grams per day; GI, gastrointestinal; kcal, kilocalories; MAOI, monoamine oxidase inhibitor; mg/day, milligrams per day; mg/serving, milligrams per serving; pH, potential of hydrogen; QC, quality control; *v*/*v*, volume/volume.

**Table 3:** Metabolite and effector classes are mapped from proximal molecular targets to endothelial, immunothrombotic, BBB, and neuroimmune readouts linked to depression and anxiety domains. Product-family assignments reflect matrix chemistry and fermentation biology, supporting endpoint selection, confound control, and phenotype stratification.

Metabolite/Effector Class	Primary Targets (Examples)	Endothelium/Immunothrombosis Readouts	BBB/Neuroimmune Readouts	Depression/Anxiety Domains Most Linked	Product Families Most Plausible to Drive It	Ref.
SCFAs (butyrate/propionate/acetate)	GPCR signaling; HDAC modulation	sICAM-1/sVCAM-1; endothelial ROS; NO bioavailability proxies; platelet activation panels (secondary)	BBB permeability markers; microglial priming signatures (preclinical); cytokine shifts	Inflammatory depression; sleep disturbance; stress reactivity	Vegetables + sourdough background; yogurt/kefir as ecology modulator; soy as supportive matrix	[27,218,219,220,221]
Indole derivatives (Trp axis)	AhR signaling; immune tolerance programs	endothelial activation markers; permeability assays (in vitro)	BBB integrity markers; KYN/Trp ratio; immune transcriptomics	Cognitive-affective symptoms; inflammatory depression subtype	Vegetables; soy; dairy indirectly	[222,223,224,225,226]
Bile acids (secondary BA signaling)	FXR/TGR5 immunometabolic signaling	endothelial inflammation markers; metabolic inflammation (insulin resistance proxies)	neuroinflammatory tone; BBB markers (context)	Depression/anxiety with metabolic comorbidity; fatigue	Dairy and soy strongest; vegetables as supportive ecology	[227,228,229,230,231]
Bioactive peptides	RAAS/ACE-related signaling; redox/NO pathways	endothelial function (FMD in humans); NO proxies; oxidative stress	indirect BBB effects via endothelium; inflammatory cytokine reduction	Somatic symptoms; fatigue; vascular-risk co-travel	Yogurt/kefir and soy (tempeh/miso)	[232,233,234,235]
EPS/microbial structural components (postbiotic-like)	PRR tuning (TLR/NOD); barrier reinforcement programs	adhesion molecules; glycocalyx shedding markers (e.g., syndecan-1)	neuroimmune tone (systemic-to-central)	Anxiety with inflammatory signatures; stress reactivity	Kefir/yogurt; vegetables (strain-dependent)	[42,105,236,237,238]

ACE, angiotensin-converting enzyme; AhR, aryl hydrocarbon receptor; BA, bile acids; BBB, blood–brain barrier; EPS, exopolysaccharides; FXR, farnesoid X receptor; FMD, flow-mediated dilation; GPCR, G protein-coupled receptor; HDAC, histone deacetylase; KYN, kynurenine; NO, nitric oxide; NOD, nucleotide-binding oligomerization domain receptor; PRR, pattern-recognition receptor; RAAS, renin–angiotensin–aldosterone system; ROS, reactive oxygen species; SCFAs, short-chain fatty acids; sICAM-1, soluble intercellular adhesion molecule-1; sVCAM-1, soluble vascular cell adhesion molecule-1; TGR5, Takeda G protein-coupled receptor 5; TLR, Toll-like receptor; Trp, tryptophan.

**Table 4:** A practical modular panel linking fermented-food exposures to pathway nodes and 1–2 tractable target-engagement biomarkers across SCFA, barrier/endotoxemia, endothelial, immunothrombotic, tryptophan, and epigenetic-aging modules, with product-platform examples and mandatory sodium/biogenic-amine confound control to strengthen inference. Readouts in this table are intended to support target-engagement inference only when exposure, compartment, timing, and confound control are aligned. To reduce overinterpretation, each module should be assigned an evidence status, such as established human association, preclinical mechanism, indirect fermented-food evidence, or conceptual hypothesis. This evidence label should accompany mechanistic claims in the text and tables.

Module (Exposure)	Key Targets/Nodes	Minimal Target-Engagement Readouts (Pick 1–2)	Most Sensitive Domains	Product Platforms (Examples)	Ref.
SCFA axis (butyrate, propionate, acetate)	FFAR2/FFAR3 (GPR43/GPR41); HCAR2 (GPR109A); HDAC modulation (butyrate)	Plasma or fecal SCFAs (targeted); IL-6 or hsCRP	Inflammatory depression; sleep disturbance; stress reactivity	Kimchi/sauerkraut; sourdough whole grains; yogurt/kefir; Brazil: cassava ferments (puba/carimã, polvilho azedo, farinha d’água, tucupi)	[221,331,332,333,334]
Barrier/endotoxemia (upstream driver)	PRR tone (TLR/NOD); tight-junction programs (indirect)	LBP or sCD14; optional fecal calprotectin (GI-prominent)	Inflammatory depression; anxiety with GI symptoms	All platforms, especially live ferments; Brazil: cassava ferments	[6,150,168,335]
Endothelial activation (remodeling link)	NF-κB programs; adhesion biology	sICAM-1 or sVCAM-1; optional E-selectin	Vascular-risk depression/anxiety; fatigue and somatic symptoms	* All platforms; Brazil: Canastra/Marajó cheeses (peptide-forward), plus cassava ferments (barrier-forward)	[301,336,337]
Immunothrombosis (platelet activation)	Platelet activation; thrombo-inflammatory coupling	Platelet P-selectin (flow) or aggregation (single-agonist panel)	Anxious arousal/stress reactivity in inflammatory phenotypes; vascular-risk bridge	All platforms (indirect); include only when claiming immunothrombosis relevance	[315,338,339,340,341]
Tryptophan diversion (neuroimmune bridge)	IDO/TDO induction; kynurenine shift under inflammation	KYN/tryptophan ratio (targeted)	Inflammatory depression; cognitive-affective symptoms	Cross-platform downstream mediator (not a delivered metabolite)	[342,343,344,345,346]
Epigenetic aging (systems endpoint)	DNA methylation clocks; pace-of-aging networks	One DNAm clock + one pace-of-aging metric	Chronic/recurrent depression/anxiety; cardiometabolic comorbidity	Cross-platform; most informative in ≥6–8 week interventions	[347,348,349,350]
Confound control (must quantify)	Vasoactive and behavioral confounds; biogenic amines	Sodium per serving; histamine (minimum) ± tyramine	Prevents false symptom signals; protects vascular endpoint interpretation	Critical for kimchi, miso, aged cheeses, and spontaneous ferments; Brazil: tucupi; Canastra/Marajó	[72,91,107,109,351]

*, conceptual. DNAm, DNA methylation; FFAR2, free fatty acid receptor 2; FFAR3, free fatty acid receptor 3; GPR41, G protein-coupled receptor 41; GPR43, G protein-coupled receptor 43; HCAR2, hydroxycarboxylic acid receptor 2; HDAC, histone deacetylase; hsCRP, high-sensitivity C-reactive protein; IDO, indoleamine 2,3-dioxygenase; KYN, kynurenine; IL-6, interleukin-6; LBP, lipopolysaccharide-binding protein; NF-κB, nuclear factor kappa B; NOD, nucleotide-binding oligomerization domain receptor; PRR, pattern-recognition receptor; SCFAs, short-chain fatty acids; sCD14, soluble cluster of differentiation 14; sICAM-1, soluble intercellular adhesion molecule-1; sVCAM-1, soluble vascular cell adhesion molecule-1; TDO, tryptophan 2,3-dioxygenase; TLR, Toll-like receptor.

**Table 5:** Minimum viable mechanistic biomarker panel for fermented-food psychobiotic trials in depression and anxiety. The table outlines tiered biomarker sets—minimal, enhanced, and deluxe—covering clinical phenotype, metabolite modules, barrier/endotoxemia, endothelial activation, immunothrombosis, epigenetic aging, and product verification to support interpretable, mechanism-focused trials rather than symptom-only studies.

Domain	Minimal (Must-Have)	Enhanced (Strong Mechanistic Paper)	Deluxe (Multi-Omics, Grant-Ready)	Ref.
Clinical phenotype	Depression + anxiety scales; sleep quality; GI symptom index	Symptom clusters (anhedonia, anxious arousal); stress reactivity task	Digital phenotyping + actigraphy; ecological momentary assessment	[420,421,422]
Metabolites	Targeted SCFAs + Trp/KYN ratio	Indole panel + bile acid panel	Untargeted metabolomics + pathway enrichment	[423,424,425]
Barrier/endotoxemia	LBP and/or sCD14	Add fecal calprotectin; permeability assays in subsets	Microbiome functional profiling + mucosal markers (if feasible)	[150,426,427]
Endothelium	sICAM-1, sVCAM-1 (or similar activation markers)	Add glycocalyx shedding marker(s); oxidative stress marker	Endothelial transcriptomics (cells or proxies), vascular imaging endpoints	[307,428,429]
Immunothrombosis	Platelet activation (P-selectin or aggregation assay)	Platelet–leukocyte aggregates; NET proxy panel	Thrombin generation + platelet transcriptomics	[430,431,432]
Epigenetic aging	One clock + one pace-of-aging metric	≥2 clocks + inflammatory proteomics triangulation	Multi-tissue clocks (if available) + integrative modeling	[433,434,435]
Product verification	Batch composition + sodium + amine screen (if relevant)	Metabolite fingerprint per batch	Shotgun metagenomics of product + stability testing	[436,437,438]

GI, gastrointestinal; KYN, kynurenine, LBP, lipopolysaccharide-binding protein; NET, neutrophil extracellular trap; SCFAs, short-chain fatty acids; sCD14, soluble cluster of differentiation 14; sICAM-1, soluble intercellular adhesion molecule-1; sVCAM-1, soluble vascular cell adhesion molecule-1.

**Table 6:** Decisive trial templates for mechanism-first fermented-food psychobiotics in depression and anxiety. The table presents pragmatic, phenotype-enriched trial designs with matched comparators, required mechanistic signatures, minimal endpoints, and falsifiable hypotheses, enabling symptom changes to be interpreted through barrier, metabolite, endothelial, peptide, bile-acid, or platelet pathway engagement.

Decisive Trial	Enriched Phenotype	Arms (Dose, Duration)	Required Mechanistic Signature	Key Endpoints (Minimal)	Falsifiable Hypothesis	Ref.
Live vs. pasteurized yogurt/kefir RCT	Inflammatory depression (hsCRP/IL-6 high) ± sleep issues	Live vs. pasteurized comparator; 6–8 w; fixed g/day; sodium/sugar matched	Peptide/EPS engagement; endothelial activation falls when barrier/endotoxemia improves	Peptide/EPS; hsCRP or IL-6; LBP or sCD14; sICAM-1 or sVCAM-1; sleep/fatigue domains	H1: Domain improvement is mediated by peptide/EPS + Δendothelial activation, not macronutrients/expectancy.	[105,427,465,466]
Low-sodium vegetable ferment feeding RCT (kimchi/sauerkraut)	GI-linked anxiety and/or inflammatory depression	Live low-sodium ferment vs. pasteurized matched-acid control; 4–6 w; histamine/tyramine screened	ΔLBP/sCD14 required; downstream ΔsICAM-1/VCAM-1; SCFAs rise context-dependently	Sodium + amines; SCFAs; LBP or sCD14; optional calprotectin; sICAM-1/VCAM-1; anxious arousal	H2: If endotoxemia does not fall, symptom change will not exceed control.	[88,93,467,468,469]
Soy ferment RCT (tempeh/miso ± peptide-enriched arm)	Metabolic comorbidity + fatigue/somatic domain	Fermented soy vs. matched-protein non-fermented soy; 6–8 w; sodium matched (miso)	Peptidome shift; bile-acid module shift; improved redox/NO proxies	Peptides; bile acids; oxidative stress + NO proxy; hsCRP; fatigue/somatic domains	H3: Fatigue benefit requires bile-acid module shift; absent shift implies null.	[380,470,471,472,473]
Brazil cassava ferment crossover (tucupi or polvilho azedo)	High endotoxemia potential or GI-dominant symptoms	Cassava ferment vs. matched-carb control; 2–4 w/period; washout; strict product QC	Acid + starch-remodeling signature; ΔLBP/sCD14 and ΔsICAM-1/VCAM-1 define success	Product pH/TA + lactate/acetate; resistant-starch proxy; LBP/sCD14; sICAM-1/VCAM-1; GI index	H4: Upstream barrier engagement can be demonstrated even with modest global symptom change.	[67,77,474,475,476]
Embedded platelet sub-study	Inflammatory anxious arousal and/or vascular-risk depression	Platelet panel at baseline/end; standardize draw timing; no NSAID changes	Platelet activation falls only when endothelial activation falls	P-selectin (flow) or aggregation; sICAM-1/VCAM-1; hsCRP; stress reactivity task	H5: Platelet changes are downstream; mediate stress-reactivity only with Δendothelium.	[300,313,477,478,479]

EPS, exopolysaccharides; g/day, grams per day; GI, gastrointestinal; hsCRP, high-sensitivity C-reactive protein; IL-6, interleukin-6; LBP, lipopolysaccharide-binding protein; NO, nitric oxide; NSAID, nonsteroidal anti-inflammatory drug; pH, potential of hydrogen; QC, quality control; RCT, randomized controlled trial; SCFAs, short-chain fatty acids; sCD14, soluble cluster of differentiation 14; sICAM-1, soluble intercellular adhesion molecule-1; sVCAM-1, soluble vascular cell adhesion molecule-1; TA, titratable acidity; w, weeks; Δ, change in.

## Data Availability

No new data were created or analyzed in this study. Data sharing is not applicable to this article.

## Abbreviations

The following abbreviations are used in this manuscript:

ACE	angiotensin-converting enzyme
ADMA	asymmetric dimethylarginine
AhR	aryl hydrocarbon receptor
BBB	blood–brain barrier
BP	blood pressure
CA	cholic acid
CDCA	chenodeoxycholic acid
CNS	central nervous system
CRP	C-reactive protein
DCA	deoxycholic acid
DPP-IV	dipeptidyl peptidase-4
eNOS	endothelial nitric oxide synthase
EPS	exopolysaccharides
FFAR2	free fatty acid receptor 2 (GPR43)
FFAR3	free fatty acid receptor 3 (GPR41)
FGF19	fibroblast growth factor 19
FITC	fluorescein isothiocyanate
FMD	flow-mediated dilation
FXR	Farnesoid X Receptor
GFAP	glial fibrillary acidic protein
GLP-1	glucagon-like peptide-1
GPCR	G protein-coupled receptor
GPR109A	G protein-coupled receptor 109A (HCAR2)
HDAC	histone deacetylase
HOMA-IR	homeostatic model assessment of insulin resistance
HPA	hypothalamic–pituitary–adrenal
I-FABP	intestinal fatty acid-binding protein
IAA	indole-3-acetic acid
ICAM-1	intercellular adhesion molecule 1
IL	interleukin
ILA	indole-3-lactic acid
IPA	indole-3-propionic acid
KYN	kynurenine
LAB	lactic acid bacteria
LBP	lipopolysaccharide-binding protein
LC-MS	liquid chromatography–mass spectrometry
LCA	lithocholic acid
LPS	lipopolysaccharide
MAOI	monoamine oxidase inhibitor
MAPK	mitogen-activated protein kinase
MDD	major depressive disorder
MMP-9	matrix metalloproteinase 9
MPO	myeloperoxidase
mTOR	mechanistic target of rapamycin
NET	neutrophil extracellular trap
NF-κB	nuclear factor kappa B
NO	nitric oxide
NOX2	NADPH oxidase 2
Nrf2	nuclear factor erythroid 2-related factor 2
PF4	platelet factor 4
PI3K	phosphoinositide 3-kinase
PRR	pattern-recognition receptor
QC	quality control
RAAS	renin–angiotensin–aldosterone system
Reg3	regenerating islet-derived protein 3 family
ROS	reactive oxygen species
sCD14	soluble cluster of differentiation 14
SCFA	short-chain fatty acids
sICAM-1	soluble intercellular adhesion molecule 1
sVCAM-1	soluble vascular cell adhesion molecule 1
TEER	transepithelial electrical resistance
TGR5	Takeda G protein-coupled receptor 5
Th17	T helper 17 cell
TLR	Toll-like receptor
TNF-α	tumor necrosis factor alpha
Trp	tryptophan
UDCA	ursodeoxycholic acid
VEGF	vascular endothelial growth factor
vWF	von Willebrand factor
ZO-1	zonula occludens 1

## References

[1] Menard C., Pfau M.L., Hodes G.E., Kana V., Wang V.X., Bouchard S., Takahashi A., Flanigan M.E., Aleyasin H., LeClair K.B. (2017). Social stress induces neurovascular pathology promoting depression. Nat. Neurosci..

[2] Taylor W.D., Aizenstein H.J., Alexopoulos G.S. (2013). The vascular depression hypothesis: Mechanisms linking vascular disease with depression. Mol. Psychiatry.

[3] Tanaka M. (2025). From Monoamines to Systems Psychiatry: Rewiring Depression Science and Care (1960s-2025). Biomedicines.

[4] Chehadi A.C., de Lima E.P., Detregiachi C.R.P., Haber R.S.d.A., Catharin V.M.C.S., Laurindo L.F., Valenti V.E., Galhardi C.M., Tanaka M., Barbalho S.M. (2026). Harnessing Dietary Tryptophan: Bridging the Gap Between Neurobiology and Psychiatry in Depression Management. Int. J. Mol. Sci..

[5] Tanaka M., Vécsei L. (2026). The kynurenine pathway in depression and schizophrenia: Convergent signals, divergent states, and clinical signatures. EXCLI J..

[6] Balasubramanian R., Schneider E., Gunnigle E., Cotter P.D., Cryan J.F. (2024). Fermented foods: Harnessing their potential to modulate the microbiota-gut-brain axis for mental health. Neurosci. Biobehav. Rev..

[7] Palepu M.S.K., Dandekar M.P. (2022). Remodeling of microbiota gut-brain axis using psychobiotics in depression. Eur. J. Pharmacol..

[8] Ribera C., Sánchez-Ortí J.V., Clarke G., Marx W., Mörkl S., Balanzá-Martínez V. (2024). Probiotic, prebiotic, synbiotic and fermented food supplementation in psychiatric disorders: A systematic review of clinical trials. Neurosci. Biobehav. Rev..

[9] Puglisi V., Bramanti A., Lanza G., Cantone M., Vinciguerra L., Pennisi M., Bonanno L., Pennisi G., Bella R. (2018). Impaired Cerebral Haemodynamics in Vascular Depression: Insights From Transcranial Doppler Ultrasonography. Front. Psychiatry.

[10] Izzi B., Tirozzi A., Cerletti C., Donati M.B., de Gaetano G., Hoylaerts M.F., Iacoviello L., Gialluisi A. (2020). Beyond Haemostasis and Thrombosis: Platelets in Depression and Its Co-Morbidities. Int. J. Mol. Sci..

[11] Wu S., Zhang Y., Lu Y., Yin Y., Yang C., Tang W., Song T., Tao X., Wang Q. (2025). Vascular depression: A comprehensive exploration of the definition, mechanisms, and clinical challenges. Neurobiol. Dis..

[12] Jellinger K.A. (2022). The enigma of vascular depression in old age: A critical update. J. Neural Transm..

[13] Tanaka M., Battaglia S., Liloia D. (2025). Navigating Neurodegeneration: Integrating Biomarkers, Neuroinflammation, and Imaging in Parkinson’s, Alzheimer’s, and Motor Neuron Disorders. Biomedicines.

[14] Dudek K.A., Dion-Albert L., Lebel M., LeClair K., Labrecque S., Tuck E., Ferrer Perez C., Golden S.A., Tamminga C., Turecki G. (2020). Molecular adaptations of the blood-brain barrier promote stress resilience vs. depression. Proc. Natl. Acad. Sci. USA.

[15] Tanaka M., He Z., Han S., Battaglia S. (2025). Editorial: Noninvasive brain stimulation: A promising approach to study and improve emotion regulation. Front. Behav. Neurosci..

[16] Szabó Á., Galla Z., Spekker E., Szűcs M., Martos D., Takeda K., Ozaki K., Inoue H., Yamamoto S., Toldi J. (2025). Oxidative and Excitatory Neurotoxic Stresses in CRISPR/Cas9-Induced Kynurenine Aminotransferase Knockout Mice: A Novel Model for Despair-Based Depression and Post-Traumatic Stress Disorder. Front. Biosci. Landmark Ed..

[17] Zhang X., Wang Y., Xue S., Gong L., Yan J., Zheng Y., Yang X., Fan Y., Han K., Chen Y. (2024). Chronic stress in adulthood results in microvascular dysfunction and subsequent depressive-like behavior. Sci. Rep..

[18] Najjar S., Pearlman D.M., Devinsky O., Najjar A., Zagzag D. (2013). Neurovascular unit dysfunction with blood-brain barrier hyperpermeability contributes to major depressive disorder: A review of clinical and experimental evidence. J. Neuroinflamm..

[19] Yan C., Wu H., Fang X., He J., Zhu F. (2023). Platelet, a key regulator of innate and adaptive immunity. Front. Med..

[20] Mandel J., Casari M., Stepanyan M., Martyanov A., Deppermann C. (2022). Beyond Hemostasis: Platelet Innate Immune Interactions and Thromboinflammation. Int. J. Mol. Sci..

[21] Tanaka M., Battaglia S. (2025). From Biomarkers to Behavior: Mapping the Neuroimmune Web of Pain, Mood, and Memory. Biomedicines.

[22] Jia L., Xiao L., Fu Y., Shao Z., Jing Z., Yuan J., Xie Y., Guo J., Wang Y., Geng W. (2024). Neuroprotective effects of probiotics on anxiety- and depression-like disorders in stressed mice by modulating tryptophan metabolism and the gut microbiota. Food Funct..

[23] Stark K., Massberg S. (2021). Interplay between inflammation and thrombosis in cardiovascular pathology. Nat. Rev. Cardiol..

[24] Aghara H., Patel M., Chadha P., Parwani K., Chaturvedi R., Mandal P. (2025). Unraveling the Gut-Liver-Brain Axis: Microbiome, Inflammation, and Emerging Therapeutic Approaches. Mediat. Inflamm..

[25] van de Wouw M., Walsh A.M., Crispie F., van Leuven L., Lyte J.M., Boehme M., Clarke G., Dinan T.G., Cotter P.D., Cryan J.F. (2020). Distinct actions of the fermented beverage kefir on host behaviour, immunity and microbiome gut-brain modules in the mouse. Microbiome.

[26] Schneider E., Balasubramanian R., Ferri A., Cotter P.D., Clarke G., Cryan J.F. (2025). Fibre & fermented foods: Differential effects on the microbiota-gut-brain axis. Proc. Nutr. Soc..

[27] O’Riordan K.J., Collins M.K., Moloney G.M., Knox E.G., Aburto M.R., Fülling C., Morley S.J., Clarke G., Schellekens H., Cryan J.F. (2022). Short chain fatty acids: Microbial metabolites for gut-brain axis signalling. Mol. Cell. Endocrinol..

[28] Aslam H., Green J., Jacka F.N., Collier F., Berk M., Pasco J., Dawson S.L. (2020). Fermented foods, the gut and mental health: A mechanistic overview with implications for depression and anxiety. Nutr. Neurosci..

[29] Śliwka A., Polak-Berecka M., Zdybel K., Zelek-Molik A., Waśko A. (2025). Psychobiotics in Depression: Sources, Metabolites, and Treatment—A Systematic Review. Nutrients.

[30] Tanaka M. (2026). Synaptic Plasticity—Intrinsic Excitability and Antidepressant Discovery. Biomedicines.

[31] Ji X., Wang J., Lan T., Zhao D., Xu P. (2025). Gut microbial metabolites and the brain-gut axis in Alzheimer’s disease: A review. Biomol. Biomed..

[32] Vich Vila A., Collij V., Sanna S., Sinha T., Imhann F., Bourgonje A.R., Mujagic Z., Jonkers D., Masclee A.A.M., Fu J. (2020). Impact of commonly used drugs on the composition and metabolic function of the gut microbiota. Nat. Commun..

[33] Li Y., Zhang L., Xu X., Lin S., Xie J., Li J. (2025). Formation Mechanism and Nutritional Effects of Organic Acids in Fermented Foods: A Review. J. Food Sci..

[34] Gautam A., Poopalarajah R., Ahmad A.R., Rana B.N., Denekew T.W., Ahn N., Utenova L., Kunwor Y.S., Bhandari N.N., Jha A.R. (2025). Ecological factors that drive microbial communities in culturally diverse fermented foods. BMC Microbiol..

[35] Almahal Z.H., Hasan A., Razzak S.A., Nzila A., Uddin S. (2025). Molecular Perspective of Dietary Influences on the Gut Microbiome alongside Neurological Health: Exploring the Gut-Brain Axis. ACS Chem. Neurosci..

[36] Del Toro-Barbosa M., Hurtado-Romero A., Garcia-Amezquita L.E., García-Cayuela T. (2020). Psychobiotics: Mechanisms of Action, Evaluation Methods and Effectiveness in Applications with Food Products. Nutrients.

[37] Liloia D., Rocca P., Brasso C., Tanaka M., Manuello J., Crocetta A., Duca S., Costa T., Cauda F. (2026). Identification of a diagnosis-selective neurobiological substrate for bipolar disorder, major depressive disorder, and schizophrenia: A meta-analysis of 57,717 subjects. Psychol. Med..

[38] Ahmed H., Leyrolle Q., Koistinen V., Kärkkäinen O., Layé S., Delzenne N., Hanhineva K. (2022). Microbiota-derived metabolites as drivers of gut-brain communication. Gut Microbes.

[39] Cheng L.H., Liu Y.W., Wu C.C., Wang S., Tsai Y.C. (2019). Psychobiotics in mental health, neurodegenerative and neurodevelopmental disorders. J. Food Drug Anal..

[40] Tanaka M., Battaglia S. (2025). Dualistic Dynamics in Neuropsychiatry: From Monoaminergic Modulators to Multiscale Biomarker Maps. Biomedicines.

[41] Tanaka M. (2025). From Serendipity to Precision: Integrating AI, Multi-Omics, and Human-Specific Models for Personalized Neuropsychiatric Care. Biomedicines.

[42] Peluzio M., Dias M.M.E., Martinez J.A., Milagro F.I. (2021). Kefir and Intestinal Microbiota Modulation: Implications in Human Health. Front. Nutr..

[43] Tanaka M., Araujo A.C., Valenti V.E., Guiguer E.L., Catharin V.C.S., Gualhardi C.M., de Souza Bastos Mazuqueli Pereira E., de Alvares Goulart R., de Argolo Haber R.S., de Carvalho A.C.A. (2026). From Polyphenols to Prodrugs: Bridging the Blood-Brain Barrier with Nanomedicine and Neurotherapeutics. Int. J. Mol. Sci..

[44] Caffrey E.B., Sonnenburg J.L., Devkota S. (2024). Our extended microbiome: The human-relevant metabolites and biology of fermented foods. Cell Metab..

[45] Sampsell K., Marcolla C.S., Tapping S., Fan Y., Sánchez-Lafuente C.L., Willing B.P., Reimer R.A., Burton J.P. (2025). Current Research in Fermented Foods: Bridging Tradition and Science. Adv. Nutr..

[46] Rusch J.A., Layden B.T., Dugas L.R. (2023). Signalling cognition: The gut microbiota and hypothalamic-pituitary-adrenal axis. Front. Endocrinol..

[47] Park I., Mannaa M. (2025). Fermented Foods as Functional Systems: Microbial Communities and Metabolites Influencing Gut Health and Systemic Outcomes. Foods.

[48] Caspani G., Kennedy S., Foster J.A., Swann J. (2019). Gut microbial metabolites in depression: Understanding the biochemical mechanisms. Microb. Cell.

[49] van der Hee B., Wells J.M. (2021). Microbial Regulation of Host Physiology by Short-chain Fatty Acids. Trends Microbiol..

[50] Verni M., Verardo V., Rizzello C.G. (2019). How Fermentation Affects the Antioxidant Properties of Cereals and Legumes. Foods.

[51] Baxter N.T., Schmidt A.W., Venkataraman A., Kim K.S., Waldron C., Schmidt T.M. (2019). Dynamics of Human Gut Microbiota and Short-Chain Fatty Acids in Response to Dietary Interventions with Three Fermentable Fibers. mBio.

[52] Wei S., Wang C., Zhang Q., Yang H., Deehan E.C., Zong X., Wang Y., Jin M. (2022). Dynamics of microbial communities during inulin fermentation associated with the temporal response in SCFA production. Carbohydr. Polym..

[53] Sarkar S., Sha S.P., Ghatani K. (2023). Metabolomics of ethnic fermented foods and beverages: Understanding new aspects through Omic techniques. Front. Sustain. Food Syst..

[54] Balcázar-Zumaeta C.R., Castro-Alayo E.M., Cayo-Colca I.S., Idrogo-Vásquez G., Muñoz-Astecker L.D. (2023). Metabolomics during the spontaneous fermentation in cocoa (*Theobroma cacao* L.): An exploraty review. Food Res. Int..

[55] Yang F., Chen C., Ni D., Yang Y., Tian J., Li Y., Chen S., Ye X., Wang L. (2023). Effects of Fermentation on Bioactivity and the Composition of Polyphenols Contained in Polyphenol-Rich Foods: A Review. Foods.

[56] Utpott M., Rodrigues E., Rios A.O., Mercali G.D., Flôres S.H. (2022). Metabolomics: An analytical technique for food processing evaluation. Food Chem..

[57] Liu H., Liao C., Wu L., Tang J., Chen J., Lei C., Zheng L., Zhang C., Liu Y.Y., Xavier J. (2022). Ecological dynamics of the gut microbiome in response to dietary fiber. ISME J..

[58] Wen L., Yang L., Chen C., Li J., Fu J., Liu G., Kan Q., Ho C.T., Huang Q., Lan Y. (2024). Applications of multi-omics techniques to unravel the fermentation process and the flavor formation mechanism in fermented foods. Crit. Rev. Food Sci. Nutr..

[59] García-Burgos M., Moreno-Fernández J., Alférez M.J., Díaz-Castro J., López-Aliaga I. (2020). New perspectives in fermented dairy products and their health relevance. J. Funct. Foods.

[60] Şanlier N., Gökcen B.B., Sezgin A.C. (2019). Health benefits of fermented foods. Crit. Rev. Food Sci. Nutr..

[61] Parvez S., Malik K.A., Ah Kang S., Kim H.Y. (2006). Probiotics and their fermented food products are beneficial for health. J. Appl. Microbiol..

[62] do Prado F.G., Pagnoncelli M.G.B., de Melo Pereira G.V., Karp S.G., Soccol C.R. (2022). Fermented soy products and their potential health benefits: A review. Microorganisms.

[63] Harahap I.A., Suliburska J., Karaca A.C., Capanoglu E., Esatbeyoglu T. (2025). Fermented soy products: A review of bioactives for health from fermentation to functionality. Compr. Rev. Food Sci. Food Saf..

[64] Gao Y., Hou L., Gao J., Li D., Tian Z., Fan B., Wang F., Li S. (2021). Metabolomics Approaches for the Comprehensive Evaluation of Fermented Foods: A Review. Foods.

[65] Peres Fabbri L., Cavallero A., Vidotto F., Gabriele M. (2024). Bioactive Peptides from Fermented Foods: Production Approaches, Sources, and Potential Health Benefits. Foods.

[66] Freire A.L., Ramos C.L., Schwan R.F. (2015). Microbiological and chemical parameters during cassava based-substrate fermentation using potential starter cultures of lactic acid bacteria and yeast. Food Res. Int..

[67] Penido F.C.L., Piló F.B., Sandes S.H.C., Nunes Á.C., Colen G., Oliveira E.S., Rosa C.A., Lacerda I.C.A. (2018). Selection of starter cultures for the production of sour cassava starch in a pilot-scale fermentation process. Braz. J. Microbiol..

[68] Sionek B., Szydłowska A., Küçükgöz K., Kołożyn-Krajewska D. (2023). Traditional and new microorganisms in lactic acid fermentation of food. Fermentation.

[69] Caplice E., Fitzgerald G.F. (1999). Food fermentations: Role of microorganisms in food production and preservation. Int. J. Food Microbiol..

[70] Lima T.T.M., Hosken B.d.O., Venturim B.C., Lopes I.L., Martin J.G.P. (2022). Traditional Brazilian fermented foods: Cultural and technological aspects. J. Ethn. Foods.

[71] MAYORGA G.A.C., PALMA G.B.A., Sandoval-Cañas G.J., Ordoñez-Araque R.H. (2020). Ancestral fermented indigenous beverages from South America made from cassava (*Manihot esculenta*). Food Sci. Technol..

[72] do Carmo Brito B.N., Campos Chisté R., Santos Lopes A., Abreu Glória M.B., da Silva Pena R. (2019). Influence of spontaneous fermentation of manipueira on bioactive amine and carotenoid profiles during tucupi production. Food Res. Int..

[73] Brito B., Martins M.G., Chisté R.C., Lopes A.S., Gloria M.B.A., Pena R.D.S. (2023). Total and Free Hydrogen Cyanide Content and Profile of Bioactive Amines in Commercial Tucupi, a Traditionally Derived Cassava Product Widely Consumed in Northern Brazil. Foods.

[74] Malongane F., Berejena T. (2024). Exploring the microbiome present in fermented indigenous African foods and their potential impact on human health. J. Agric. Food Res..

[75] de Oliveira Gonçalves L., São Julião S.M., Zago L., Santana I. (2024). Cassava, an illustrious (un) known: Consumption of recipes with the root and its derived products. Int. J. Gastron. Food Sci..

[76] Ferraro V., Piccirillo C., Tomlins K., Pintado M.E. (2016). Cassava (*Manihot esculenta* Crantz) and yam (*Dioscorea* spp.) crops and their derived foodstuffs: Safety, security and nutritional value. Crit. Rev. Food Sci. Nutr..

[77] Mafaldo Í.M., Araújo L.M., Cabral L., Barão C.E., Noronha M.F., Fink J.R., de Albuquerque T.M.R., Dos Santos Lima M., Vidal H., Pimentel T.C. (2024). Cassava (*Manihot esculenta*) Brazilian cultivars have different chemical compositions, present prebiotic potential, and beneficial effects on the colonic microbiota of celiac individuals. Food Res. Int..

[78] Hosken B.d.O., Melo Pereira G.V., Lima T.T.M., Ribeiro J.B., Magalhães Júnior W.C.P.d., Martin J.G.P. (2023). Underexplored potential of lactic acid bacteria associated with artisanal cheese making in Brazil: Challenges and opportunities. Fermentation.

[79] Penna A.L.B., Gigante M.L., Todorov S.D. (2021). Artisanal Brazilian Cheeses-History, Marketing, Technological and Microbiological Aspects. Foods.

[80] Margalho L.P., Feliciano M.D., Silva C.E., Abreu J.S., Piran M.V.F., Sant’Ana A.S. (2020). Brazilian artisanal cheeses are rich and diverse sources of nonstarter lactic acid bacteria regarding technological, biopreservative, and safety properties-Insights through multivariate analysis. J. Dairy Sci..

[81] Pineda A.P.A., Campos G.Z., Pimentel-Filho N.J., Franco B., Pinto U.M. (2021). Brazilian Artisanal Cheeses: Diversity, Microbiological Safety, and Challenges for the Sector. Front. Microbiol..

[82] Bezerra D., Souza K.M.S., Sales D.C., Araújo E.O.M., Urbano S.A., Cipolat-Gotet C., Anaya K., Ribeiro C., Porto A.L.F., Rangel A.H.N. (2024). Effect of ripening time on the content of bioactive peptides and fatty acids profile of Artisanal Coalho cheese. PLoS ONE.

[83] Campagnollo F.B., Margalho L.P., Kamimura B.A., Feliciano M.D., Freire L., Lopes L.S., Alvarenga V.O., Cadavez V.A.P., Gonzales-Barron U., Schaffner D.W. (2018). Selection of indigenous lactic acid bacteria presenting anti-listerial activity, and their role in reducing the maturation period and assuring the safety of traditional Brazilian cheeses. Food Microbiol..

[84] Zheng X., Xu X., Ma Y., Zhu L., Xiao J., Deng L., Shi X., Wang B. (2021). Diversity and potential function of bacterial communities during milk fermentation of Kazak artisanal cheese. Process Biochem..

[85] Van Hoorde K., Van Leuven I., Dirinck P., Heyndrickx M., Coudijzer K., Vandamme P., Huys G. (2010). Selection, application and monitoring of *Lactobacillus paracasei* strains as adjunct cultures in the production of Gouda-type cheeses. Int. J. Food Microbiol..

[86] Mangione G., Caccamo M., Marino V.M., Marino G., Licitra G. (2023). Characterization of artisanal saffron ricotta cheese produced in Sicily: Physicochemical, microbiological, sensory, and antioxidant characteristics. J. Dairy. Sci..

[87] de Albuquerque T., Campos G.Z., d’Ovidio L., Pinto U.M., Sobral P., Galvão J.A. (2024). Unveiling Safety Concerns in Brazilian Artisanal Cheeses: A Call for Enhanced Ripening Protocols and Microbiological Assessments. Foods.

[88] Fijan S., Fijan P., Wei L., Marco M.L. (2024). Health benefits of kimchi, sauerkraut, and other fermented foods of the genus brassica. Appl. Microbiol..

[89] Tan X., Cui F., Wang D., Lv X., Li X., Li J. (2023). Fermented Vegetables: Health Benefits, Defects, and Current Technological Solutions. Foods.

[90] Rezac S., Kok C.R., Heermann M., Hutkins R. (2018). Fermented Foods as a Dietary Source of Live Organisms. Front. Microbiol..

[91] Saha Turna N., Chung R., McIntyre L. (2024). A review of biogenic amines in fermented foods: Occurrence and health effects. Heliyon.

[92] Barbieri F., Montanari C., Gardini F., Tabanelli G. (2019). Biogenic Amine Production by Lactic Acid Bacteria: A Review. Foods.

[93] Dimidi E., Cox S.R., Rossi M., Whelan K. (2019). Fermented Foods: Definitions and Characteristics, Impact on the Gut Microbiota and Effects on Gastrointestinal Health and Disease. Nutrients.

[94] Abdul Hakim B.N., Xuan N.J., Oslan S.N.H. (2023). A Comprehensive Review of Bioactive Compounds from Lactic Acid Bacteria: Potential Functions as Functional Food in Dietetics and the Food Industry. Foods.

[95] Kaur H., Kaur G., Ali S.A. (2022). Dairy-based probiotic-fermented functional foods: An update on their health-promoting properties. Fermentation.

[96] Bourrie B.C., Willing B.P., Cotter P.D. (2016). The Microbiota and Health Promoting Characteristics of the Fermented Beverage Kefir. Front. Microbiol..

[97] Nyanzi R., Jooste P.J., Buys E.M. (2021). Invited review: Probiotic yogurt quality criteria, regulatory framework, clinical evidence, and analytical aspects. J. Dairy Sci..

[98] Paul A.K., Lim C.L., Apu M.A.I., Dolma K.G., Gupta M., de Lourdes Pereira M., Wilairatana P., Rahmatullah M., Wiart C., Nissapatorn V. (2023). Are Fermented Foods Effective against Inflammatory Diseases?. Int. J. Environ. Res. Public Health.

[99] Terpou A., Dahiya D., Nigam P.S. (2025). Evolving Dynamics of Fermented Food Microbiota and the Gut Microenvironment: Strategic Pathways to Enhance Human Health. Foods.

[100] Li X., Yao Z., Qian J., Li H., Li H. (2024). Lactate Protects Intestinal Epithelial Barrier Function from Dextran Sulfate Sodium-Induced Damage by GPR81 Signaling. Nutrients.

[101] Rosa D.D., Dias M.M.S., Grześkowiak Ł.M., Reis S.A., Conceição L.L., Peluzio M. (2017). Milk kefir: Nutritional, microbiological and health benefits. Nutr. Res. Rev..

[102] Vieira C.P., Rosario A., Lelis C.A., Rekowsky B.S.S., Carvalho A.P.A., Rosário D.K.A., Elias T.A., Costa M.P., Foguel D., Conte-Junior C.A. (2021). Bioactive Compounds from Kefir and Their Potential Benefits on Health: A Systematic Review and Meta-Analysis. Oxid. Med. Cell. Longev..

[103] Caffrey E.B., Perelman D., Ward C.P., Sonnenburg E.D., Gardner C.D., Sonnenburg J.L. (2025). Unpacking Food Fermentation: Clinically Relevant Tools for Fermented Food Identification and Consumption. Adv. Nutr..

[104] Paveljšek D., Pertziger E., Fardet A., Panagiotakos D.B., Savary-Auzeloux I., Adamberg S., Peñas E., Frias J., Ntantou A., Diamantoglou I. (2025). A systematic review of prospective evidence linking non-alcoholic fermented food consumption with lower mortality risk. Front. Nutr..

[105] Gao Y., Liu Y., Ma T., Liang Q., Sun J., Wu X., Song Y., Nie H., Huang J., Mu G. (2025). Fermented Dairy Products as Precision Modulators of Gut Microbiota and Host Health: Mechanistic Insights, Clinical Evidence, and Future Directions. Foods.

[106] Todorovic S., Akpinar A., Assunção R., Bär C., Bavaro S.L., Berkel Kasikci M., Domínguez-Soberanes J., Capozzi V., Cotter P.D., Doo E.H. (2024). Health benefits and risks of fermented foods-the PIMENTO initiative. Front. Nutr..

[107] Hazards E.P.O.B. (2011). Scientific opinion on risk based control of biogenic amine formation in fermented foods. EFSA J..

[108] De B.J., Bell J.W., Nolte F., Arcieri J., Correa G. (2021). Histamine Limits by Country: A Survey and Review. J. Food Prot..

[109] Dala-Paula B.M., Custódio F.B., Gloria M.B. (2023). Health concerns associated with biogenic amines in food and interaction with amine oxidase drugs. Curr. Opin. Food Sci..

[110] Patik J.C., Lennon S.L., Farquhar W.B., Edwards D.G. (2021). Mechanisms of Dietary Sodium-Induced Impairments in Endothelial Function and Potential Countermeasures. Nutrients.

[111] Boegehold M.A. (2013). The effect of high salt intake on endothelial function: Reduced vascular nitric oxide in the absence of hypertension. J. Vasc. Res..

[112] Farquhar W.B., Edwards D.G., Jurkovitz C.T., Weintraub W.S. (2015). Dietary sodium and health: More than just blood pressure. J. Am. Coll. Cardiol..

[113] DuPont J.J., Greaney J.L., Wenner M.M., Lennon-Edwards S.L., Sanders P.W., Farquhar W.B., Edwards D.G. (2013). High dietary sodium intake impairs endothelium-dependent dilation in healthy salt-resistant humans. J. Hypertens..

[114] Grillo A., Salvi L., Coruzzi P., Salvi P., Parati G. (2019). Sodium Intake and Hypertension. Nutrients.

[115] Stratton J.E., Hutkins R.W., Taylor S.L. (1991). Biogenic Amines in Cheese and other Fermented Foods: A Review. J. Food Prot..

[116] Mikhailidis D.P., Jeremy J.Y., Barradas M.A., Green N., Dandona P. (1983). Effect of ethanol on vascular prostacyclin (prostaglandin I2) synthesis, platelet aggregation, and platelet thromboxane release. Br. Med. J. Clin. Res. Ed..

[117] Salem R.O., Laposata M. (2005). Effects of alcohol on hemostasis. Am. J. Clin. Pathol..

[118] Muzaifa M., Abubakar Y., Safrida, Nilda C., Sapitri M. Alcohol content and chemical characteristics of fermented beverages in Aceh Province-Indonesia. Proceedings of the IOP Conference Series: Earth and Environmental Science.

[119] Gardiner C., Weakley J., Burke L.M., Roach G.D., Sargent C., Maniar N., Huynh M., Miller D.J., Townshend A., Halson S.L. (2025). The effect of alcohol on subsequent sleep in healthy adults: A systematic review and meta-analysis. Sleep Med. Rev..

[120] Guerzoni S., Pellesi L., Pini L.A., Caputo F. (2018). Drug-drug interactions in the treatment for alcohol use disorders: A comprehensive review. Pharmacol. Res..

[121] Panyod S., Wu W.K., Chang C.T., Wada N., Ho H.C., Lo Y.L., Tsai S.P., Chen R.A., Huang H.S., Liu P.Y. (2024). Common dietary emulsifiers promote metabolic disorders and intestinal microbiota dysbiosis in mice. Commun. Biol..

[122] Bancil A.S., Sandall A.M., Rossi M., Chassaing B., Lindsay J.O., Whelan K. (2021). Food Additive Emulsifiers and Their Impact on Gut Microbiome, Permeability, and Inflammation: Mechanistic Insights in Inflammatory Bowel Disease. J. Crohns Colitis.

[123] Shil A., Olusanya O., Ghufoor Z., Forson B., Marks J., Chichger H. (2020). Artificial Sweeteners Disrupt Tight Junctions and Barrier Function in the Intestinal Epithelium through Activation of the Sweet Taste Receptor, T1R3. Nutrients.

[124] Ruiz-Ojeda F.J., Plaza-Díaz J., Sáez-Lara M.J., Gil A. (2019). Effects of Sweeteners on the Gut Microbiota: A Review of Experimental Studies and Clinical Trials. Adv. Nutr..

[125] Dias P.G.I., Sajiwani J.W.A., Rathnayaka R. (2020). Consumer perception and sensory profile of probiotic yogurt with added sugar and reduced milk fat. Heliyon.

[126] Walter J., Maldonado-Gómez M.X., Martínez I. (2018). To engraft or not to engraft: An ecological framework for gut microbiome modulation with live microbes. Curr. Opin. Biotechnol..

[127] Fiore W., Arioli S., Guglielmetti S. (2020). The Neglected Microbial Components of Commercial Probiotic Formulations. Microorganisms.

[128] Ashrafian F., Keshavarz Azizi Raftar S., Shahryari A., Behrouzi A., Yaghoubfar R., Lari A., Moradi H.R., Khatami S., Omrani M.D., Vaziri F. (2021). Comparative effects of alive and pasteurized *Akkermansia muciniphila* on normal diet-fed mice. Sci. Rep..

[129] Pessione E., Cirrincione S. (2016). Bioactive Molecules Released in Food by Lactic Acid Bacteria: Encrypted Peptides and Biogenic Amines. Front. Microbiol..

[130] Skowron K., Budzyńska A., Grudlewska-Buda K., Wiktorczyk-Kapischke N., Andrzejewska M., Wałecka-Zacharska E., Gospodarek-Komkowska E. (2022). Two Faces of Fermented Foods-The Benefits and Threats of Its Consumption. Front. Microbiol..

[131] Mohr A.E., Pyne D.B., Leite G.S.F., Akins D., Pugh J. (2024). A systematic scoping review of study methodology for randomized controlled trials investigating probiotics in athletic and physically active populations. J. Sport Health Sci..

[132] Ouwehand A.C. (2017). A review of dose-responses of probiotics in human studies. Benef. Microbes.

[133] Fernández-Rodríguez D., Bravo M.C., Pizarro M., Vergara-Barra P., Hormazábal M.J., Leonario-Rodriguez M. (2025). Efficacy of *Lactobacillus* spp. Interventions to Modulate Mood Symptoms: A Scoping Review of Clinical Trials. Int. J. Mol. Sci..

[134] West N.P., Hughes L., Ramsey R., Zhang P., Martoni C.J., Leyer G.J., Cripps A.W., Cox A.J. (2020). Probiotics, Anticipation Stress, and the Acute Immune Response to Night Shift. Front. Immunol..

[135] ÇOLAK H., UĞUR M. (2002). The effect of different temperature and time in storage on the formation of biogenic amines in fermented sucuks. Turk. J. Vet. Anim. Sci..

[136] Ferdousi R., Rouhi M., Mohammadi R., Mortazavian A.M., Khosravi-Darani K., Rad A.H. (2013). Evaluation of probiotic survivability in yogurt exposed to cold chain interruption. Iran. J. Pharm. Res. IJPR.

[137] Cabello-Olmo M., Oneca M., Torre P., Díaz J.V., Encio I.J., Barajas M., Araña M. (2020). Influence of Storage Temperature and Packaging on Bacteria and Yeast Viability in a Plant-Based Fermented Food. Foods.

[138] Binder L.B., Menegatti Bevilacqua L., Gro Lorentzen Thomassen F., Lebel M., Jensen B.A.H., Menard C. (2025). Across Barriers: Blood-Brain and Gut Barrier Signaling in Psychiatric Disorders. J. Neurochem..

[139] Morys J., Małecki A., Nowacka-Chmielewska M. (2024). Stress and the gut-brain axis: An inflammatory perspective. Front. Mol. Neurosci..

[140] Masanetz R.K., Winkler J., Winner B., Günther C., Süß P. (2022). The Gut-Immune-Brain Axis: An Important Route for Neuropsychiatric Morbidity in Inflammatory Bowel Disease. Int. J. Mol. Sci..

[141] Di Sabatino A., Santacroce G., Rossi C.M., Broglio G., Lenti M.V. (2023). Role of mucosal immunity and epithelial-vascular barrier in modulating gut homeostasis. Intern. Emerg. Med..

[142] Grover M., Vanuytsel T., Chang L. (2025). Intestinal Permeability in Disorders of Gut-Brain Interaction: From Bench to Bedside. Gastroenterology.

[143] Zhao H., Tao L., Tang C., Cai W., Shen W. (2025). Do immune system and microbiome-gut-brain axis interactions associate with major depressive disorder?. J. Transl. Med..

[144] Abraham C., Abreu M.T., Turner J.R. (2022). Pattern Recognition Receptor Signaling and Cytokine Networks in Microbial Defenses and Regulation of Intestinal Barriers: Implications for Inflammatory Bowel Disease. Gastroenterology.

[145] Chen R., Zou J., Chen J., Zhong X., Kang R., Tang D. (2025). Pattern recognition receptors: Function, regulation and therapeutic potential. Signal Transduct. Target. Ther..

[146] Fitzgerald K.A., Kagan J.C. (2020). Toll-like Receptors and the Control of Immunity. Cell.

[147] Vanuytsel T., Bercik P., Boeckxstaens G. (2023). Understanding neuroimmune interactions in disorders of gut-brain interaction: From functional to immune-mediated disorders. Gut.

[148] Na K., Oh B.C., Jung Y. (2023). Multifaceted role of CD14 in innate immunity and tissue homeostasis. Cytokine Growth Factor Rev..

[149] Feng S., Zhang C., Chen S., He R., Chao G., Zhang S. (2023). TLR5 Signaling in the Regulation of Intestinal Mucosal Immunity. J. Inflamm. Res..

[150] Stevens B.R., Goel R., Seungbum K., Richards E.M., Holbert R.C., Pepine C.J., Raizada M.K. (2018). Increased human intestinal barrier permeability plasma biomarkers zonulin and FABP2 correlated with plasma LPS and altered gut microbiome in anxiety or depression. Gut.

[151] Wu H., Wang J., Teng T., Yin B., He Y., Jiang Y., Liu X., Yu Y., Li X., Zhou X. (2023). Biomarkers of intestinal permeability and blood-brain barrier permeability in adolescents with major depressive disorder. J. Affect. Disord..

[152] Yang S., Yu M. (2021). Role of Goblet Cells in Intestinal Barrier and Mucosal Immunity. J. Inflamm. Res..

[153] Liang L., Liu L., Zhou W., Yang C., Mai G., Li H., Chen Y. (2022). Gut microbiota-derived butyrate regulates gut mucus barrier repair by activating the macrophage/WNT/ERK signaling pathway. Clin. Sci..

[154] Rochette L., Lorin J., Zeller M., Guilland J.C., Lorgis L., Cottin Y., Vergely C. (2013). Nitric oxide synthase inhibition and oxidative stress in cardiovascular diseases: Possible therapeutic targets?. Pharmacol. Ther..

[155] Singh V., Kaur R., Kumari P., Pasricha C., Singh R. (2023). ICAM-1 and VCAM-1: Gatekeepers in various inflammatory and cardiovascular disorders. Clin. Chim. Acta.

[156] Gearing A.J., Hemingway I., Pigott R., Hughes J., Rees A.J., Cashman S.J. (1992). Soluble forms of vascular adhesion molecules, E-selectin, ICAM-1, and VCAM-1: Pathological significance. Ann. N. Y. Acad. Sci..

[157] Radi Z.A., Kehrli M.E., Ackermann M.R. (2001). Cell adhesion molecules, leukocyte trafficking, and strategies to reduce leukocyte infiltration. J. Vet. Intern. Med..

[158] Zhang H., Wang Y., Qu M., Li W., Wu D., Cata J.P., Miao C. (2023). Neutrophil, neutrophil extracellular traps and endothelial cell dysfunction in sepsis. Clin. Transl. Med..

[159] Li H., Horke S., Förstermann U. (2014). Vascular oxidative stress, nitric oxide and atherosclerosis. Atherosclerosis.

[160] Silva Y.P., Bernardi A., Frozza R.L. (2020). The Role of Short-Chain Fatty Acids From Gut Microbiota in Gut-Brain Communication. Front. Endocrinol..

[161] Yang J., Wu Z., Long Q., Huang J., Hong T., Liu W., Lin J. (2020). Insights Into Immunothrombosis: The Interplay Among Neutrophil Extracellular Trap, von Willebrand Factor, and ADAMTS13. Front. Immunol..

[162] Chen Z., Zhang H., Qu M., Nan K., Cao H., Cata J.P., Chen W., Miao C. (2021). Review: The Emerging Role of Neutrophil Extracellular Traps in Sepsis and Sepsis-Associated Thrombosis. Front. Cell. Infect. Microbiol..

[163] Meyers S., Crescente M., Verhamme P., Martinod K. (2022). *Staphylococcus aureus* and Neutrophil Extracellular Traps: The Master Manipulator Meets Its Match in Immunothrombosis. Arterioscler. Thromb. Vasc. Biol..

[164] Fernández-Pérez M.P., Águila S., Reguilón-Gallego L., de Los Reyes-García A.M., Miñano A., Bravo-Pérez C., de la Morena M.E., Corral J., García-Barberá N., Gómez-Verdú J.M. (2021). Neutrophil extracellular traps and von Willebrand factor are allies that negatively influence COVID-19 outcomes. Clin. Transl. Med..

[165] Janaszak-Jasiecka A., Płoska A., Wierońska J.M., Dobrucki L.W., Kalinowski L. (2023). Endothelial dysfunction due to eNOS uncoupling: Molecular mechanisms as potential therapeutic targets. Cell. Mol. Biol. Lett..

[166] Schulz E., Jansen T., Wenzel P., Daiber A., Münzel T. (2008). Nitric oxide, tetrahydrobiopterin, oxidative stress, and endothelial dysfunction in hypertension. Antioxid. Redox Signal..

[167] Beltran-Velasco A.I., Clemente-Suárez V.J. (2025). Impact of Peripheral Inflammation on Blood-Brain Barrier Dysfunction and Its Role in Neurodegenerative Diseases. Int. J. Mol. Sci..

[168] Ioannou M., Borkent J., Severance E.G., Yolken R.H., Fasano A., Sommer I.E.C., Haarman B.C.M. (2025). Biomarkers of intestinal permeability in major psychiatric disorders: Distinct biological roles call for a more nuanced application. Prog. Neuropsychopharmacol. Biol. Psychiatry.

[169] Liebner S., Dijkhuizen R.M., Reiss Y., Plate K.H., Agalliu D., Constantin G. (2018). Functional morphology of the blood-brain barrier in health and disease. Acta Neuropathol..

[170] Kadry H., Noorani B., Cucullo L. (2020). A blood-brain barrier overview on structure, function, impairment, and biomarkers of integrity. Fluids Barriers CNS.

[171] Varatharaj A., Galea I. (2017). The blood-brain barrier in systemic inflammation. Brain Behav. Immun..

[172] Zhao Y., Gan L., Ren L., Lin Y., Ma C., Lin X. (2022). Factors influencing the blood-brain barrier permeability. Brain Res..

[173] Bao X., Wu J., Xie Y., Kim S., Michelhaugh S., Jiang J., Mittal S., Sanai N., Li J. (2020). Protein Expression and Functional Relevance of Efflux and Uptake Drug Transporters at the Blood-Brain Barrier of Human Brain and Glioblastoma. Clin. Pharmacol. Ther..

[174] Galea I. (2021). The blood-brain barrier in systemic infection and inflammation. Cell. Mol. Immunol..

[175] Li Y., Liu B., Zhao T., Quan X., Han Y., Cheng Y., Chen Y., Shen X., Zheng Y., Zhao Y. (2023). Comparative study of extracellular vesicles derived from mesenchymal stem cells and brain endothelial cells attenuating blood-brain barrier permeability via regulating Caveolin-1-dependent ZO-1 and Claudin-5 endocytosis in acute ischemic stroke. J. Nanobiotechnol..

[176] Linville R.M., DeStefano J.G., Sklar M.B., Xu Z., Farrell A.M., Bogorad M.I., Chu C., Walczak P., Cheng L., Mahairaki V. (2019). Human iPSC-derived blood-brain barrier microvessels: Validation of barrier function and endothelial cell behavior. Biomaterials.

[177] Tanaka M., Szatmári I., Vécsei L. (2025). Quinoline Quest: Kynurenic Acid Strategies for Next-Generation Therapeutics via Rational Drug Design. Pharmaceuticals.

[178] Janigro D., Bailey D.M., Lehmann S., Badaut J., O’Flynn R., Hirtz C., Marchi N. (2020). Peripheral Blood and Salivary Biomarkers of Blood-Brain Barrier Permeability and Neuronal Damage: Clinical and Applied Concepts. Front. Neurol..

[179] Kraynak T.E., Marsland A.L., Wager T.D., Gianaros P.J. (2018). Functional neuroanatomy of peripheral inflammatory physiology: A meta-analysis of human neuroimaging studies. Neurosci. Biobehav. Rev..

[180] Tanaka M. (2026). Neurogenesis and Neuroinflammation in Dialogue: Mapping Gaps, Modulating Microglia, Rewiring Aging. Cells.

[181] Candelario-Jalil E., Dijkhuizen R.M., Magnus T. (2022). Neuroinflammation, Stroke, Blood-Brain Barrier Dysfunction, and Imaging Modalities. Stroke.

[182] Mayer M.G., Fischer T. (2024). Microglia at the blood brain barrier in health and disease. Front. Cell. Neurosci..

[183] Takata F., Nakagawa S., Matsumoto J., Dohgu S. (2021). Blood-Brain Barrier Dysfunction Amplifies the Development of Neuroinflammation: Understanding of Cellular Events in Brain Microvascular Endothelial Cells for Prevention and Treatment of BBB Dysfunction. Front. Cell. Neurosci..

[184] Belleau E.L., Treadway M.T., Pizzagalli D.A. (2019). The Impact of Stress and Major Depressive Disorder on Hippocampal and Medial Prefrontal Cortex Morphology. Biol. Psychiatry.

[185] Ronaldson P.T., Davis T.P. (2020). Regulation of blood-brain barrier integrity by microglia in health and disease: A therapeutic opportunity. J. Cereb. Blood Flow. Metab..

[186] de Lima E.P., Laurindo L.F., Catharin V.C.S., Direito R., Tanaka M., Jasmin Santos German I., Lamas C.B., Guiguer E.L., Araújo A.C., Fiorini A.M.R. (2025). Polyphenols, Alkaloids, and Terpenoids Against Neurodegeneration: Evaluating the Neuroprotective Effects of Phytocompounds Through a Comprehensive Review of the Current Evidence. Metabolites.

[187] Barbalho S.M., Leme Boaro B., da Silva Camarinha Oliveira J., Patočka J., Barbalho Lamas C., Tanaka M., Laurindo L.F. (2025). Molecular Mechanisms Underlying Neuroinflammation Intervention with Medicinal Plants: A Critical and Narrative Review of the Current Literature. Pharmaceuticals.

[188] Hare B.D., Duman R.S. (2020). Prefrontal cortex circuits in depression and anxiety: Contribution of discrete neuronal populations and target regions. Mol. Psychiatry.

[189] Pihelgas S., Ehala-Aleksejev K., Kutti M.L., Kuldjärv R., Kazantseva J. (2025). Impact of fresh and fermented vegetable consumption on gut microbiota and body composition: Insights from diverse data analysis approaches. Front. Nutr..

[190] Moon H.J., Oh S.H., Park K.B., Cha Y.S. (2023). Kimchi and *Leuconostoc mesenteroides* DRC 1506 Alleviate Dextran Sulfate Sodium (DSS)-Induced Colitis via Attenuating Inflammatory Responses. Foods.

[191] Wei L., Marco M.L. (2025). The fermented cabbage metabolome and its protection against cytokine-induced intestinal barrier disruption of Caco-2 monolayers. Appl. Environ. Microbiol..

[192] Kennedy P.J., Cryan J.F., Dinan T.G., Clarke G. (2017). Kynurenine pathway metabolism and the microbiota-gut-brain axis. Neuropharmacology.

[193] Figueiredo Godoy A.C., Frota F.F., Araújo L.P., Valenti V.E., Pereira E., Detregiachi C.R.P., Galhardi C.M., Caracio F.C., Haber R.S.A., Fornari Laurindo L. (2025). Neuroinflammation and Natural Antidepressants: Balancing Fire with Flora. Biomedicines.

[194] Shigetomi S., Fujimoto N., Hirano K., Matsuda T., Kato C., Nishimura N., Hino S. (2026). Interactions between Dietary Protein and Vegetable Fiber via the Gut Microbiota Are Associated with Cecal Fermentation Profiles and IgA Responses in Rats. J. Nutr..

[195] Wang Y., Cai X., Ma Y., Yang Y., Pan C.W., Zhu X., Ke C. (2024). Metabolomics on depression: A comparison of clinical and animal research. J. Affect. Disord..

[196] Jansen R., Milaneschi Y., Schranner D., Kastenmuller G., Arnold M., Han X., Dunlop B.W., Rush A.J., Kaddurah-Daouk R., Penninx B. (2024). The metabolome-wide signature of major depressive disorder. Mol. Psychiatry.

[197] Zeng L., Qian Y., Cui X., Zhao J., Ning Z., Cha J., Wang K., Ge C., Jia J., Dou T. (2025). Immunomodulatory role of gut microbial metabolites: Mechanistic insights and therapeutic frontiers. Front. Microbiol..

[198] Gasaly N., de Vos P., Hermoso M.A. (2021). Impact of Bacterial Metabolites on Gut Barrier Function and Host Immunity: A Focus on Bacterial Metabolism and Its Relevance for Intestinal Inflammation. Front. Immunol..

[199] Mann E.R., Lam Y.K., Uhlig H.H. (2024). Short-chain fatty acids: Linking diet, the microbiome and immunity. Nat. Rev. Immunol..

[200] Gill P.A., van Zelm M.C., Muir J.G., Gibson P.R. (2018). Review article: Short chain fatty acids as potential therapeutic agents in human gastrointestinal and inflammatory disorders. Aliment. Pharmacol. Ther..

[201] van Trijp M.P.H., Rios-Morales M., Witteman B., Abegaz F., Gerding A., An R., Koehorst M., Evers B., van Dongen K.C.V., Zoetendal E.G. (2024). Intraintestinal fermentation of fructo- and galacto-oligosaccharides and the fate of short-chain fatty acids in humans. iScience.

[202] Kirschner S.K., Engelen M.P., Haas P., Bischoff S.C., Deutz N.E. (2025). Short-chain fatty acid kinetics and concentrations are higher after inulin supplementation in young and older adults: A randomized trial. Am. J. Clin. Nutr..

[203] Wong J.M., de Souza R., Kendall C.W., Emam A., Jenkins D.J. (2006). Colonic health: Fermentation and short chain fatty acids. J. Clin. Gastroenterol..

[204] Louis P., Flint H.J. (2017). Formation of propionate and butyrate by the human colonic microbiota. Environ. Microbiol..

[205] Boets E., Deroover L., Houben E., Vermeulen K., Gomand S.V., Delcour J.A., Verbeke K. (2015). Quantification of in Vivo Colonic Short Chain Fatty Acid Production from Inulin. Nutrients.

[206] Du Y., He C., An Y., Huang Y., Zhang H., Fu W., Wang M., Shan Z., Xie J., Yang Y. (2024). The Role of Short Chain Fatty Acids in Inflammation and Body Health. Int. J. Mol. Sci..

[207] Sanchez-Ledesma L.M., Rodríguez-Victoria J.A., Ramírez-Malule H. (2024). Effect of fermentation time, pH, and their interaction on the production of volatile fatty acids from cassava wastewater. Water.

[208] e Silva A.P.R., Pantoja G.V., Crispino A.C.S., Braga Y.P., Lima M.F.T., Silva L.F. (2023). Tucupi: A review of uses, physical-chemical, nutritional properties and technologies Tucupi: Uma revisão dos usos, propriedades físico-químicas, nutricionais e tecnologias. Braz. J. Dev..

[209] Halake N.H., Chinthapalli B. (2020). Fermentation of traditional African Cassava based foods: Microorganisms role in nutritional and safety value. J. Exp. Agric. Int..

[210] Olaniyan S.A., Hussein J.B., Oke M.O., Akinwande B.A., Workneh T.S., Ayodele M., Adeyemi I.A. (2025). Integrated bioprocessing of cassava residues for enzymatic starch recovery, citric acid production, and effluent detoxification. Sci. Rep..

[211] Zhang D., Jian Y.P., Zhang Y.N., Li Y., Gu L.T., Sun H.H., Liu M.D., Zhou H.L., Wang Y.S., Xu Z.X. (2023). Short-chain fatty acids in diseases. Cell Commun. Signal..

[212] Facchin S., Bertin L., Bonazzi E., Lorenzon G., De Barba C., Barberio B., Zingone F., Maniero D., Scarpa M., Ruffolo C. (2024). Short-Chain Fatty Acids and Human Health: From Metabolic Pathways to Current Therapeutic Implications. Life.

[213] Liu P., Wang Y., Yang G., Zhang Q., Meng L., Xin Y., Jiang X. (2021). The role of short-chain fatty acids in intestinal barrier function, inflammation, oxidative stress, and colonic carcinogenesis. Pharmacol. Res..

[214] Wang L.Y., He L.H., Xu L.J., Li S.B. (2024). Short-chain fatty acids: Bridges between diet, gut microbiota, and health. J. Gastroenterol. Hepatol..

[215] Tao Z., Wang Y. (2025). The health benefits of dietary short-chain fatty acids in metabolic diseases. Crit. Rev. Food Sci. Nutr..

[216] Li M., van Esch B., Wagenaar G.T.M., Garssen J., Folkerts G., Henricks P.A.J. (2018). Pro- and anti-inflammatory effects of short chain fatty acids on immune and endothelial cells. Eur. J. Pharmacol..

[217] Weng C.C., Suarez C., Cheang S.E., Couture G., Goodson M.L., Barboza M., Kalanetra K.M., Masarweh C.F., Mills D.A., Raybould H.E. (2024). Quantifying Gut Microbial Short-Chain Fatty Acids and Their Isotopomers in Mechanistic Studies Using a Rapid, Readily Expandable LC-MS Platform. Anal. Chem..

[218] Li M., van Esch B., Henricks P.A.J., Folkerts G., Garssen J. (2018). The Anti-inflammatory Effects of Short Chain Fatty Acids on Lipopolysaccharide- or Tumor Necrosis Factor α-Stimulated Endothelial Cells via Activation of GPR41/43 and Inhibition of HDACs. Front. Pharmacol..

[219] Wang W., Dernst A., Martin B., Lorenzi L., Cadefau-Fabregat M., Phulphagar K., Wagener A., Budden C., Stair N., Wagner T. (2024). Butyrate and propionate are microbial danger signals that activate the NLRP3 inflammasome in human macrophages upon TLR stimulation. Cell Rep..

[220] Fock E., Parnova R. (2023). Mechanisms of Blood-Brain Barrier Protection by Microbiota-Derived Short-Chain Fatty Acids. Cells.

[221] Cheng J., Hu H., Ju Y., Liu J., Wang M., Liu B., Zhang Y. (2024). Gut microbiota-derived short-chain fatty acids and depression: Deep insight into biological mechanisms and potential applications. Gen. Psychiatr..

[222] Zhou Y., Chen Y., He H., Peng M., Zeng M., Sun H. (2023). The role of the indoles in microbiota-gut-brain axis and potential therapeutic targets: A focus on human neurological and neuropsychiatric diseases. Neuropharmacology.

[223] Bessede A., Gargaro M., Pallotta M.T., Matino D., Servillo G., Brunacci C., Bicciato S., Mazza E.M., Macchiarulo A., Vacca C. (2014). Aryl hydrocarbon receptor control of a disease tolerance defence pathway. Nature.

[224] Gutiérrez-Vázquez C., Quintana F.J. (2018). Regulation of the Immune Response by the Aryl Hydrocarbon Receptor. Immunity.

[225] Agus A., Planchais J., Sokol H. (2018). Gut Microbiota Regulation of Tryptophan Metabolism in Health and Disease. Cell Host Microbe.

[226] Suneson K., Lindahl J., Chamli Hårsmar S., Söderberg G., Lindqvist D. (2021). Inflammatory Depression-Mechanisms and Non-Pharmacological Interventions. Int. J. Mol. Sci..

[227] MahmoudianDehkordi S., Bhattacharyya S., Brydges C.R., Jia W., Fiehn O., Rush A.J., Dunlop B.W., Kaddurah-Daouk R. (2022). Gut Microbiome-Linked Metabolites in the Pathobiology of Major Depression With or Without Anxiety—A Role for Bile Acids. Front. Neurosci..

[228] Zhao X., Zheng I., Huang W., Tang D., Zhao M., Hou R., Huang Y., Shi Y., Zhu W., Wang S. (2025). Research Progress on the Mechanism of Bile Acids and Their Receptors in Depression. Int. J. Mol. Sci..

[229] Chávez-Talavera O., Tailleux A., Lefebvre P., Staels B. (2017). Bile Acid Control of Metabolism and Inflammation in Obesity, Type 2 Diabetes, Dyslipidemia, and Nonalcoholic Fatty Liver Disease. Gastroenterology.

[230] Guo C., Xie S., Chi Z., Zhang J., Liu Y., Zhang L., Zheng M., Zhang X., Xia D., Ke Y. (2016). Bile Acids Control Inflammation and Metabolic Disorder through Inhibition of NLRP3 Inflammasome. Immunity.

[231] Fiaschini N., Mancuso M., Tanori M., Colantoni E., Vitali R., Diretto G., Lorenzo Rebenaque L., Stronati L., Negroni A. (2022). Liver Steatosis and Steatohepatitis Alter Bile Acid Receptors in Brain and Induce Neuroinflammation: A Contribution of Circulating Bile Acids and Blood-Brain Barrier. Int. J. Mol. Sci..

[232] Chakrabarti S., Jahandideh F., Wu J. (2014). Food-derived bioactive peptides on inflammation and oxidative stress. BioMed Res. Int..

[233] Aluko R.E. (2015). Antihypertensive peptides from food proteins. Annu. Rev. Food Sci. Technol..

[234] Okagu I.U., Ezeorba T.P.C., Aham E.C., Aguchem R.N., Nechi R.N. (2022). Recent findings on the cellular and molecular mechanisms of action of novel food-derived antihypertensive peptides. Food Chem..

[235] Chakrabarti S., Wu J. (2016). Bioactive peptides on endothelial function. Food Sci. Hum. Wellness.

[236] Laiño J., Villena J., Kanmani P., Kitazawa H. (2016). Immunoregulatory Effects Triggered by Lactic Acid Bacteria Exopolysaccharides: New Insights into Molecular Interactions with Host Cells. Microorganisms.

[237] Akkerman R., Oerlemans M.M.P., Ferrari M., Fernández-Lainez C., Walvoort M.T.C., de Vos P. (2025). Exopolysaccharides from *Bifidobacterium longum* subsp. *infantis* and *Bifidobacterium adolescentis* modulate Toll-like receptor signaling. Carbohydr. Polym..

[238] Shukla A., Tangney M. (2025). Bacterial exopolysaccharides and live biotherapeutics: Orchestrating immune modulation and therapeutic potential in the gut microbiome era. Biomed. Pharmacother..

[239] Grifka-Walk H.M., Jenkins B.R., Kominsky D.J. (2021). Amino Acid Trp: The Far Out Impacts of Host and Commensal Tryptophan Metabolism. Front. Immunol..

[240] Gao J., Xu K., Liu H., Liu G., Bai M., Peng C., Li T., Yin Y. (2018). Impact of the Gut Microbiota on Intestinal Immunity Mediated by Tryptophan Metabolism. Front. Cell. Infect. Microbiol..

[241] Tanaka M., Vécsei L. (2025). From Microbial Switches to Metabolic Sensors: Rewiring the Gut-Brain Kynurenine Circuit. Biomedicines.

[242] Gao K., Mu C.L., Farzi A., Zhu W.Y. (2020). Tryptophan Metabolism: A Link Between the Gut Microbiota and Brain. Adv. Nutr..

[243] Juhász L., Galla Z., Tanaka M., Vécsei L. (2026). Receptor-Mitochondria Crosstalk in the Kynurenine Metabolic Pathway: Integrating Metabolomics and Clinical Mass Spectrometry. Antioxidants.

[244] Szabó Á., Galla Z., Spekker E., Martos D., Szűcs M., Fejes-Szabó A., Fehér Á., Takeda K., Ozaki K., Inoue H. (2025). Behavioral Balance in Tryptophan Turmoil: Regional Metabolic Rewiring in Kynurenine Aminotransferase II Knockout Mice. Cells.

[245] Sinha A.K., Laursen M.F., Brinck J.E., Rybtke M.L., Hjørne A.P., Procházková N., Pedersen M., Roager H.M., Licht T.R. (2024). Dietary fibre directs microbial tryptophan metabolism via metabolic interactions in the gut microbiota. Nat. Microbiol..

[246] Cellini B., Zelante T., Dindo M., Bellet M.M., Renga G., Romani L., Costantini C. (2020). Pyridoxal 5′-Phosphate-Dependent Enzymes at the Crossroads of Host-Microbe Tryptophan Metabolism. Int. J. Mol. Sci..

[247] Bosi A., Banfi D., Bistoletti M., Giaroni C., Baj A. (2020). Tryptophan Metabolites Along the Microbiota-Gut-Brain Axis: An Interkingdom Communication System Influencing the Gut in Health and Disease. Int. J. Tryptophan Res..

[248] Hou Y., Li J., Ying S. (2023). Tryptophan Metabolism and Gut Microbiota: A Novel Regulatory Axis Integrating the Microbiome, Immunity, and Cancer. Metabolites.

[249] Gupta S.K., Vyavahare S., Duchesne Blanes I.L., Berger F., Isales C., Fulzele S. (2023). Microbiota-derived tryptophan metabolism: Impacts on health, aging, and disease. Exp. Gerontol..

[250] Martos D., Lőrinczi B., Szatmári I., Vécsei L., Tanaka M. (2025). Decoupling Behavioral Domains via Kynurenic Acid Analog Optimization: Implications for Schizophrenia and Parkinson’s Disease Therapeutics. Cells.

[251] Juhász L., Spisák K., Szolnoki B.Z., Nászai A., Szabó Á., Rutai A., Tallósy S.P., Szabó A., Toldi J., Tanaka M. (2025). The Power Struggle: Kynurenine Pathway Enzyme Knockouts and Brain Mitochondrial Respiration. J. Neurochem..

[252] Costantini C., Bellet M.M., Renga G., Stincardini C., Borghi M., Pariano M., Cellini B., Keller N., Romani L., Zelante T. (2020). Tryptophan Co-Metabolism at the Host-Pathogen Interface. Front. Immunol..

[253] Hyland N.P., Cavanaugh C.R., Hornby P.J. (2022). Emerging effects of tryptophan pathway metabolites and intestinal microbiota on metabolism and intestinal function. Amino Acids.

[254] Li S. (2023). Modulation of immunity by tryptophan microbial metabolites. Front. Nutr..

[255] Shaw C., Hess M., Weimer B.C. (2023). Microbial-Derived Tryptophan Metabolites and Their Role in Neurological Disease: Anthranilic Acid and Anthranilic Acid Derivatives. Microorganisms.

[256] Huang Z., Schoones T., Wells J.M., Fogliano V., Capuano E. (2021). Substrate-Driven Differences in Tryptophan Catabolism by Gut Microbiota and Aryl Hydrocarbon Receptor Activation. Mol. Nutr. Food Res..

[257] Stanimirov B., Đanić M., Pavlović N., Zaklan D., Lazarević S., Mikov M., Stankov K. (2025). Gut-Brain Axis and Bile Acid Signaling: Linking Microbial Metabolism to Brain Function and Metabolic Regulation. Int. J. Mol. Sci..

[258] Cai J., Rimal B., Jiang C., Chiang J.Y.L., Patterson A.D. (2022). Bile acid metabolism and signaling, the microbiota, and metabolic disease. Pharmacol. Ther..

[259] Monteiro-Cardoso V.F., Corlianò M., Singaraja R.R. (2021). Bile Acids: A Communication Channel in the Gut-Brain Axis. Neuromol. Med..

[260] Pathak P., Xie C., Nichols R.G., Ferrell J.M., Boehme S., Krausz K.W., Patterson A.D., Gonzalez F.J., Chiang J.Y.L. (2018). Intestine farnesoid X receptor agonist and the gut microbiota activate G-protein bile acid receptor-1 signaling to improve metabolism. Hepatology.

[261] Ferrell J.M., Chiang J.Y.L. (2021). Bile acid receptors and signaling crosstalk in the liver, gut and brain. Liver Res..

[262] Wegner K., Just S., Gau L., Mueller H., Gérard P., Lepage P., Clavel T., Rohn S. (2017). Rapid analysis of bile acids in different biological matrices using LC-ESI-MS/MS for the investigation of bile acid transformation by mammalian gut bacteria. Anal. Bioanal. Chem..

[263] Yan W., Zhang K., Guo J., Xu L. (2025). Bile acid-mediated gut-liver axis crosstalk: The role of nuclear receptor signaling in dynamic regulation of inflammatory networks. Front. Immunol..

[264] He Y., Shaoyong W., Chen Y., Li M., Gan Y., Sun L., Liu Y., Wang Y., Jin M. (2026). The functions of gut microbiota-mediated bile acid metabolism in intestinal immunity. J. Adv. Res..

[265] Tyagi A., Kumar V. (2025). The gut microbiota-bile acid axis: A crucial regulator of immune function and metabolic health. World J. Microbiol. Biotechnol..

[266] Chiang J.Y., Pathak P., Liu H., Donepudi A., Ferrell J., Boehme S. (2017). Intestinal Farnesoid X Receptor and Takeda G Protein Couple Receptor 5 Signaling in Metabolic Regulation. Dig. Dis..

[267] Chiang J.Y.L. (2017). Bile acid metabolism and signaling in liver disease and therapy. Liver Res..

[268] Reiter S., Dunkel A., Dawid C., Hofmann T. (2021). Targeted LC-MS/MS Profiling of Bile Acids in Various Animal Tissues. J. Agric. Food Chem..

[269] Gao T., Hu S., Xu W., Wang Z., Guo T., Chen F., Ma Y., Zhu L., Chen F., Wang X. (2024). Targeted LC-MS/MS profiling of bile acids reveals primary/secondary bile acid ratio as a novel biomarker for necrotizing enterocolitis. Anal. Bioanal. Chem..

[270] Ramos-Garcia V., Ten-Doménech I., Vento M., Bullich-Vilarrubias C., Romaní-Pérez M., Sanz Y., Nobili A., Falcone M., Di Stefano M., Quintás G. (2023). Fast profiling of primary, secondary, conjugated, and sulfated bile acids in human urine and murine feces samples. Anal. Bioanal. Chem..

[271] Künili İ.E., Akdeniz V., Akpınar A., Budak Ş., Curiel J.A., Guzel M., Karagözlü C., Berkel Kasikci M., Caruana G.P.M., Starowicz M. (2025). Bioactive compounds in fermented foods: A systematic narrative review. Front. Nutr..

[272] Diez-Ozaeta I., Astiazaran O.J. (2022). Fermented foods: An update on evidence-based health benefits and future perspectives. Food Res. Int..

[273] Yılmaz C., Gökmen V. (2020). Neuroactive compounds in foods: Occurrence, mechanism and potential health effects. Food Res. Int..

[274] Han M., Dong Y., Wang S., Huang X., Bai C., Gai Z. (2024). Regulation of gut microbiota and serum neurotransmitters in mice by *Streptococcus thermophilus* GA8- and *Lacticaseibacillus rhamnosus* HAO9-fermented milk containing high levels of gamma-aminobutyric acid. J. Sci. Food Agric..

[275] Porras-García E., Fernández-Espada Calderón I., Gavala-González J., Fernández-García J.C. (2023). Potential neuroprotective effects of fermented foods and beverages in old age: A systematic review. Front. Nutr..

[276] Li K.J., Burton-Pimentel K.J., Brouwer-Brolsma E.M., Blaser C., Badertscher R., Pimentel G., Portmann R., Feskens E.J.M., Vergères G. (2023). Identifying Plasma and Urinary Biomarkers of Fermented Food Intake and Their Associations with Cardiometabolic Health in a Dutch Observational Cohort. J. Agric. Food Chem..

[277] Parada Venegas D., De la Fuente M.K., Landskron G., González M.J., Quera R., Dijkstra G., Harmsen H.J.M., Faber K.N., Hermoso M.A. (2019). Short Chain Fatty Acids (SCFAs)-Mediated Gut Epithelial and Immune Regulation and Its Relevance for Inflammatory Bowel Diseases. Front. Immunol..

[278] Cheng J., Wu Q., Sun R., Li W., Wang Z., Zhou M., Yang T., Wang J., Lyu Y., Yue C. (2024). Protective effects of a probiotic-fermented germinated grain complex on neurotransmitters and sleep quality in sleep-deprived mice. Front. Microbiol..

[279] Li R., Shan S., Xu Y., Xiong J., Cheng G. (2025). Identification of bioaccessible and neuroprotective peptides from fermented casein hydrolysate. J. Dairy Sci..

[280] Rizwan D., Masoodi F., Wani S.M., Mir S.A. (2023). Bioactive peptides from fermented foods and their relevance in COVID-19 mitigation. Food Prod. Process. Nutr..

[281] Guo Q., Chen P., Chen X. (2023). Bioactive peptides derived from fermented foods: Preparation and biological activities. J. Funct. Foods.

[282] Peighambardoust S.H., Karami Z., Pateiro M., Lorenzo J.M. (2021). A Review on Health-Promoting, Biological, and Functional Aspects of Bioactive Peptides in Food Applications. Biomolecules.

[283] Chai K.F., Voo A.Y.H., Chen W.N. (2020). Bioactive peptides from food fermentation: A comprehensive review of their sources, bioactivities, applications, and future development. Compr. Rev. Food Sci. Food Saf..

[284] Chaudhary A., Bhalla S., Patiyal S., Raghava G.P.S., Sahni G. (2021). FermFooDb: A database of bioactive peptides derived from fermented foods. Heliyon.

[285] Chourasia R., Chiring Phukon L., Abedin M.M., Padhi S., Singh S.P., Rai A.K. (2023). Bioactive peptides in fermented foods and their application: A critical review. Syst. Microbiol. Biomanuf..

[286] Xia W., Han J., Zhu S., Wang Y., Zhang W., Wu Z. (2023). Structural elucidation of the exopolysaccharide from *Streptococcus thermophilus* XJ53 and the effect of its molecular weight on immune activity. Int. J. Biol. Macromol..

[287] Wang N., Jia G., Wang C., Chen M., Xie F., Nepovinnykh N., Goff H.D., Guo Q. (2020). Structural characterisation and immunomodulatory activity of exopolysaccharides from liquid fermentation of *Monascus purpureus* (Hong Qu). Food Hydrocoll..

[288] Xiao M., Tang L., Fu X., Ren X., Bi J., Wang J., Li D., Kong Q., Mou H., Zhu C. (2024). Preparation, structural characterization and rheological properties of a novel fucose-containing exopolysaccharide from *Clavibacter michiganensis*. Food Hydrocoll..

[289] Bhattacharjee M.J., Bala A., Khan M.R., Mukherjee A.K. (2025). Functional impact of bioactive peptides derived from fermented foods on diverse human populations. Food Chem..

[290] Martin M., Deussen A. (2019). Effects of natural peptides from food proteins on angiotensin converting enzyme activity and hypertension. Crit. Rev. Food Sci. Nutr..

[291] Xue L., Yin R., Howell K., Zhang P. (2021). Activity and bioavailability of food protein-derived angiotensin-I-converting enzyme-inhibitory peptides. Compr. Rev. Food Sci. Food Saf..

[292] Xiang L., Qiu Z., Zhao R., Zheng Z., Qiao X. (2023). Advancement and prospects of production, transport, functional activity and structure-activity relationship of food-derived angiotensin converting enzyme (ACE) inhibitory peptides. Crit. Rev. Food Sci. Nutr..

[293] Olalere O.A., Yap P.-G., Gan C.-Y. (2023). Comprehensive review on some food-derived bioactive peptides with anti-hypertension therapeutic potential for angiotensin-converting enzyme (ACE) inhibition. J. Proteins Proteom..

[294] Tonolo F., Moretto L., Grinzato A., Fiorese F., Folda A., Scalcon V., Ferro S., Arrigoni G., Bellamio M., Feller E. (2020). Fermented Soy-Derived Bioactive Peptides Selected by a Molecular Docking Approach Show Antioxidant Properties Involving the Keap1/Nrf2 Pathway. Antioxidants.

[295] Lin H., Zhao J., Xie Y., Tang J., Wang Q., Zhao J., Xu M., Liu P. (2024). Identification and molecular mechanisms of novel antioxidant peptides from fermented broad bean paste: A combined in silico and in vitro study. Food Chem..

[296] Wan Y.J., Hong T., Shi H.F., Yin J.Y., Koev T., Nie S.P., Gilbert R.G., Xie M.Y. (2021). Probiotic fermentation modifies the structures of pectic polysaccharides from carrot pulp. Carbohydr. Polym..

[297] Mussbacher M., Salzmann M., Brostjan C., Hoesel B., Schoergenhofer C., Datler H., Hohensinner P., Basílio J., Petzelbauer P., Assinger A. (2019). Cell Type-Specific Roles of NF-κB Linking Inflammation and Thrombosis. Front. Immunol..

[298] Hassamal S. (2023). Chronic stress, neuroinflammation, and depression: An overview of pathophysiological mechanisms and emerging anti-inflammatories. Front. Psychiatry.

[299] Wang R., Xiao F., Peng W., Yuan X. (2025). Molecular regulatory mechanisms of depression-related thrombosis risk. J. Med. Biochem..

[300] Koudouovoh-Tripp P., Hüfner K., Egeter J., Kandler C., Giesinger J.M., Sopper S., Humpel C., Sperner-Unterweger B. (2021). Stress Enhances Proinflammatory Platelet Activity: The Impact of Acute and Chronic Mental Stress. J. Neuroimmune Pharmacol..

[301] van Dooren F.E., Schram M.T., Schalkwijk C.G., Stehouwer C.D., Henry R.M., Dagnelie P.C., Schaper N.C., van der Kallen C.J., Koster A., Sep S.J. (2016). Associations of low grade inflammation and endothelial dysfunction with depression—The Maastricht Study. Brain Behav. Immun..

[302] Waclawovsky A.J., de Brito E., Smith L., Vancampfort D., da Silva A.M.V., Schuch F.B. (2021). Endothelial dysfunction in people with depressive disorders: A systematic review and meta-analysis. J. Psychiatr. Res..

[303] Nicolai L., Kaiser R., Stark K. (2023). Thromboinflammation in long COVID-the elusive key to postinfection sequelae?. J. Thromb. Haemost..

[304] Fekete M., Lehoczki A., Szappanos Á., Toth A., Mahdi M., Sótonyi P., Benyó Z., Yabluchanskiy A., Tarantini S., Ungvari Z. (2025). Cerebromicrovascular mechanisms contributing to long COVID: Implications for neurocognitive health. Geroscience.

[305] Sherif Z.A., Gomez C.R., Connors T.J., Henrich T.J., Reeves W.B. (2023). Pathogenic mechanisms of post-acute sequelae of SARS-CoV-2 infection (PASC). eLife.

[306] Hally K.E., Parker O.M., Brunton-O’Sullivan M.M., Harding S.A., Larsen P.D. (2021). Linking Neutrophil Extracellular Traps and Platelet Activation: A Composite Biomarker Score for Predicting Outcomes after Acute Myocardial Infarction. Thromb. Haemost..

[307] Waclawovsky A.J., Dos Santos E.B., de Oliveira A.A.R., Stubbs B., Schuch F.B. (2025). Plasma biomarkers of endothelial function in people with depressive disorder: A systematic review and meta-analysis. J. Affect. Disord..

[308] Lopez-Vilchez I., Diaz-Ricart M., Navarro V., Torramade S., Zamorano-Leon J., Lopez-Farre A., Galan A.M., Gasto C., Escolar G. (2016). Endothelial damage in major depression patients is modulated by SSRI treatment, as demonstrated by circulating biomarkers and an in vitro cell model. Transl. Psychiatry.

[309] Delialis D., Mavraganis G., Dimoula A., Patras R., Dimopoulou A.M., Sianis A., Ajdini E., Maneta E., Kokras N., Stamatelopoulos K. (2022). A systematic review and meta-analysis on the effect of selective serotonin reuptake inhibitors on endothelial function. J. Affect. Disord..

[310] Dawood T., Barton D.A., Lambert E.A., Eikelis N., Lambert G.W. (2016). Examining Endothelial Function and Platelet Reactivity in Patients with Depression before and after SSRI Therapy. Front. Psychiatry.

[311] Brambilla M., Josefsson E.C., Ramstrom S., Di Minno A., Di Minno M.N.D., Gangatirkar P., Moujalled D., Becchetti A., Lordkipanidzé M., Camera M. (2025). Biomarkers of in vivo platelet activation in coronary artery disease: A systematic review and meta-analysis: Communication from the SSC of the ISTH. J. Thromb. Haemost..

[312] Kannan M., Ahmad F., Saxena R. (2019). Platelet activation markers in evaluation of thrombotic risk factors in various clinical settings. Blood Rev..

[313] Shyamkrishnan R., Saharia G.K., Patra S., Bandyopadhyay D., Patro B.K. (2022). Flow cytometry based platelet activation markers and state of inflammation among subjects with type 2 diabetes with and without depression. Sci. Rep..

[314] Nemet I., Saha P.P., Gupta N., Zhu W., Romano K.A., Skye S.M., Cajka T., Mohan M.L., Li L., Wu Y. (2020). A Cardiovascular Disease-Linked Gut Microbial Metabolite Acts via Adrenergic Receptors. Cell.

[315] Zhu W., Gregory J.C., Org E., Buffa J.A., Gupta N., Wang Z., Li L., Fu X., Wu Y., Mehrabian M. (2016). Gut Microbial Metabolite TMAO Enhances Platelet Hyperreactivity and Thrombosis Risk. Cell.

[316] Ghatge M., Flora G.D., Nayak M.K., Chauhan A.K. (2024). Platelet Metabolic Profiling Reveals Glycolytic and 1-Carbon Metabolites Are Essential for GP VI-Stimulated Human Platelets-Brief Report. Arterioscler. Thromb. Vasc. Biol..

[317] Huynh K. (2020). Novel gut microbiota-derived metabolite promotes platelet thrombosis via adrenergic receptor signalling. Nat. Rev. Cardiol..

[318] Garg R. (2025). From gut to blood: How microbiome metabolites orchestrate platelet function. J. Hematol. Allied Sci..

[319] Ascher S., Reinhardt C. (2018). The gut microbiota: An emerging risk factor for cardiovascular and cerebrovascular disease. Eur. J. Immunol..

[320] Kračun D., Lopes L.R., Cifuentes-Pagano E., Pagano P.J. (2025). NADPH oxidases: Redox regulation of cell homeostasis and disease. Physiol. Rev..

[321] Folco E.J., Mawson T.L., Vromman A., Bernardes-Souza B., Franck G., Persson O., Nakamura M., Newton G., Luscinskas F.W., Libby P. (2018). Neutrophil Extracellular Traps Induce Endothelial Cell Activation and Tissue Factor Production Through Interleukin-1α and Cathepsin G. Arterioscler. Thromb. Vasc. Biol..

[322] Döring Y., Soehnlein O., Weber C. (2017). Neutrophil extracellular traps in atherosclerosis and atherothrombosis. Circ. Res..

[323] Azzouz D., Palaniyar N. (2024). How Do ROS Induce NETosis? Oxidative DNA Damage, DNA Repair, and Chromatin Decondensation. Biomolecules.

[324] Zambrano F., Uribe P., Schulz M., Hermosilla C., Taubert A., Sánchez R. (2025). Antioxidants as Modulators of NETosis: Mechanisms, Evidence, and Therapeutic Potential. Int. J. Mol. Sci..

[325] Pecchillo Cimmino T., Ammendola R., Cattaneo F., Esposito G. (2023). NOX Dependent ROS Generation and Cell Metabolism. Int. J. Mol. Sci..

[326] Duttaroy A.K. (2021). Role of Gut Microbiota and Their Metabolites on Atherosclerosis, Hypertension and Human Blood Platelet Function: A Review. Nutrients.

[327] Wang H., Kim S.J., Lei Y., Wang S., Wang H., Huang H., Zhang H., Tsung A. (2024). Neutrophil extracellular traps in homeostasis and disease. Signal Transduct. Target. Ther..

[328] González-Jiménez P., Méndez R., Latorre A., Mengot N., Piqueras M., Reyes S., Moscardó A., Alonso R., Amara-Elori I., Menéndez R. (2023). Endothelial Damage, Neutrophil Extracellular Traps and Platelet Activation in COVID-19 vs. Community-Acquired Pneumonia: A Case-Control Study. Int. J. Mol. Sci..

[329] Garabet L., Henriksson C.E., Lozano M.L., Ghanima W., Bussel J., Brodin E., Fernández-Pérez M.P., Martínez C., González-Conejero R., Mowinckel M.C. (2020). Markers of endothelial cell activation and neutrophil extracellular traps are elevated in immune thrombocytopenia but are not enhanced by thrombopoietin receptor agonists. Thromb. Res..

[330] Kasai S., Shimizu S., Tatara Y., Mimura J., Itoh K. (2020). Regulation of Nrf2 by Mitochondrial Reactive Oxygen Species in Physiology and Pathology. Biomolecules.

[331] He J., Zhang P., Shen L., Niu L., Tan Y., Chen L., Zhao Y., Bai L., Hao X., Li X. (2020). Short-Chain Fatty Acids and Their Association with Signalling Pathways in Inflammation, Glucose and Lipid Metabolism. Int. J. Mol. Sci..

[332] Bailey M.A., Thompson S.V., Mysonhimer A.R., Bennett J.N., Vanhie J.J., De Lisio M., Burd N.A., Khan N.A., Holscher H.D. (2023). Dietary fiber intake and fecal short-chain fatty acid concentrations are associated with lower plasma lipopolysaccharide-binding protein and inflammation. Am. J. Physiol. Gastrointest. Liver Physiol..

[333] Liu P., Liu Z., Wang J., Wang J., Gao M., Zhang Y., Yang C., Zhang A., Li G., Li X. (2024). Immunoregulatory role of the gut microbiota in inflammatory depression. Nat. Commun..

[334] Oliver A., Alkan Z., Stephensen C.B., Newman J.W., Kable M.E., Lemay D.G. (2024). Diet, Microbiome, and Inflammation Predictors of Fecal and Plasma Short-Chain Fatty Acids in Humans. J. Nutr..

[335] Gárate I., Garcia-Bueno B., Madrigal J.L., Caso J.R., Alou L., Gomez-Lus M.L., Micó J.A., Leza J.C. (2013). Stress-induced neuroinflammation: Role of the Toll-like receptor-4 pathway. Biol. Psychiatry.

[336] Janssen E., Köhler S., Geraets A.F.J., Stehouwer C.D.A., Schaper N.C., Sep S.J.S., Henry R.M.A., van der Kallen C.J.H., Schalkwijk C.G., Koster A. (2021). Low-grade inflammation and endothelial dysfunction predict four-year risk and course of depressive symptoms: The Maastricht study. Brain Behav. Immun..

[337] Collins T., Read M.A., Neish A.S., Whitley M.Z., Thanos D., Maniatis T. (1995). Transcriptional regulation of endothelial cell adhesion molecules: NF-κB and cytokine-inducible enhancers. FASEB J..

[338] Rolling C.C., Barrett T.J., Berger J.S. (2023). Platelet-monocyte aggregates: Molecular mediators of thromboinflammation. Front. Cardiovasc. Med..

[339] Kappelmayer J., Nagy B., Miszti-Blasius K., Hevessy Z., Setiadi H. (2004). The emerging value of P-selectin as a disease marker. Clin. Chem. Lab. Med..

[340] Brydon L., Magid K., Steptoe A. (2006). Platelets, coronary heart disease, and stress. Brain Behav. Immun..

[341] Zhu W., Wang Z., Tang W.H.W., Hazen S.L. (2017). Gut Microbe-Generated Trimethylamine N-Oxide From Dietary Choline Is Prothrombotic in Subjects. Circulation.

[342] Tanaka M., Tóth F., Polyák H., Szabó Á., Mándi Y., Vécsei L. (2021). Immune Influencers in Action: Metabolites and Enzymes of the Tryptophan-Kynurenine Metabolic Pathway. Biomedicines.

[343] Correia A.S., Vale N. (2022). Tryptophan Metabolism in Depression: A Narrative Review with a Focus on Serotonin and Kynurenine Pathways. Int. J. Mol. Sci..

[344] Badawy A.A., Guillemin G. (2019). The Plasma [Kynurenine]/[Tryptophan] Ratio and Indoleamine 2,3-Dioxygenase: Time for Appraisal. Int. J. Tryptophan Res..

[345] Hunt C., Macedo E.C.T., Suchting R., de Dios C., Cuellar Leal V.A., Soares J.C., Dantzer R., Teixeira A.L., Selvaraj S. (2020). Effect of immune activation on the kynurenine pathway and depression symptoms—A systematic review and meta-analysis. Neurosci. Biobehav. Rev..

[346] Haroon E., Welle J.R., Woolwine B.J., Goldsmith D.R., Baer W., Patel T., Felger J.C., Miller A.H. (2020). Associations among peripheral and central kynurenine pathway metabolites and inflammation in depression. Neuropsychopharmacology.

[347] Horvath S., Raj K. (2018). DNA methylation-based biomarkers and the epigenetic clock theory of ageing. Nat. Rev. Genet..

[348] Hillary R.F., Stevenson A.J., McCartney D.L., Campbell A., Walker R.M., Howard D.M., Ritchie C.W., Horvath S., Hayward C., McIntosh A.M. (2020). Epigenetic measures of ageing predict the prevalence and incidence of leading causes of death and disease burden. Clin. Epigenet..

[349] Protsenko E., Yang R., Nier B., Reus V., Hammamieh R., Rampersaud R., Wu G.W.Y., Hough C.M., Epel E., Prather A.A. (2021). GrimAge,” an epigenetic predictor of mortality, is accelerated in major depressive disorder. Transl. Psychiatry.

[350] Oblak L., van der Zaag J., Higgins-Chen A.T., Levine M.E., Boks M.P. (2021). A systematic review of biological, social and environmental factors associated with epigenetic clock acceleration. Ageing Res. Rev..

[351] Park Y.K., Lee J.H., Mah J.H. (2019). Occurrence and Reduction of Biogenic Amines in Kimchi and Korean Fermented Seafood Products. Foods.

[352] Kabacik S., Lowe D., Fransen L., Leonard M., Ang S.L., Whiteman C., Corsi S., Cohen H., Felton S., Bali R. (2022). The relationship between epigenetic age and the hallmarks of aging in human cells. Nat. Aging.

[353] Liu D., Aziz N.A., Pehlivan G., Breteler M.M.B. (2023). Cardiovascular correlates of epigenetic aging across the adult lifespan: A population-based study. Geroscience.

[354] Battaglia S., Tanaka M. (2026). Screen, Sample, Stratify: Biomarkers and Machine Learning Compress Dementia Pathways. Biomedicines.

[355] Higgins-Chen A.T., Thrush K.L., Wang Y., Minteer C.J., Kuo P.L., Wang M., Niimi P., Sturm G., Lin J., Moore A.Z. (2022). A computational solution for bolstering reliability of epigenetic clocks: Implications for clinical trials and longitudinal tracking. Nat. Aging.

[356] Shindo R., Tanifuji T., Okazaki S., Otsuka I., Shirai T., Mouri K., Horai T., Hishimoto A. (2023). Accelerated epigenetic aging and decreased natural killer cells based on DNA methylation in patients with untreated major depressive disorder. npj Aging.

[357] Lu A.T., Quach A., Wilson J.G., Reiner A.P., Aviv A., Raj K., Hou L., Baccarelli A.A., Li Y., Stewart J.D. (2019). DNA methylation GrimAge strongly predicts lifespan and healthspan. Aging.

[358] Yusupov N., Dieckmann L., Erhart M., Sauer S., Rex-Haffner M., Kopf-Beck J., Brückl T.M., Czamara D., Binder E.B. (2023). Transdiagnostic evaluation of epigenetic age acceleration and burden of psychiatric disorders. Neuropsychopharmacology.

[359] Han L.K.M., Aghajani M., Clark S.L., Chan R.F., Hattab M.W., Shabalin A.A., Zhao M., Kumar G., Xie L.Y., Jansen R. (2018). Epigenetic Aging in Major Depressive Disorder. Am. J. Psychiatry.

[360] Ma Z., Shen S., Hao M., Qin Y., Tang Y., Jin L., Wang F., Gong X. (2026). Accelerated epigenetic aging in males with mood disorders accompanied by insomnia. J. Affect. Disord..

[361] Nshanian M., Gruber J.J., Geller B.S., Chleilat F., Lancaster S.M., White S.M., Alexandrova L., Camarillo J.M., Kelleher N.L., Zhao Y. (2025). Short-chain fatty acid metabolites propionate and butyrate are unique epigenetic regulatory elements linking diet, metabolism and gene expression. Nat. Metab..

[362] Wang J., Zhao Q., Zhang S., Liu J., Fan X., Han B., Hou Y., Ai X. (2026). Microbial short chain fatty acids: Effective histone deacetylase inhibitors in immune regulation (Review). Int. J. Mol. Med..

[363] Thomas S.P., Denu J.M. (2021). Short-chain fatty acids activate acetyltransferase p300. eLife.

[364] Sanchez H.N., Moroney J.B., Gan H., Shen T., Im J.L., Li T., Taylor J.R., Zan H., Casali P. (2020). B cell-intrinsic epigenetic modulation of antibody responses by dietary fiber-derived short-chain fatty acids. Nat. Commun..

[365] McBride D.A., Dorn N.C., Yao M., Johnson W.T., Wang W., Bottini N., Shah N.J. (2023). Short-chain fatty acid-mediated epigenetic modulation of inflammatory T cells in vitro. Drug Deliv. Transl. Res..

[366] Ke Y., Li D., Zhao M., Liu C., Liu J., Zeng A., Shi X., Cheng S., Pan B., Zheng L. (2018). Gut flora-dependent metabolite Trimethylamine-N-oxide accelerates endothelial cell senescence and vascular aging through oxidative stress. Free Radic. Biol. Med..

[367] Li X., Li C., Zhang W., Wang Y., Qian P., Huang H. (2023). Inflammation and aging: Signaling pathways and intervention therapies. Signal Transduct. Target. Ther..

[368] Hwang H.J., Kim N., Herman A.B., Gorospe M., Lee J.S. (2022). Factors and Pathways Modulating Endothelial Cell Senescence in Vascular Aging. Int. J. Mol. Sci..

[369] Meier H.C.S., Mitchell C., Karadimas T., Faul J.D. (2023). Systemic inflammation and biological aging in the Health and Retirement Study. Geroscience.

[370] Teissier T., Boulanger E., Cox L.S. (2022). Interconnections between Inflammageing and Immunosenescence during Ageing. Cells.

[371] Picos A., Seoane N., Campos-Toimil M., Viña D. (2025). Vascular senescence and aging: Mechanisms, clinical implications, and therapeutic prospects. Biogerontology.

[372] Müller L., Di Benedetto S. (2024). Inflammaging, immunosenescence, and cardiovascular aging: Insights into long COVID implications. Front. Cardiovasc. Med..

[373] Li Q., Qian Z., Huang Y., Yang X., Yang J., Xiao N., Liang G., Zhang H., Fu Y., Lin Y. (2025). Mechanisms of endothelial senescence and vascular aging. Biogerontology.

[374] Donato A.J., Morgan R.G., Walker A.E., Lesniewski L.A. (2015). Cellular and molecular biology of aging endothelial cells. J. Mol. Cell. Cardiol..

[375] Sazdova I., Hadzi-Petrushev N., Keremidarska-Markova M., Stojchevski R., Sopi R., Shileiko S., Mitrokhin V., Gagov H., Avtanski D., Lubomirov L.T. (2024). SIRT-associated attenuation of cellular senescence in vascular wall. Mech. Ageing Dev..

[376] Zaccaria E., Klaassen T., Alleleyn A.M.E., Boekhorst J., Smokvina T., Kleerebezem M., Troost F.J. (2023). Endogenous small intestinal microbiome determinants of transient colonisation efficiency by bacteria from fermented dairy products: A randomised controlled trial. Microbiome.

[377] Padhi S., Sarkar P., Sahoo D., Rai A.K. (2025). Potential of fermented foods and their metabolites in improving gut microbiota function and lowering gastrointestinal inflammation. J. Sci. Food Agric..

[378] Bui G., Marco M.L. (2025). Impact of Fermented Dairy on Gastrointestinal Health and Associated Biomarkers. Nutr. Rev..

[379] Shahbazi R., Sharifzad F., Bagheri R., Alsadi N., Yasavoli-Sharahi H., Matar C. (2021). Anti-Inflammatory and Immunomodulatory Properties of Fermented Plant Foods. Nutrients.

[380] Maleki Sedgi F., Mozaffari N., Pashaei M.R., Hajizadeh-Sharafabad F. (2025). Effect of fermented soybean on metabolic outcomes, anthropometric indices, and body composition: A systematic review and meta-analysis of clinical trials. Food Funct..

[381] West L.M., Sabaté J., Nwachukwu I.D., Lee G.J., Sirirat R., Wright A., Rajaram S. (2025). Effects of Fermented Soy on Cognition in Older Adults: Outcomes of a Randomized, Controlled Trial. Nutrients.

[382] Kuettner A., Beck T., Drosch T., Kettering K., Heuschmid M., Burgstahler C., Claussen C.D., Kopp A.F., Schroeder S. (2005). Image quality and diagnostic accuracy of non-invasive coronary imaging with 16 detector slice spiral computed tomography with 188 ms temporal resolution. Heart.

[383] Lee M.J., Zhu J., An J.H., Lee S.E., Kim T.Y., Oh E., Kang Y.E., Chung W., Heo J.Y. (2022). A transcriptomic analysis of cerebral microvessels reveals the involvement of Notch1 signaling in endothelial mitochondrial-dysfunction-dependent BBB disruption. Fluids Barriers CNS.

[384] Ohbuchi M., Shibuta M., Tetsuka K., Sasaki-Iwaoka H., Oishi M., Shimizu F., Nagasaka Y. (2024). Modeling of Blood-Brain Barrier (BBB) Dysfunction and Immune Cell Migration Using Human BBB-on-a-Chip for Drug Discovery Research. Int. J. Mol. Sci..

[385] Ozgür B., Puris E., Brachner A., Appelt-Menzel A., Oerter S., Balzer V., Holst M.R., Christiansen R.F., Hyldig K., Buckley S.T. (2023). Characterization of an iPSC-based barrier model for blood-brain barrier investigations using the SBAD0201 stem cell line. Fluids Barriers CNS.

[386] Clarke E., Ferguson J., Collins C. (2023). Dietary assessment and metabolomic methodologies in feeding studies: A scoping review. Proc. Nutr. Soc..

[387] Clarke E.D., Ferguson J.J., Stanford J., Collins C.E. (2023). Dietary Assessment and Metabolomic Methodologies in Human Feeding Studies: A Scoping Review. Adv. Nutr..

[388] Fetsko A.R., Sebo D.J., Budzynski L.B., Scharbarth A., Taylor M.R. (2024). IL-1β disrupts the initiation of blood-brain barrier development by inhibiting endothelial Wnt/β-catenin signaling. iScience.

[389] Chen L.H., Canibe N., Curtasu M.V., Hedemann M.S. (2025). Untargeted metabolomics as a tool to assess the impact of dietary approaches on pig gut health: A review. J. Anim. Sci. Biotechnol..

[390] Yang K., Zeng L., Ge A., Yi Y., Wang S., Ge J. (2021). Exploring the Oxidative Stress Mechanism of Buyang Huanwu Decoction in Intervention of Vascular Dementia Based on Systems Biology Strategy. Oxid. Med. Cell. Longev..

[391] Zeilstra D., Younes J.A., Brummer R.J., Kleerebezem M. (2018). Perspective: Fundamental Limitations of the Randomized Controlled Trial Method in Nutritional Research: The Example of Probiotics. Adv. Nutr..

[392] Slykerman R.F., Davies N., Vlckova K., O’Riordan K.J., Bassett S.A., Dekker J., Schellekens H., Hyland N.P., Clarke G., Patterson E. (2025). Precision Psychobiotics for Gut-Brain Axis Health: Advancing the Discovery Pipelines to Deliver Mechanistic Pathways and Proven Health Efficacy. Microb. Biotechnol..

[393] Michaelis L., Berg L., Maier L. (2024). Confounder or Confederate? The Interactions Between Drugs and the Gut Microbiome in Psychiatric and Neurological Diseases. Biol. Psychiatry.

[394] Kyei-Baffour V.O., Vijaya A.K., Burokas A., Daliri E.B. (2025). Psychobiotics and the gut-brain axis: Advances in metabolite quantification and their implications for mental health. Crit. Rev. Food Sci. Nutr..

[395] Samantaray P., Saha S. (2026). Decoding the Microbial Diversity of Indian Fermented Foods: Integrating Ethnobiology, Multi-Omics and Functional Insights. Foods.

[396] Marco M.L., Heeney D., Binda S., Cifelli C.J., Cotter P.D., Foligné B., Gänzle M., Kort R., Pasin G., Pihlanto A. (2017). Health benefits of fermented foods: Microbiota and beyond. Curr. Opin. Biotechnol..

[397] Zheng X., Huang X., Liu Y., Sun Y., Zhang D., Liu X., Zhang J., Xia P., Ling J., Wang W. (2026). Current progress in production and identification of umami peptides from soybeans and soybean products: A review. Food Chem..

[398] Taylor A.M., Holscher H.D. (2020). A review of dietary and microbial connections to depression, anxiety, and stress. Nutr. Neurosci..

[399] Shawky E., Surendran S., El-Khair R.M.A. (2026). Fermented Vegetables as a Source of Psychobiotics: A Review of the Evidence for Mental Health Benefits. Probiot. Antimicrob. Proteins.

[400] Kim B., Hong V.M., Yang J., Hyun H., Im J.J., Hwang J., Yoon S., Kim J.E. (2016). A review of fermented foods with beneficial effects on brain and cognitive function. Prev. Nutr. Food Sci..

[401] Selhub E.M., Logan A.C., Bested A.C. (2014). Fermented foods, microbiota, and mental health: Ancient practice meets nutritional psychiatry. J. Physiol. Anthropol..

[402] Melini F., Melini V., Luziatelli F., Ficca A.G., Ruzzi M. (2019). Health-promoting components in fermented foods: An up-to-date systematic review. Nutrients.

[403] David L.A., Maurice C.F., Carmody R.N., Gootenberg D.B., Button J.E., Wolfe B.E., Ling A.V., Devlin A.S., Varma Y., Fischbach M.A. (2014). Diet rapidly and reproducibly alters the human gut microbiome. Nature.

[404] Sánchez-Villegas A., Delgado-Rodríguez M., Alonso A., Schlatter J., Lahortiga F., Serra Majem L., Martínez-González M.A. (2009). Association of the Mediterranean dietary pattern with the incidence of depression: The Seguimiento Universidad de Navarra/University of Navarra follow-up (SUN) cohort. Arch. Gen. Psychiatry.

[405] Bertin L., Facchin S., Barberio B., Maniero D., Lorenzon G., Cesaroni F., Zanconato M., Romanelli G., Francini-Pesenti F., Busetto L. (2026). Diet and Gut Microbiota in Inflammatory Bowel Disease: A Clinical and Nutritional Perspective. Pharmaceuticals.

[406] Goldsmith J.R., Sartor R.B. (2014). The role of diet on intestinal microbiota metabolism: Downstream impacts on host immune function and health, and therapeutic implications. J. Gastroenterol..

[407] McFarland L.V., Evans C.T., Goldstein E.J.C. (2018). Strain-Specificity and Disease-Specificity of Probiotic Efficacy: A Systematic Review and Meta-Analysis. Front. Med..

[408] Haiyan L., Dan W., Xiaochao W., Xiuxiu C., Ye L., Zhiguo C., Ting L. (2025). Efficacy of probiotic intervention in unmedicated depression: A systematic review and meta-analysis. Front. Psychiatry.

[409] Zhao S., Liang S., Tao J., Peng Y., Chen S., Wai H.K.F., Chung F.Y., Sin Z.Y., Wong M.K.L., Haqq A.M. (2025). Probiotics for adults with major depressive disorder compared with antidepressants: A systematic review and network meta-analysis. Nutr. Rev..

[410] Marco M.L., Cunningham M., Bischoff S.C., Clarke G., Delzenne N., Lewis J.D., Meisel M., Merenstein D., O’Toole P.W., Staudacher H.M. (2026). The International Scientific Association for Probiotics and Prebiotics (ISAPP) consensus statement on the definition and scope of gut health. Nat. Rev. Gastroenterol. Hepatol..

[411] Xie J., Bai S., He F., Xu K., Wang J., Ren Y., Chen J., Xie P. (2026). Gut Microbial-Related Alanine, Aspartate, and Glutamate Metabolism is Disordered in Peripheral and Central Tissues of Depressed Mice. Curr. Neuropharmacol..

[412] Kaur S., Bhandari N., Mahajan S., Mehta D., Chauhan S., Kumar V., Rohilla M., Mehta S., Dhankhar S. (2026). Molecular Pathways of Microbiota-derived Neuromodulation: An Integrative View. Curr. Neurovasc. Res..

[413] Zhang C., Zhang J., Liu D. (2021). Biochemical changes and microbial community dynamics during spontaneous fermentation of Zhacai, a traditional pickled mustard tuber from China. Int. J. Food Microbiol..

[414] Huang R., Fang Y., Zhong Y., Wang D., Lu W., Zhao H., Deng Y. (2025). Advancing fermentation science: Microbial dynamics, metabolomics, and safety in fermented vegetables. J. Futur. Foods.

[415] Świder O., Wójcicki M., Bujak M., Juszczuk-Kubiak E., Szczepańska M., Roszko M. (2021). Time Evolution of Microbial Composition and Metabolic Profile for Biogenic Amines and Free Amino Acids in a Model Cucumber Fermentation System Brined with 0.5% to 5.0% Sodium Chloride. Molecules.

[416] Wang X., Chen B., Fang X., Zhong Q., Liao Z., Wang J., Wu X., Ma Y., Li P., Feng X. (2024). Soy isoflavone-specific biotransformation product S-equol in the colon: Physiological functions, transformation mechanisms, and metabolic regulatory pathways. Crit. Rev. Food Sci. Nutr..

[417] Yuan Y., Yang Y., Xiao L., Qu L., Zhang X., Wei Y. (2023). Advancing Insights into Probiotics during Vegetable Fermentation. Foods.

[418] Tanaka M. (2025). Special Issue “Translating Molecular Psychiatry: From Biomarkers to Personalized Therapies. Int. J. Mol. Sci..

[419] Tanaka M. (2025). Parkinson’s Disease: Bridging Gaps, Building Biomarkers, and Reimagining Clinical Translation. Cells.

[420] Ruan Y., Lin H., Lu X., Lin Y., Sun J., Xu C., Zhou L., Cai Z., Chen X. (2024). Application and value of anxiety and depression scale in patients with functional dyspepsia. BMC Psychol..

[421] Grisanzio K.A., Goldstein-Piekarski A.N., Wang M.Y., Rashed Ahmed A.P., Samara Z., Williams L.M. (2018). Transdiagnostic Symptom Clusters and Associations With Brain, Behavior, and Daily Function in Mood, Anxiety, and Trauma Disorders. JAMA Psychiatry.

[422] Zarate D., Stavropoulos V., Ball M., de Sena Collier G., Jacobson N.C. (2022). Exploring the digital footprint of depression: A PRISMA systematic literature review of the empirical evidence. BMC Psychiatry.

[423] Xiao W., Li J., Gao X., Yang H., Su J., Weng R., Gao Y., Ni W., Gu Y. (2022). Involvement of the gut-brain axis in vascular depression via tryptophan metabolism: A benefit of short chain fatty acids. Exp. Neurol..

[424] Agus A., Clément K., Sokol H. (2021). Gut microbiota-derived metabolites as central regulators in metabolic disorders. Gut.

[425] Jacobs J.P., Lagishetty V., Hauer M.C., Labus J.S., Dong T.S., Toma R., Vuyisich M., Naliboff B.D., Lackner J.M., Gupta A. (2023). Multi-omics profiles of the intestinal microbiome in irritable bowel syndrome and its bowel habit subtypes. Microbiome.

[426] Madison A.A., Andridge R., Padin A.C., Wilson S., Bailey M.T., Alfano C.M., Povoski S.P., Lipari A.M., Agnese D.M., Carson W.E. (2020). Endotoxemia coupled with heightened inflammation predicts future depressive symptoms. Psychoneuroendocrinology.

[427] Morena D., Lippi M., Scopetti M., Turillazzi E., Fineschi V. (2025). Leaky Gut Biomarkers as Predictors of Depression and Suicidal Risk: A Systematic Review and Meta-Analysis. Diagnostics.

[428] Ali M.M., Mahmoud A.M., Le Master E., Levitan I., Phillips S.A. (2019). Role of matrix metalloproteinases and histone deacetylase in oxidative stress-induced degradation of the endothelial glycocalyx. Am. J. Physiol. Heart Circ. Physiol..

[429] Groten S.A., Smit E.R., Janssen E.F.J., van den Eshof B.L., van Alphen F.P.J., van der Zwaan C., Meijer A.B., Hoogendijk A.J., van den Biggelaar M. (2023). Multi-omics delineation of cytokine-induced endothelial inflammatory states. Commun. Biol..

[430] Michelson A.D., Barnard M.R., Krueger L.A., Valeri C.R., Furman M.I. (2001). Circulating monocyte-platelet aggregates are a more sensitive marker of in vivo platelet activation than platelet surface P-selectin: Studies in baboons, human coronary intervention, and human acute myocardial infarction. Circulation.

[431] Pluta K., Porębska K., Urbanowicz T., Gąsecka A., Olasińska-Wiśniewska A., Targoński R., Krasińska A., Filipiak K.J., Jemielity M., Krasiński Z. (2022). Platelet-Leucocyte Aggregates as Novel Biomarkers in Cardiovascular Diseases. Biology.

[432] Solari F.A., Krahn D., Swieringa F., Verhelst S., Rassaf T., Tasdogan A., Zahedi R.P., Lorenz K., Renné T., Heemskerk J.W.M. (2023). Multi-omics approaches to study platelet mechanisms. Curr. Opin. Chem. Biol..

[433] Belsky D.W., Caspi A., Corcoran D.L., Sugden K., Poulton R., Arseneault L., Baccarelli A., Chamarti K., Gao X., Hannon E. (2022). DunedinPACE, a DNA methylation biomarker of the pace of aging. eLife.

[434] Hillary R.F., Trejo-Banos D., Kousathanas A., McCartney D.L., Harris S.E., Stevenson A.J., Patxot M., Ojavee S.E., Zhang Q., Liewald D.C. (2020). Multi-method genome- and epigenome-wide studies of inflammatory protein levels in healthy older adults. Genome Med..

[435] Liu Z., Leung D., Thrush K., Zhao W., Ratliff S., Tanaka T., Schmitz L.L., Smith J.A., Ferrucci L., Levine M.E. (2020). Underlying features of epigenetic aging clocks in vivo and in vitro. Aging Cell.

[436] Xiong H., Yu L.X., Qu H. (2013). Batch-to-batch quality consistency evaluation of botanical drug products using multivariate statistical analysis of the chromatographic fingerprint. AAPS PharmSciTech.

[437] Kirwan J.A., Broadhurst D.I., Davidson R.L., Viant M.R. (2013). Characterising and correcting batch variation in an automated direct infusion mass spectrometry (DIMS) metabolomics workflow. Anal. Bioanal. Chem..

[438] Lugli G.A., Mangifesta M., Mancabelli L., Milani C., Turroni F., Viappiani A., van Sinderen D., Ventura M. (2019). Compositional assessment of bacterial communities in probiotic supplements by means of metagenomic techniques. Int. J. Food Microbiol..

[439] Roach J., Mital R., Haffner J.J., Colwell N., Coats R., Palacios H.M., Liu Z., Godinho J.L.P., Ness M., Peramuna T. (2024). Microbiome metabolite quantification methods enabling insights into human health and disease. Methods.

[440] Chelakkot C., Ghim J., Ryu S.H. (2018). Mechanisms regulating intestinal barrier integrity and its pathological implications. Exp. Mol. Med..

[441] Wienkamp A.K., Erpenbeck L., Rossaint J. (2022). Platelets in the NETworks interweaving inflammation and thrombosis. Front. Immunol..

[442] Rosell A., Martinod K., Mackman N., Thålin C. (2022). Neutrophil extracellular traps and cancer-associated thrombosis. Thromb. Res..

[443] Xia F., Cui P., Liu L., Chen J., Zhou Q., Wang Q., Zhou H. (2024). Quantification of gut microbiome metabolites using chemical isotope derivatization strategy combined with LC-MS/MS: Application in neonatal hypoxic-ischemic encephalopathy rat model. J. Pharm. Biomed. Anal..

[444] Heumel S., de Rezende Rodovalho V., Urien C., Specque F., Brito Rodrigues P., Robil C., Delval L., Sencio V., Descat A., Deruyter L. (2024). Shotgun metagenomics and systemic targeted metabolomics highlight indole-3-propionic acid as a protective gut microbial metabolite against influenza infection. Gut Microbes.

[445] Wang Q., Chen C., Zuo S., Cao K., Li H. (2023). Integrative analysis of the gut microbiota and faecal and serum short-chain fatty acids and tryptophan metabolites in patients with cirrhosis and hepatic encephalopathy. J. Transl. Med..

[446] Barbara G., Barbaro M.R., Fuschi D., Palombo M., Falangone F., Cremon C., Marasco G., Stanghellini V. (2021). Inflammatory and Microbiota-Related Regulation of the Intestinal Epithelial Barrier. Front. Nutr..

[447] Meyer F., Wendling D., Demougeot C., Prati C., Verhoeven F. (2023). Cytokines and intestinal epithelial permeability: A systematic review. Autoimmun. Rev..

[448] Zuo L., Kuo W.T., Turner J.R. (2020). Tight Junctions as Targets and Effectors of Mucosal Immune Homeostasis. Cell. Mol. Gastroenterol. Hepatol..

[449] Dmytriv T.R., Storey K.B., Lushchak V.I. (2024). Intestinal barrier permeability: The influence of gut microbiota, nutrition, and exercise. Front. Physiol..

[450] Joffre J., Hellman J. (2021). Oxidative Stress and Endothelial Dysfunction in Sepsis and Acute Inflammation. Antioxid. Redox Signal..

[451] Scioli M.G., Storti G., D’Amico F., Rodríguez Guzmán R., Centofanti F., Doldo E., Céspedes Miranda E.M., Orlandi A. (2020). Oxidative Stress and New Pathogenetic Mechanisms in Endothelial Dysfunction: Potential Diagnostic Biomarkers and Therapeutic Targets. J. Clin. Med..

[452] Dri E., Lampas E., Lazaros G., Lazarou E., Theofilis P., Tsioufis C., Tousoulis D. (2023). Inflammatory Mediators of Endothelial Dysfunction. Life.

[453] Hernandez-Navarro I., Botana L., Diez-Mata J., Tesoro L., Jimenez-Guirado B., Gonzalez-Cucharero C., Alcharani N., Zamorano J.L., Saura M., Zaragoza C. (2024). Replicative Endothelial Cell Senescence May Lead to Endothelial Dysfunction by Increasing the BH2/BH4 Ratio Induced by Oxidative Stress, Reducing BH4 Availability, and Decreasing the Expression of eNOS. Int. J. Mol. Sci..

[454] Theofilis P., Sagris M., Oikonomou E., Antonopoulos A.S., Siasos G., Tsioufis C., Tousoulis D. (2021). Inflammatory Mechanisms Contributing to Endothelial Dysfunction. Biomedicines.

[455] Montenont E., Rondina M.T., Campbell R.A. (2019). Altered functions of platelets during aging. Curr. Opin. Hematol..

[456] Elaskalani O., Abdol Razak N.B., Metharom P. (2018). Neutrophil extracellular traps induce aggregation of washed human platelets independently of extracellular DNA and histones. Cell Commun. Signal..

[457] Reyes-Díaz A., Mata-Haro V., Hernández J., González-Córdova A.F., Hernández-Mendoza A., Reyes-Díaz R., Torres-Llanez M.J., Beltrán-Barrientos L.M., Vallejo-Cordoba B. (2018). Milk Fermented by Specific *Lactobacillus* Strains Regulates the Serum Levels of IL-6, TNF-α and IL-10 Cytokines in a LPS-Stimulated Murine Model. Nutrients.

[458] Martini M.C., Lerebours E.C., Lin W.J., Harlander S.K., Berrada N.M., Antoine J.M., Savaiano D.A. (1991). Strains and species of lactic acid bacteria in fermented milks (yogurts): Effect on in vivo lactose digestion. Am. J. Clin. Nutr..

[459] Cao P., Gao M., Huang D., Xu X., Liu Z., Liu Q., Lu Y., Pan F., Li Z., Sun J. (2025). Analysis of Five Biogenic Amines in Foods on the Chinese Market and Estimation of Acute Histamine Exposure from Fermented Foods in the Chinese Population. Foods.

[460] Darnay L., Miklós G., Lőrincz A., Szakmár K., Pásztor-Huszár K., Laczay P. (2022). Possible inhibitory effect of microbial transglutaminase on the formation of biogenic amines during Trappist cheese ripening. Food Addit. Contam. Part A.

[461] Abdel Tawab F.I., Abd Elkadr M.H., Sultan A.M., Hamed E.O., El-Zayat A.S., Ahmed M.N. (2023). Probiotic potentials of lactic acid bacteria isolated from Egyptian fermented food. Sci. Rep..

[462] Wang Y., An M., Lv Y., Hou Y., Li Z., Dai J., Ni W., Hu S. (2026). Genomic and functional characterization of probiotic strains from traditional fermented dairy products in alleviating dextran sulfate sodium-induced colitis. J. Dairy. Sci..

[463] Kent D.M., Paulus J.K., van Klaveren D., D’Agostino R., Goodman S., Hayward R., Ioannidis J.P.A., Patrick-Lake B., Morton S., Pencina M. (2020). The Predictive Approaches to Treatment effect Heterogeneity (PATH) Statement. Ann. Intern. Med..

[464] Jardon K.M., Canfora E.E., Goossens G.H., Blaak E.E. (2022). Dietary macronutrients and the gut microbiome: A precision nutrition approach to improve cardiometabolic health. Gut.

[465] Suneson K., Grudet C., Ventorp F., Malm J., Asp M., Westrin Å., Lindqvist D. (2023). An inflamed subtype of difficult-to-treat depression. Prog. Neuropsychopharmacol. Biol. Psychiatry.

[466] Sigala-Robles R., Santiago-López L., Hernández-Mendoza A., Vallejo-Cordoba B., Mata-Haro V., Wall-Medrano A., González-Córdova A.F. (2022). Peptides, Exopolysaccharides, and Short-Chain Fatty Acids from Fermented Milk and Perspectives on Inflammatory Bowel Diseases. Dig. Dis. Sci..

[467] Kim H.Y., Park E.S., Choi Y.S., Park S.J., Kim J.H., Chang H.K., Park K.Y. (2022). Kimchi improves irritable bowel syndrome: Results of a randomized, double-blind placebo-controlled study. Food Nutr. Res..

[468] Nielsen E.S., Garnås E., Jensen K.J., Hansen L.H., Olsen P.S., Ritz C., Krych L., Nielsen D.S. (2018). Lacto-fermented sauerkraut improves symptoms in IBS patients independent of product pasteurisation—A pilot study. Food Funct..

[469] Garnås E. (2023). Fermented Vegetables as a Potential Treatment for Irritable Bowel Syndrome. Curr. Dev. Nutr..

[470] Jung S.M., Kaur A., Amen R.I., Oda K., Rajaram S., Sabatè J., Haddad E.H. (2023). Effect of the Fermented Soy Q-CAN(^®^) Product on Biomarkers of Inflammation and Oxidation in Adults with Cardiovascular Risk, and Canonical Correlations between the Inflammation Biomarkers and Blood Lipids. Nutrients.

[471] Watanabe K., Igarashi M., Li X., Nakatani A., Miyamoto J., Inaba Y., Sutou A., Saito T., Sato T., Tachibana N. (2018). Dietary soybean protein ameliorates high-fat diet-induced obesity by modifying the gut microbiota-dependent biotransformation of bile acids. PLoS ONE.

[472] Li T., Han K., Feng G., Guo J., Wan Z., Yang X. (2024). Condensation of Soy Protein Peptides Contributes to Sequester Bile Acids and Mitigate LPS-Induced Inflammation. J. Agric. Food Chem..

[473] Shimizu S., Hirano K., Saito T., Akiyama H., Shimizu K., Honda H. (2025). Identification of Soy-Derived Peptides With Micelle Disruption Activity of Secondary Bile Acids. Food Sci. Nutr..

[474] Hornero-Ramirez H., Morisette A., Marcotte B., Penhoat A., Lecomte B., Panthu B., Lessard Lord J., Thirion F., Van-Den-Berghe L., Blond E. (2025). Multifunctional dietary approach reduces intestinal inflammation in relation with changes in gut microbiota composition in subjects at cardiometabolic risk: The SINFONI project. Gut Microbes.

[475] Chen Z., Liang N., Zhang H., Li H., Guo J., Zhang Y., Chen Y., Wang Y., Shi N. (2024). Resistant starch and the gut microbiome: Exploring beneficial interactions and dietary impacts. Food Chem. X.

[476] Mohammad S., Thiemermann C. (2020). Role of Metabolic Endotoxemia in Systemic Inflammation and Potential Interventions. Front. Immunol..

[477] Serebruany V.L., Glassman A.H., Malinin A.I., Nemeroff C.B., Musselman D.L., van Zyl L.T., Finkel M.S., Krishnan K.R., Gaffney M., Harrison W. (2003). Platelet/endothelial biomarkers in depressed patients treated with the selective serotonin reuptake inhibitor sertraline after acute coronary events: The Sertraline AntiDepressant Heart Attack Randomized Trial (SADHART) Platelet Substudy. Circulation.

[478] van Zyl L.T., Lespérance F., Frasure-Smith N., Malinin A.I., Atar D., Laliberté M.A., Serebruany V.L. (2009). Platelet and endothelial activity in comorbid major depression and coronary artery disease patients treated with citalopram: The Canadian Cardiac Randomized Evaluation of Antidepressant and Psychotherapy Efficacy Trial (CREATE) biomarker sub-study. J. Thromb. Thrombolysis.

[479] Pereira J., Massardo T., Saez C.G., Olivares N., Valenzuela J.G., Risco L., Veliz J., Spuler J., Castro G., Falloux E. (2018). Endothelial dysfunction and platelet activation in major depressive disorder: Association with brain perfusion abnormalities. Blood.

